# Supplementary material for: Sexually transmitted infections amongst men who have sex with men (MSM) in South Africa
Source: PLOS Glob Public Health. 2023 Apr 5;3(4):e0001782. doi: 10.1371/journal.pgph.0001782 (PMC10075439; doi:10.1371/journal.pgph.0001782)
Supplement: S1 Protocol — Inclusion and exclusion for HVTN 702 clinical trial (page 92–96). Available from: https://clinicaltrials.gov/ProvidedDocs/49/NCT02968849/Prot_ICF_000.pdf. (PDF) [file pgph.0001782.s001.pdf]

HIV VACCINE
TRIALS NETWORK

PROTOCOL

HVTN 702

A pivotal phase 2b/3 multi-site, randomized, double-blind, placebo-controlled clinical trial to evaluate the safety and efficacy of ALVAC-HIV (vCP2438) and Bivalent Subtype C gp120/MF59 in preventing HIV-1 infection in adults in South Africa

DAIDS DOCUMENT ID 12059

CLINICAL TRIAL SPONSORED BY

Division of AIDS (DAIDS)
National Institute of Allergy and Infectious Diseases (NIAID)
National Institutes of Health (NIH)
Department of Health and Human Services (DHHS)
Bethesda, Maryland, USA

STUDY PRODUCT(S) PROVIDED BY

Sanofi Pasteur
Swiftwater, Pennsylvania, USA

GlaxoSmithKline Biologicals, S.A. (GSK)
Rixensart, Belgium

May 20, 2020
HVTN 702
Version 4.0

On 23 January 2020, in reviewing cumulative unblinded data for HVTN 702, the NIAID HIV Vaccine Data Safety and Monitoring Board (DSMB) found that pre-established criteria for vaccine non-efficacy had been met. Accordingly, the DSMB recommended halting vaccinations. The HVTN 702 Oversight Group (OG) concurred with this DSMB recommendation and vaccinations in HVTN 702 were stopped as of 3 February 2020. The OG further determined that all HVTN 702 participants who have not yet completed their clinic visits schedules should be followed for safety for 12 months after the last vaccination they received.

Since no more participants will be vaccinated at Visit 11 (Month 18), Visit 12 (Month 18.5), whose purpose was evaluation of safety and immunogenicity following the vaccination at Visit 11, has been removed from the schedule. The visit schedule for the 12-month follow-up period for all participants remains otherwise unchanged.

HVTN 702 had an adaptive two-stage design. Stage 1 entailed following all participants for 24 months post-enrollment. Stage 2 was to be conducted if the vaccine was found to be efficacious at the end of Stage 1 and entailed following all participants for an additional 12 months to 36 months post-enrollment. Because the criteria for vaccine non-efficacy were met during Stage 1, Stage 2 of the trial will not occur.

All participants have been unblinded to their treatment assignments. Follow-up will continue in an unblinded fashion.

Procedures specified for remaining follow-up visits have been revised. Because all remaining participants are now at least 3 months post-vaccination, pregnancy tests and contraception assessments have been removed from the list of procedures. Because the primary assessment of vaccine efficacy has been completed, further laboratory testing for ARV is no longer needed. Because all study participants have been unblinded, the “Outside testing and belief questionnaire” is no longer needed. Remaining procedures, including HIV testing, physical examinations, STI testing/treatment, recording of concomitant medications, AE assessment, social impact assessment, and specimen collection for safety and selected endpoint evaluations remain unchanged.

The revised study procedures are shown in new Section 9.4.1 and in new Appendix M and Appendix N.

The visit schedule and procedures for HIV-infected participants (Schedule 2) remain unchanged (see Appendix G and Appendix I).

New Appendix K contains an addendum to the sample consent form that updates study information and new Appendix L contains tables of procedures for the consent addendum.

Contents

1 Ethical considerations 6
2 IRB/EC review considerations 8
2.1 Minimized risks to participants 8
2.2 Reasonable risk/benefit balance 9
2.3 Equitable subject selection 9
2.4 Appropriate informed consent 9
2.5 Adequate safety monitoring 10
2.6 Protect privacy/confidentiality 10
3 Overview 11
3.1 Protocol Team 15
4 Background 17
4.1 Burden of HIV and rationale for HIV vaccine development 17
4.2 Rationale for trial concept 17
4.3 ALVAC-HIV (vCP2438) 29
4.4 Bivalent Subtype C gp120/MF59 31
4.5 Trial design rationale 33
4.6 Combination prevention of HIV acquisition 38
4.7 Nonclinical studies 39
4.8 Plans for future product development and testing 42
4.9 Clinical studies 43
4.10 Potential risks of study products and administration 67
5 Objectives and endpoints 68
5.1 Primary objectives and endpoints 68
5.2 Secondary objectives and endpoints 69
5.3 Exploratory objectives 70
6 Statistical considerations 72
6.1 Definitions of study cohorts for analysis 72
6.2 Objectives 73
6.3 Study cohorts utilized for evaluations of study endpoints 73
6.4 Accrual and sample size 74
6.5 Monitoring of trial 76
6.6 Assessment of PrEP use 83
6.7 Randomization of treatment assignments 84
6.8 Blinding 84
6.9 Statistical analysis 85
7 Selection and withdrawal of participants 92
7.1 Inclusion criteria 92
7.2 Exclusion criteria 94
7.3 Participant departure from vaccination schedule or withdrawal 96
8 Study product preparation and administration 99
8.1 Vaccine regimen 99
8.2 Study product formulation 99
8.3 Preparation of study products 100

8.4 Administration 103
8.5 Acquisition of study products 103
8.6 Pharmacy records 103
8.7 Final disposition of study products 104

9 Clinical procedures 105
9.1 Informed consent 105
9.2 Pre-enrollment procedures 107
9.3 Enrollment and vaccination visits (pre-unblinding) 108
9.4 Follow-up visits (pre-unblinding) 110
9.5 Stool sampling 112
9.6 Procedures for HIV-infected participants 112
9.7 HIV counseling and testing 113
9.8 Pregnancy prevention assessment 115
9.9 Assessments of reactogenicity 115
9.10 Visit windows and missed visits 116
9.11 Early termination visit 117
9.12 Pregnancy 117

10 HIV-1 infection assessment and clinical response 118
10.1 HIV-1 symptom assessment 118
10.2 HIV-1 screening test (prior to randomization) 118
10.3 HIV-1 *testing* postvaccination 118
10.4 Endpoint adjudication 119
10.5 HIV-1 infection during the study 119
10.6 Medical care for participants who become HIV-1–infected 120

11 Laboratory 121
11.1 HVTN CRS laboratory procedures 121
11.2 Total blood volume 121
11.3 Assay timepoints 121
11.4 Endpoint assays: humoral 121
11.5 Endpoint assays: cellular 122
11.6 Viral sequencing 122
11.7 ARV detection 122
11.8 Genotyping 123
11.9 Laboratory Center assay portfolio 123
11.10 Exploratory studies 123
11.11 Other use of stored specimens 124
11.12 Biohazard containment 124

12 Safety monitoring and safety review 125
12.1 Safety monitoring and oversight 125
12.2 Safety reporting 126
12.3 AEs requiring immediate PSRT notification and prompt PSRT AE review 129
12.4 Review of cumulative safety data 131
12.5 Study termination 132

13 Protocol conduct 133

13.1 Protocol governance 134
13.2 Social impacts 135
13.3 Compliance with NIH guidelines for research involving products containing recombinant DNA 135
13.4 Specific regulatory considerations for Republic of South Africa 136
13.5 Emergency communication with study participants 136

14 Version history 137

15 Document references (other than literature citations) 144

16 Acronyms and abbreviations 147

17 Literature cited 151

Appendix A Sample informed consent form 163

Appendix B Approved birth control methods (for sample informed consent form) 185

Appendix C Approved birth control methods for transgender men (for sample informed consent form) 186

Appendix D Sample consent form for use of samples and information in other studies 187

Appendix E Tables of procedures (for sample informed consent form) 192

Appendix F Schedule 1: Laboratory procedures for HIV-uninfected participants 194

Appendix G Schedule 2: Laboratory procedures for HIV-infected participants 196

Appendix H Schedule 1: Procedures at HVTN CRS for HIV-uninfected participants (continued on next page) 198

Appendix I Schedule 2: Procedures at HVTN CRS for HIV-infected participants 200

Appendix J Adverse events of special interest 201

Appendix K Sample addendum to informed consent form (post-unblinding) 202

Appendix L Tables of procedures (for addendum to sample informed consent form) ..208

Appendix M Schedule 3: Follow-up laboratory procedures for HIV-uninfected participants (post-unblinding) 209

Appendix N Schedule 3: Follow-up procedures at HVTN CRS for HIV-uninfected participants (post-unblinding) 211

Appendix O Protocol Signature Page 212

1 Ethical considerations

Multiple candidate HIV vaccines will need to be studied simultaneously in different populations around the world before a successful HIV preventive vaccine is found. It is critical that universally accepted ethical guidelines are followed at all sites involved in the conduct of these clinical trials. The HIV Vaccine Trials Network (HVTN) has addressed ethical concerns in the following ways:

- HVTN trials are designed and conducted to enhance the knowledge base necessary to find a preventive vaccine, using methods that are scientifically rigorous and valid, and in accordance with Good Clinical Practice (GCP) guidelines.

- HVTN scientists and operational staff incorporate the philosophies underlying major codes (1-3), declarations, and other guidance documents relevant to human subjects research into the design and conduct of HIV vaccine clinical trials.

- HVTN scientists and operational staff are committed to substantive community input—into the planning, conduct, and follow-up of its research—to help ensure that locally appropriate cultural and linguistic needs of study populations are met. Community Advisory Boards (CAB) are required by DAIDS and supported at all HVTN research sites to ensure community input, in accordance with Good Participatory Practices (GPP) and all local and national guidelines. The HVTN leadership is aware of the *Guidelines for Good Clinical Practice in the Conduct of Clinical Trials with Human Participants in South Africa* Section 2.4 "Communication and Community Involvement" and the South African Medical Research Council's *Guidelines on Ethics for Medical Research: HIV Preventive Vaccine Research* (particularly Section 5, Community Participation) and works to implement both guidelines generally and those sections specifically.

- HVTN clinical trial staff counsel study participants routinely on how to reduce HIV risk. Participants who become HIV infected during the trial are provided counseling on notifying their partners and about HIV infection according to local guidelines. Staff members will also counsel them about reducing their risk of transmitting HIV to others.

- The HVTN requires that all international HVTN sites lacking national plans for providing antiretroviral therapy (ART) develop plans for the care and treatment of participants who acquire HIV infection during a trial. Each plan is developed in consultation with representatives of host countries, communities from which potential trial participants will be drawn, sponsors, and the HVTN. Participants will be referred to agreed-upon programs for ART provision as soon as the participant is willing and able to attend. If a

program is not available at a site and ART is needed, a privately established fund will be used to pay for access to treatment to the fullest extent possible.

- The HVTN is committed to ensuring that all trial participants receive access to the highest standard of prevention, which may include, but is not limited to, risk reduction counseling, provision of male and female condoms, diagnostic testing and access to treatment for sexually transmitted infections (STIs), information regarding male circumcision and referral to services that can provide male circumcision when indicated, and appropriate referrals to pre- and postexposure prophylaxis (PrEP and PEP) according to national and/or local guidelines.

- The HVTN provides training so that all participating sites similarly ensure fair participant selection, protect the privacy of research participants, and obtain meaningful informed consent. During the study, participants will have their wellbeing monitored, and to the fullest extent possible, their privacy protected. Participants may withdraw from the study at any time.

- Prior to implementation, HVTN trials are rigorously reviewed by scientists who are not involved in the conduct of the trials under consideration.

- HVTN trials are reviewed by local and national regulatory bodies and are conducted in compliance with all applicable national and local regulations.

- The HVTN designs its research to minimize risk and maximize benefit to both study participants and their local communities. For example, HVTN protocols provide enhancement of participants' knowledge of HIV and HIV prevention, as well as counseling, guidance, and assistance with any social impacts that may result from research participation. HVTN protocols also include careful medical review of each research participant's health conditions and reactions to study products while in the study.

- HVTN research aims to benefit local communities by directly addressing the health and HIV prevention needs of those communities and by strengthening the capacity of the communities through training, support, shared knowledge, and equipment. Researchers involved in HVTN trials are able to conduct other critical research in their local research settings.

- The HVTN recognizes the importance of institutional review and values the role of in country Institutional Review Boards (IRBs) and Ethics Committees (ECs) as custodians responsible for ensuring the ethical conduct of research in each setting.

2 IRB/EC review considerations

US Food and Drug Administration (FDA) and other US federal regulations require IRBs/ECs to ensure that certain requirements are satisfied on initial and continuing review of research (Title 45, Code of Federal Regulations (CFR), Part 46.111(a) 1-7; 21 CFR 56.111(a) 1-7). Each HVTN Investigator welcomes IRB/EC questions or concerns regarding these research requirements.

This trial is being conducted exclusively in South Africa, with funding from the US NIH. Due to this, the trial is subject to both US and South African regulations and guidelines on the protection of human research subjects and ethical research conduct. These research regulations and guidelines are based on ethical principles of respect for persons, beneficence and nonmaleficence, and justice. Where there is a conflict in regulations or guidelines, the regulation or guideline providing the maximum protection of human research subjects will be followed.

In compliance with the Guidelines For Good Practice In The Conduct Of Clinical Trials In Human Participants In South Africa ("South African GCPs"), each research location in South Africa has a South African-based Principal Investigator (PI) who is qualified to conduct (and supervise the conduct of) the research; and the research addresses an important South African health need for an HIV vaccine in line with the national strategic plan for South Africa and the national South African HIV vaccine plan. In addition, the investigators take responsibility for the conduct of the study and the control of the study products, including obtaining all appropriate South African regulatory and ethical reviews of the research. Each participating site has a standard operating procedure for ensuring that participants have the necessary information to make a decision whether or not to consent to the research. The sections below address each of the review concerns by IRBs/ECs regarding how the research will be conducted.

2.1 Minimized risks to participants

45 CFR 46.111 (a) 1 and 21 CFR 56.111 (a) 1: Risks to subjects are minimized.

This protocol minimizes risks to participants by (a) correctly and promptly informing participants about risks so that they can join in partnership with the researcher in recognizing and reporting harms; (b) respecting local/national blood draw limits; (c) performing direct observation of participants postvaccination and collecting information regarding side effects for several days postvaccination; (d) having staff properly trained in administering study procedures that may cause physical harm or psychological distress, such as blood draws, vaccinations, HIV testing and counseling and HIV risk reduction counseling; (e) providing HIV risk reduction counseling and checking on contraception use (for women); and (f) providing safety monitoring.

2.2 Reasonable risk/benefit balance

45 CFR 46.111(a) 2 and 21 CFR 56.111(a) 2: Risks to subjects are reasonable in relation to anticipated benefits, if any, to subjects, and the importance of the knowledge that may reasonably be expected to result.

In all public health research, the risk-benefit ratio may be difficult to assess because the benefits to a healthy participant are not as apparent as they would be in treatment protocols, where a study participant may be ill and may have exhausted all conventional treatment options. However, this protocol is designed to minimize the risks to participants while maximizing the potential value of the knowledge it is designed to generate.

2.3 Equitable subject selection

45 CFR 46.111 (a) 3 and 21 CFR 56.111 (a) 3: Subject selection is equitable

This protocol has specific inclusion and exclusion criteria for investigators to follow in admitting participants into the protocol. Participants are selected because of these criteria and not because of positions of vulnerability or privilege. Investigators are required to maintain screening and enrollment logs to document volunteers who screened into and out of the protocol and for what reasons.

2.4 Appropriate informed consent

45 CFR 46.111 (a) 4 & 5 and 21 CFR 56.111 (a) 4 & 5: Informed consent is sought from each prospective subject or the subject's legally authorized representative as required by 45 CFR 46.116 and 21 CFR Part 50; informed consent is appropriately documented as required by 45 CFR 46.117 and 21 CFR 50.27

The protocol specifies that informed consent must be obtained before any study procedures are initiated and assessed throughout the trial (Section 9.1). Each site is provided training in informed consent by the HVTN as part of its entering the HVTN. The HVTN requires a signed consent document for documentation, in addition to chart notes or a consent checklist.

2.5 Adequate safety monitoring

45 CFR 46.111 (a) 6 and 21 CFR 56.111 (a) 6: There is adequate provision for monitoring the data collected to ensure the safety of subjects.

This protocol has extensive safety monitoring in place (Section 12). Safety is monitored daily by HVTN Core and routinely by the HVTN 702 Protocol Safety Review Team (PSRT). In addition, a Data and Safety Monitoring Board (DSMB) periodically reviews study data.

2.6 Protect privacy/confidentiality

45 CFR 46.111 (a) 7 and 21 CFR 56.111 (a) 7: There are adequate provisions to protect the privacy of subjects and maintain the confidentiality of data.

Privacy refers to an individual’s right to be free from unauthorized or unreasonable intrusion into his/her private life and the right to control access to individually identifiable information about him/her. The term “privacy” concerns research participants or potential research participants as individuals whereas the term “confidentiality” is used to refer to the treatment of information about those individuals. This protocol respects the privacy of participants by informing them about who will have access to their personal information and study data (Appendix A). The privacy of participants is protected by assigning unique identifiers in place of the participant’s name on study data and specimens. In addition, each staff member at each study site in this protocol signs a Confidentiality Agreement with the HVTN and each study site participating in the protocol is required to have a standard operating procedure on how the staff members will protect the confidentiality of study participants.

3 Overview

Title

A pivotal phase 2b/3 multi-site, randomized, double-blind, placebo-controlled clinical trial to evaluate the safety and efficacy of ALVAC-HIV (vCP2438) and Bivalent Subtype C gp120/MF59 in preventing HIV-1 infection in adults in South Africa

Primary objective(s)

Primary objective 1:

To evaluate the vaccine efficacy (VE) of ALVAC-HIV (vCP2438) + Bivalent Subtype C gp120/MF59 for the prevention of HIV infection in HIV-seronegative South African adults over 24 months from enrollment

Per the 23 January 2020 DSMB finding that monitoring boundaries for non-efficacy have been met, Primary objective 1 has been superseded by Primary objective 3 below.

Primary objective 2:

To evaluate the safety and tolerability of ALVAC-HIV (vCP2438) + Bivalent Subtype C gp120/MF59 in adults in South Africa

Primary objective 3:

To evaluate the effect of ALVAC-HIV (vCP2438) +Bivalent Subtype C gp120/MF59 vaccination on HIV acquisition in HIV-seronegative African adults

Study products and routes of administration

- ALVAC-HIV (vCP2438) expresses the gene products ZM96 *gp120* (clade C strain) linked to the sequences encoding the HIV-1 transmembrane anchor (TM) sequence of *gp41* (28 amino acids clade B LAI strain) and *gag* and *pro* (clade B LAI strain). It is formulated as a lyophilized vaccine for injection at a dose > 10^6 cell culture infectious dose 50% ($CCID_{50}$) and < 1×10^8 $CCID_{50}$ (nominal dose of 10^7 $CCID_{50}$) and is reconstituted with 1 mL of sterile sodium chloride solution (NaCl 0.4%) administered by intramuscular (IM) injection as a single dose

- Bivalent Subtype C gp120/MF59: subtype C TV1.C gp120 Env and 1086.C gp120 Env proteins, each at a dose of 100 mcg, mixed with MF59 adjuvant (an oil-in-water emulsion) and delivered as a 0.5 mL IM injection

- ALVAC-HIV placebo: Sodium Chloride for injection, 0.9% delivered as a 1 mL IM injection

- Bivalent subtype C gp120/MF59 placebo: Sodium Chloride for injection, 0.9%, delivered as a 0.5 mL IM injection

Table 3-1 Schema

Group	N*	Primary vaccine regimen				Boosters	
		Month 0	Month 1	Month 3	Month 6	Month 12	Month 18
1	2700	ALVAC-HIV (vCP2438)	ALVAC-HIV (vCP2438)	ALVAC-HIV (vCP2438) + Bivalent Subtype C gp120/MF59	ALVAC-HIV (vCP2438)+ Bivalent Subtype C gp120/MF59	ALVAC-HIV (vCP2438) + Bivalent Subtype C gp120/MF59	ALVAC-HIV (vCP2438) + Bivalent Subtype C gp120/MF59
2	2700	Placebo	Placebo	Placebo + Placebo	Placebo + Placebo	Placebo + Placebo	Placebo + Placebo
Total	5400						

* Due to the randomization scheme, the numbers of vaccine and placebo recipients may differ slightly.

Participants

5400 healthy, HIV-1–uninfected adults in South Africa who are aged 18 to 35 years and who are at risk for HIV infection, with 2700 vaccinees and 2700 placebo recipients. To preserve study power, no more than 35% and no fewer than 30% of participants born male will be enrolled.

Actual enrollment was 5407, 2706 of whom received vaccine and 2701 received placebo. These represented 5404 unique individuals, of whom 2704 received vaccine and 2700 received placebo.

Design

Multicenter, randomized, placebo-controlled, double-blind trial with interim safety and immunogenicity assessments and interim analyses of efficacy

Duration per participant

Minimum 24, maximum 42 months per participant (including 6 months follow-up should a participant be diagnosed with HIV infection at their last scheduled study visit). All HIV-1–uninfected study participants will be followed for at least 24 months following enrollment unless an interim monitoring boundary is reached. If the vaccine efficacy analysis in Stage 1 (ie, months 0-24) supports continuation of the study through Stage 2 (ie, months 24-36), all HIV-1–uninfected study participants will be followed for 36 months of scheduled clinic visits. Participants who become HIV-1–infected during the study will be followed for approximately 6 months after confirmation of diagnosis.

The NIAID DSMB determined on 23 January 2020 that the interim non-efficacy boundary had been reached. Accordingly, vaccinations were stopped on 3 February 2020 and it was determined that participants should be unblinded and that follow up for HIV-uninfected participants should last 12 months following their last vaccination. At that time the last enrolled participant had passed the timepoint for the scheduled Month 6 vaccination; hence, the minimum scheduled study duration for this participant is 18 months. Some participants had already reached the 36-month maximum duration for HIV-1–uninfected participants. As indicated above, participants who become HIV-1 infected during the study will be followed for approximately 6 months after confirmation of diagnosis.

Estimated total study duration

Approximately 62 months. This includes 20 months projected for enrollment, up to 36 months follow-up for the last enrolled HIV-uninfected participant, and 6 months follow-up should the last-enrolled participant be diagnosed with HIV infection at the last [ie, Month 36] scheduled clinic visit.

Following cessation of vaccination and reduction of follow-up duration for HIV-1–uninfected participants, the estimated total study duration is reduced to approximately 44 months.

Study sponsor

DAIDS, NIAID, NIH, DHHS (Bethesda, Maryland, USA)

Study product providers

- ALVAC-HIV (vCP2438): Sanofi-Pasteur (Swiftwater, Pennsylvania, USA)
- Bivalent Subtype C gp120/MF59: GlaxoSmithKline Biologicals, S.A. (Rixensart, Belgium)

Core operations

HVTN Vaccine Leadership Group/Core Operations Center, Fred Hutchinson Cancer Research Center (FHCRC) (Seattle, Washington, USA)

Statistical and data management center (SDMC)

Statistical Center for HIV/AIDS Research and Prevention (SCHARP), FHCRC (Seattle, Washington, USA)

Endpoint assay laboratories

- HIV Sero-Molecular Laboratory–National Institute for Communicable Diseases (HSML-NICD) (Johannesburg, South Africa)

- South Africa Immunology Laboratory–National Institute for Communicable Diseases (SAIL-NICD) (Johannesburg, South Africa)
- Cape Town HVTN Immunology Laboratory (CHIL) (Cape Town, South Africa)
- Duke University Medical Center (Durham, North Carolina, USA)
- FHCRC/University of Washington (Seattle, Washington, USA)
- University of Cape Town (Cape Town, South Africa)
- University of Colorado, Denver (Aurora, Colorado, USA)
- University of Washington Virology Specialist Laboratory (UW-VSL) (Seattle, Washington, USA)

Study sites

HVTN Clinical Research Sites (HVTN CRSs) to be specified in the Site Announcement Memo

Safety monitoring

HVTN 702 PSRT; NIAID DSMB

3.1 Protocol Team

Protocol leadership

Chair
Glenda Gray
Perinatal HIV Research Unit (PHRU), Chris Hani Baragwanath Hospital, Soweto; President, South African Medical Research Council
011-27-11-989 9702
glenda.gray@mrc.ac.za

Cochair
Linda-Gail Bekker
The Desmond Tutu HIV Centre, Cape Town, South Africa
27-21-650-6959
linda-gail.bekker@hiv-research.org.za

Fatima Laher
PHRU, Chris Hani Baragwanath Hospital, Soweto, South Africa
27-11-989-9720
laherf@phru.co.za

Mookho Malahleha
Setshaba Research Centre, Soshanguve, South Africa
27-12-799-2422
MMalahleha@setshaba.org.za

Protocol Team leader
Nicole Grunenberg
HVTN Core, FHCRC
206-667-2043
ngrunenb@fredhutch.org

Statisticians
Yunda Huang
SCHARP, FHCRC
206-667-5780
yunda@scharp.org

Holly Janes
SCHARP, FHCRC
206-667-6353
hjanes@scharp.org

Zoe Moodie
SCHARP, FHCRC
206-667-7077
zoe@scharp.org

Medical officer
Mary Allen
DAIDS, NIAID
240-627-3036
mallen@niaid.nih.gov

Laboratory lead
John Hural
HVTN Laboratory Program, FHCRC
206-667-1683
jhural@fredhutch.com

Other contributors

Core medical monitor
Nicole Grunenberg
HVTN Core, FHCRC

Regional medical liaison
Simba Takuva
HVTN Core, FHCRC

Vaccine developer representatives
Carlos DiazGranados
Sanofi Pasteur

Marguerite Koutsoukos
GSK

Olivier Van Der Meeren
GSK

Laboratory program representative
Lisa Sanders
HVTN Laboratory Center, FHCRC

Regulatory affairs associate
Elizabeth Briesemeister
HVTN Core, FHCRC

Clinic coordinator
Katlego Mapetla
Setshaba CRS

Community Advisory Board (CAB) members
Praise Moyo
Emavundleni CRS

DAIDS protocol pharmacist
Irene Rwakazina
DAIDS, NIAID
301-761-7269

Statistical research associates
Doug Grove
SCHARP, FHCRC

Brittany Sanchez
SCHARP, FHCRC

Social behavioral scientists
Michele Andrasik
HVTN Core, FHCRC

Millicent Atujuna
The Desmond Tutu HIV Centre, University of Cape Town

Clinical safety specialist
Megan Jones
HVTN Core, FHCRC

Clinical trials manager
Shelly Ramirez
HVTN Core, FHCRC

DAIDS Project Officer
Michael Pensiero
DAIDS, NIAID

Protocol development manager
Carter Bentley
HVTN Core, FHCRC

Jim Tabane<br>Setshaba CRS	*Community engagement unit representative*	Kagisho Baepanye<br>HVTN Core, FHCRC
Thembeka Maduna<br>Soweto (PHRU) CRS	*Community educator/recruiter*	Phumla Madi<br>PHRU – Kliptown CRS
	Technical editor	Erik Schwab<br>HVTN Core, FHCRC

4 Background

4.1 Burden of HIV and rationale for HIV vaccine development

Despite remarkable advances in antiretroviral use both to treat HIV and to prevent vertical and horizontal transmission of HIV (4-9), the HIV epidemic grows relentlessly worldwide. Over 2.1 million new infections occur each year, two thirds in women and 240,000 in children (10, 11). In heavily affected countries, HIV infection rates have at best stabilized; in southern Africa the annualized acquisition rates in persons in their first decade of sexual activity average 3-5% (12-14). With approximately 6.8 million people living with HIV as of 2014 (15), South Africa's epidemic remains the largest in the world, and the vast majority of newly acquired infections occur during unprotected heterosexual intercourse. These figures clearly indicate that southern Africa is far from achieving the international health community's stated goal of an "AIDS-free generation" (5).

Treatment scale-up (16), medical male circumcision (17), and rolling out strategies to prevent mother-to-child transmission (4) may help control the HIV epidemic, but biomedical interventions that are not user dependent or time critical (eg, pericoital) and that do not rely on only one gender will be pivotal to curtail the ongoing HIV acquisition seen in young women and their partners. Due to cost, complexity of roll-out, adherence requirements, and the reservation of antiretroviral therapy for treatment, systemic or topical antiretroviral (ARV)–based biomedical interventions (5-8) to prevent sexual acquisition of HIV in southern Africa may have limited impact at a population level, despite reported efficacy. These countries may benefit from alternatives that are sustainable, affordable, and implementable; therefore, an effective HIV vaccine remains an important tool for the long-term control and eventual elimination of HIV transmission and investment in HIV vaccine evaluation remains critical (18).

4.2 Rationale for trial concept

4.2.1 Rationale for the HVTN 702 phase 2b/3 HIV vaccine trial

RV144 was a phase III randomized controlled clinical trial conducted by the US Military HIV Research Program and the Thailand Ministry of Health in approximately 16,000 HIV-1–uninfected adults between the ages of 18-30 years at varying degrees of HIV risk in Thailand (19). The clinical trial evaluated the heterologous prime-boost combination of 2 canarypox primes ALVAC-HIV (vCP1521), expressing clade E *env* and clade B *gag* and *pro*, followed by 2 ALVAC-HIV + AIDSVAX clades B/E gp120 protein boosts. These products were designed to match HIV-1 strains commonly circulating in Thailand at the time. This vaccine regimen demonstrated 31.2% efficacy when compared with placebo (n = 51 vs. n = 74, respectively; p = 0.04) at 3.5 years (19). Although evaluation of vaccine efficacy at 12 months post vaccination was not included in the pre-specified analysis, substantially greater reduction in acquisition was

observed one year postvaccination (estimated 60.5%, 95% confidence interval [CI] 22%-80%), with the vaccine effect waning over time (Figure 4-1) to 31% cumulative through 3.5 years (20).

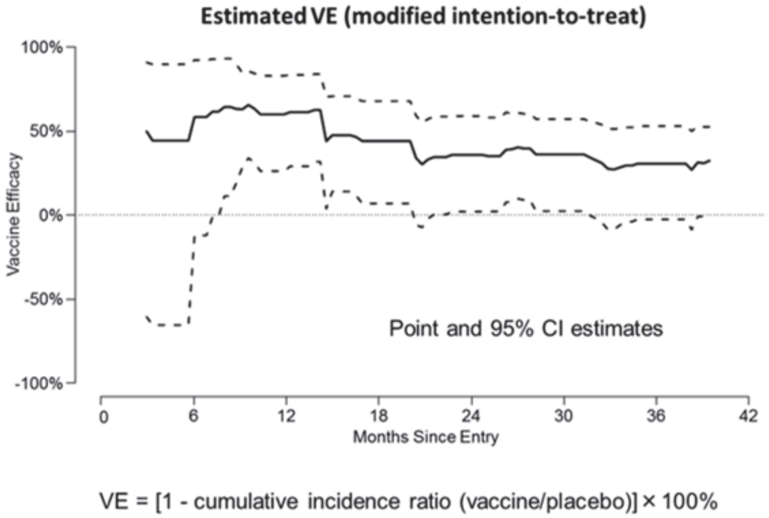

Figure 4-1 Observed vaccine efficacy (cumulative over time) in the RV144 trial (figure produced by P. Gilbert with data published by Robb et al [20])

Following the announcement of these promising efficacy results (19) and the subsequent identification of immunological correlates of risk in that trial (21) (Section 4.2.2 below), an effort was undertaken by a public-private partnership (the Pox-Protein Public Private Partnership [P5]) to adapt the RV144 ALVAC-HIV/gp120 vaccine design to more closely match the subtype C HIV strains predominant in southern Africa (22-33), the region most in need of an effective prophylactic HIV vaccine (34-38). The resulting vaccine regimen was designed to strike a balance between maintaining a close link to the RV144 vaccine regimen and the need to target the clade C HIV-1 virus while, at the same time, potentially improving the magnitude and durability of immune responses in ways that may translate into improvements in vaccine efficacy (Section 4.2.3).

The trigger to initiate enrollment of HVTN 702 will be based on the safety and immunogenicity data from HVTN 100, a phase 1-2 trial of the same vaccine regimen. HVTN 100 data demonstrating that the vaccine regimen with 2 primes and 2 boosts, regardless of the 3rd boost, is safe and produces key immune responses comparable or superior to those induced by the RV144 vaccine regimen will be the impetus for evaluating the preventive efficacy of the regimen.

HVTN 702 is a pivotal phase 2b/3 trial designed so that, if the vaccine regimen proves sufficiently efficacious, the data will support an application for marketing authorization in the Republic of South Africa (RSA).

4.2.2 Correlates of risk (CoR) in RV144

To better understand how the RV144 vaccine regimen reduced the risk of HIV infection, a large consortium of independent laboratories worked together systematically to ensure that maximal information could be derived from samples obtained from RV144 vaccinees who became infected compared with vaccinees who remained HIV uninfected at the end of the trial. A case control study was performed on 41 infected vaccine recipients, 205 uninfected vaccine recipients (5:1), and 40 placebo recipients (20 infected and 20 uninfected) within the RV144 clinical trial to identify CoR (21). Among the 6 primary immunological variables selected for the correlates analysis (5 different antibody [Ab] responses and CD4+ T cell cytokine production) that were measured at the visit 2 weeks after the final vaccination (ie, at or near peak immunogenicity), 2 immune CoR of HIV acquisition were identified among vaccine recipients in the RV144 case control study. The first was the presence of immunoglobulin G (IgG) Ab that bound to a scaffolded gp70 V1V2 recombinant protein; this variable correlated inversely with infection rate (ie, higher IgG titers to V1V2→lower infection rate), a finding subsequently extended to scaffolded gp70 V1V2 proteins from multiple HIV-1 subtypes, including clade C (39). The second was plasma Env-specific binding immunoglobulin A (IgA), which correlated directly with infection rate (ie, higher IgA Ab to Env→higher infection rate). The other 4 primary variables correlated inversely with infection rate only when the level of IgA binding was low. Notably, neither low levels of the V1V2 Ab nor high levels of Env-specific IgA was associated with a higher rate of infection than observed in the placebo group (21).

Recently, several studies have further enhanced our understanding of the efficacy seen in RV144. Rolland and colleagues demonstrated a sieve effect in the vaccine recipients, showing that the vaccine induced better protection against viruses that matched the vaccine sequence at position 169 in the V2 loop of Env (40). These data further substantiate the importance of antibodies directed against this region in protecting against infection (21). Yates and colleagues noted that Env V1V2-specific IgG3 was the immunoglobulin subclass showing the strongest negative correlation with risk of HIV acquisition in RV144 (41). Chung and colleagues demonstrated that the IgG3 subclass engaged Fc-mediated effector responses more effectively than other IgG subclasses, thereby providing a possible mechanism explaining the association of Env V1V2 IgG3 with a lower rate of HIV acquisition (42). In addition, while the original search for correlates of risk failed to demonstrate an association between CD4+ T-cell responses and infection risk, Lin et al (43) recently used a computational framework for unbiased combinatorial polyfunctionality analysis of antigen-specific T-cell subsets (COMPASS) to identify CD4+ T-cell subsets in RV144 vaccinees that correlated with reduced risk of infection. One subset of polyfunctional CD4+ T cells (ie, those expressing TNF-α, IFN-γ, IL-4, IL-2, and CD40L) was as significantly associated with reduced infection risk as IgG responses to the V1V2 region of Env. Notably, the two subsets identified by the COMPASS algorithm as significantly correlated with reduced infection risk both included T cells expressing IL-4 and CD40L, cytokine and functional markers important for CD4+ T cell and B cell interactions, leading the authors to suggest that the specific

polyfunctional T-cell subsets they identified may contribute T-cell help for production of the antibody detected as a correlate in the primary analysis, though this specific functional association has yet to be demonstrated. In sum, these CoR studies point to the importance of Ab responses, directed against a specific region of Env, in mediating the differing rates of HIV acquisition observed in RV144 and also suggest an important role for CD4+ T cell help in generating those Ab responses. They lay the groundwork for immunogenicity analyses planned for HVTN 100 and for future HIV vaccine clinical trials.

4.2.3 Rationale for the proposed vaccine regimen for South Africa

This clinical trial will be conducted in South Africa, where clade C is the predominant circulating HIV subtype (33), and will evaluate the efficacy, safety, and immunogenicity of the regionally adapted ALVAC/protein vaccine regimen. The regimen will comprise 2 administrations of ALVAC-HIV (vCP2438) containing clade C ZM96 gp120 *env* along with clade B (LAI) *gag*, *pro* and *gp41 env TM* at Months 0 and 1, followed by 3 administrations of the same ALVAC-HIV along with Bivalent Subtype C gp120/MF59 (comprising recombinant TV1.C and 1086.C gp120 Env proteins mixed with MF59 adjuvant), at Months 3, 6, and 12.

The vaccine regimen in this trial maintains a close link to the RV144 vaccine regimen while targeting the clade C HIV strains circulating in southern Africa, the region of the world where deployment of the vaccine is envisioned. In addition, the vaccine design and vaccination schedule have been altered in ways that aim at improving the magnitude and duration of vaccine-elicited immune responses beyond those observed in RV144. These changes have been made under the scientific assumption that improvements or extensions of immune responses will translate into improvements in vaccine efficacy.

The *gag*, *pro*, and *gp41 env* components of ALVAC-HIV (vCP2438) are the same as in ALVAC-HIV (vCP1521), the ALVAC-HIV used in the RV144 trial. Adaptations of the RV144 vaccine regimen to South Africa include use of the 96ZM651 *gp120 env* insert (subtype C) rather than the TH023 *gp120 env* insert (subtype E) used in RV144, and inclusion of 2 subtype C gp120 Env proteins (TV1.C and 1086.C) in boost vaccinations (rather than the subtype B and E proteins used in RV144).

The TV1.C and 1086.C gp120 Env proteins comprising Bivalent Subtype C gp120 were selected from among a long list of candidate clade C gp120 proteins according to a predefined algorithm incorporating, among other factors, genetic relatedness to regional HIV strains, CCR5 binding, capacity in animal studies to elicit key epitope-specific Ab, percent monomer expression (associated with receptor and co-receptor binding and induction of neutralizing antibodies [nAb]), expression/secretion levels in stable cell lines, stability, and immunogenicity in animal models (including evidence of recognition of key epitopes identified in the RV144 correlates studies) (44, 45). Use of gp120 monomers rather than trimers was prioritized to support comparisons with the RV144 results (46).

MF59 was selected as the protein adjuvant for multiple reasons, including its established safety record and previous experience with this adjuvant in similar prime-boost HIV vaccine regimens (47, 48). Based on related human experience, it is expected that MF59 will provide significant antigen dose sparing compared to proteins adjuvanted with alum, while potentially improving the magnitude and subsequent durability of the immune responses elicited by the RV144 regimen. In addition, MF59 has the capacity to provide a more balanced Th1/Th2 response than alum (49). MF59 was originally approved in Europe to enhance the immunogenicity of seasonal influenza vaccine among the elderly (50). MF59-adjuvanted influenza vaccine is now licensed in more than 20 countries with more than 100 million doses having been distributed. Recently, influenza vaccine formulated with MF59 has been shown to be safe in young children and to significantly enhance immune responses and efficacy in this group (51). MF59 is also used in a prepandemic H5N1 influenza vaccine (Aflunov) licensed in the European Union (EU) for use in adults, and in two pandemic H1N1 influenza vaccines (Focetria and Celtura), licensed in EU and other countries for use in adults and children (52).

If the immune responses elicited by the proposed vaccine regimen are similar to those associated with the level of protection seen in RV144 at 6.5 months after the first vaccination, close to the peak of observed vaccine efficacy, then it is believed that there will be sufficient feasibility to demonstrate that the vaccine efficacy will be close to 50% at 24 months after first vaccination. Given the observed vaccine efficacy in RV144 (Figure 4-1), Figure 4-2, Figure 4-3, and Figure 4-4 show graphically how the additional booster vaccination (Section 4.5.3) and potentially enhanced immune responses elicited by the revised vaccine regimen may translate into improved vaccine efficacy and durability.

Figure 4-2 presents the observed vaccine efficacy for study RV144. The vaccine efficacy observed 12 months after the last vaccination in RV144 (18 months after the first vaccination) was 44%.

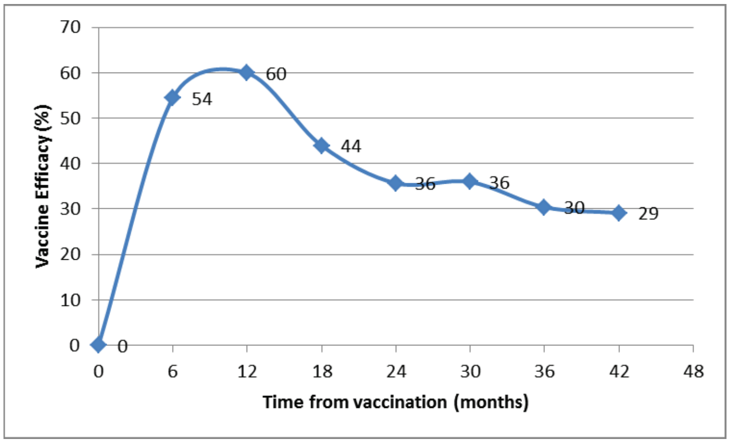

Figure 4-2 Observed vaccine efficacy over time in the RV144 trial

Figure 4-3 plots the same data from RV144 as Figure 4-2 but assumes that peak 60% efficacy is maintained for 6 more months due to the planned booster

vaccination at month 12. This assumes that the booster vaccination induces immune responses associated with the same level of protection as the month 6 vaccination. The vaccine efficacy decay curves are identical in Figure 4-2 and Figure 4-3, but the decay starts 6 months later in Figure 4-3.

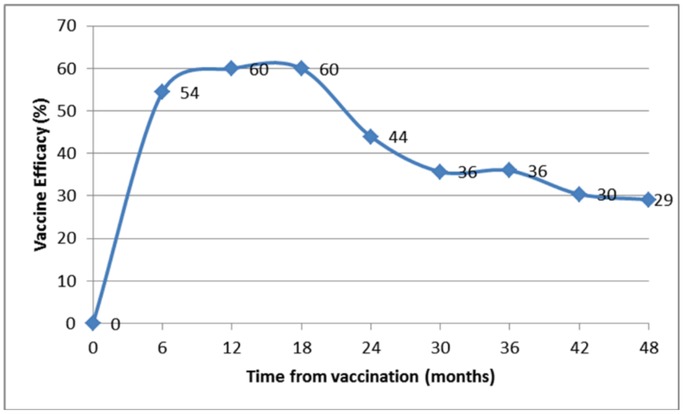

Figure 4-3 Expected vaccine efficacy for a regimen with efficacy comparable to that observed in RV144 but with a booster vaccination added at month 12

As seen in Figure 4-3, if this vaccine regimen is associated with the same peak level of protection as was observed in RV144 and the booster vaccination can maintain the peak level of protection for 6 months more, the vaccine efficacy to month 24 is projected to be 44%, which is higher than that observed for RV144.

Study RV305, a follow-up study to RV144 also conducted by the USMHRP, demonstrated enhanced immune responses with an additional booster vaccination of the ALVAC/protein regimen used in the RV144 study. Geometric mean (GM) titers of IgG responses to gD+ gp120 A244 antigen and to gp70 V1V2CaseA2 and gp70 V1V2 92TH023 scaffolds 2 weeks after the booster vaccination were several-fold higher than the immune responses observed at the month 6.5 timepoint (53). If this improvement in immune responses is associated with improved protection and can be replicated after the booster vaccination given at month 12, then the vaccine efficacy observed between months 12 and 18 may be higher than the vaccine efficacy observed between months 6 and 12 in RV144.

Figure 4-4 presents a graphical representation of this scenario, and assumes that the improved immune responses resulting from the booster vaccination are capable of improving protection by 10% between months 12 and 18.

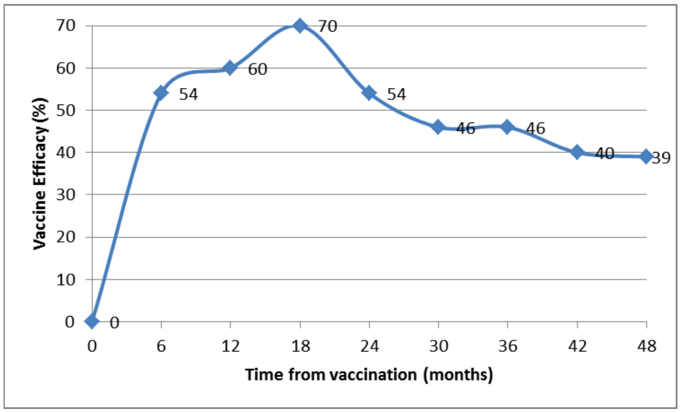

Figure 4-4 Expected vaccine efficacy for a regimen with protective efficacy comparable to that observed in RV144 over the first 12 months, and 10% improved protection from a booster vaccination at month 12.

In Figure 4-4, the vaccine efficacy decay curve is identical as the one observed in RV144, but it starts 6 months later and at a higher level. For this scenario the vaccine efficacy is projected to be 54% at 24 months and 46% at 36 months, which is substantially higher than what was observed in RV144.

4.2.4 Rationale for booster vaccination at Month 18

The immune responses induced by the clade C ALVAC/Bivalent gp120 vaccine regimen in HVTN 100 met predetermined criteria for going forward with HVTN 702, including IgG binding antibody response rates and magnitudes to vaccine-matched Env antigens, CD4+ T-cell response rate to the ALVAC ZM96 Env insert, and V1V2 response rate. The predetermined criteria are described in Table 4-16 and the results are summarized in Section 4.9.6.4 (Figure 4-15, Table 4-17, and Table 4-18). In particular, the V1V2 IgG binding antibody response rate criterion was met for the 1086.C V1V2 protein antigen, while the response rate for any clade C V1V2 antigen was 84%, well above the 63% response rate that served as the basis for the efficacy projections in Section 4.2.3.

Recently, HVTN 100 immunogenicity data from Month 12 (6 months post the 4th vaccination), Month 12.5 (2 weeks post the Month 12 booster vaccination), and Month 18 (6 months post the 5th vaccination) have become available. This data shows that overall, IgG responses decrease by 6 months following the 4th vaccination, rebound following the 5th vaccination to levels similar to post 4th vaccination peaks, and by 6 months post 5th vaccination, decline again but to levels higher than to those at 6 months post 4th vaccination. The IgG responses to V1V2 antigens are substantially lower at the Month 12 and 18 durability timepoints than are the responses to gp120 antigens (Figure 4-5 and Figure 4-6).

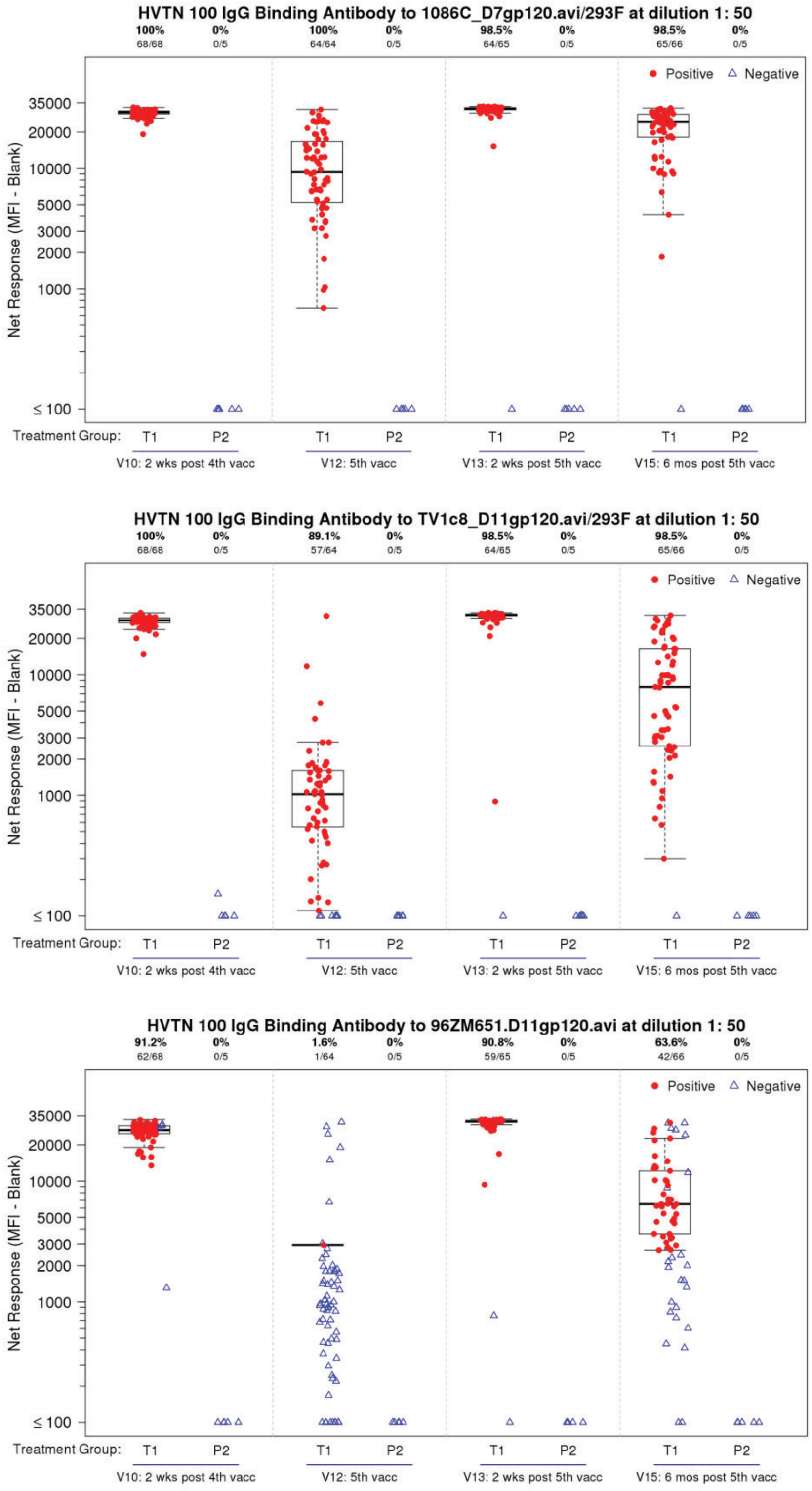

Figure 4-5 IgG antibody responses to vaccine-matched gp120 antigens at visit 10 (month 6.5, 2 weeks post 4th vaccination), at visit 12 (month 12, 6 months post 4th vaccination), at visit 13 (month 12.5, 2 weeks post 5th vaccination), and at visit 15 (month 18, 6 months post 5th vaccination). MFI – Blank = negative control adjusted mean fluorescent intensity.

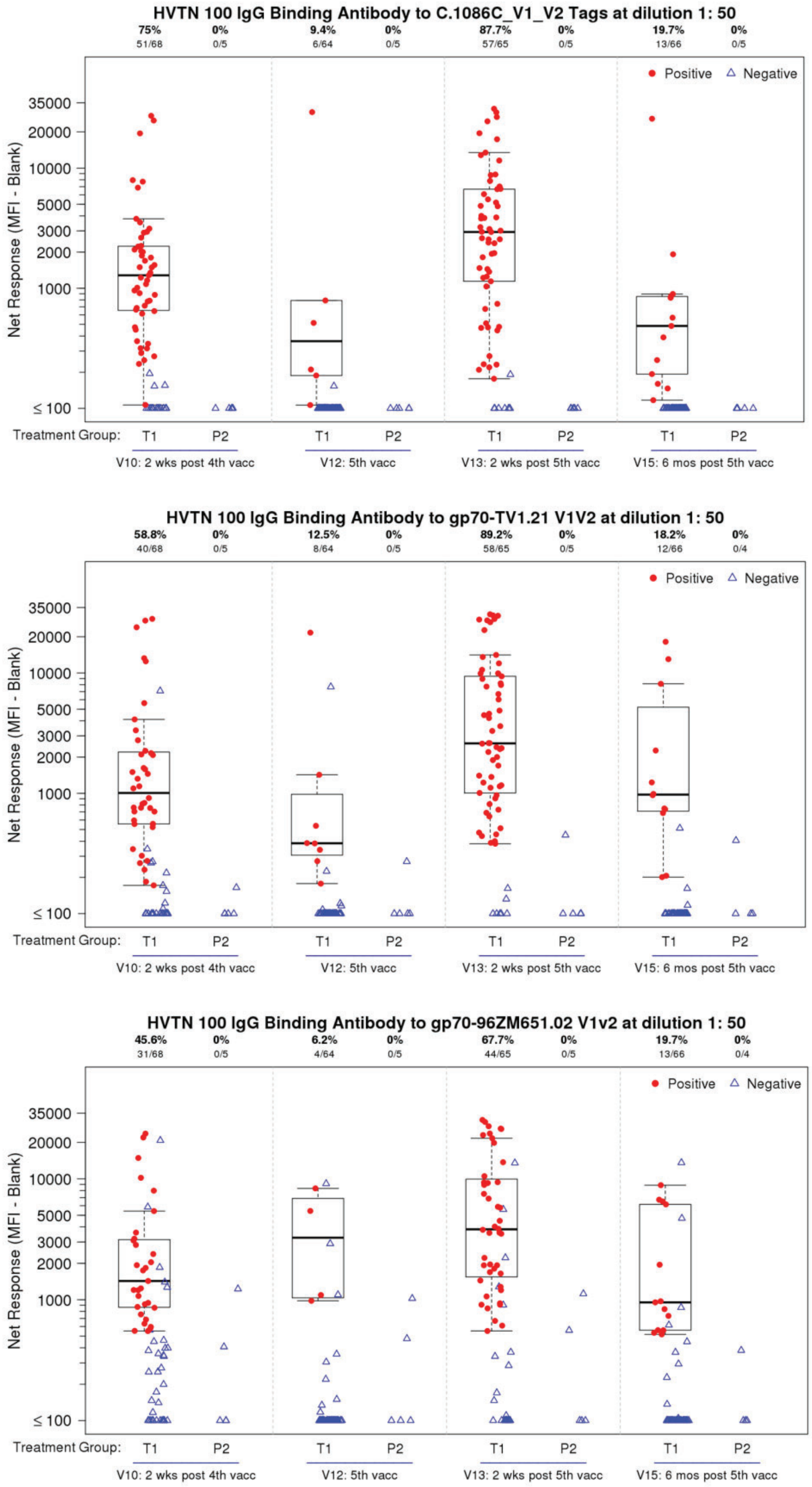

Figure 4-6 IgG antibody responses to vaccine-matched gp70 V1V2 antigens at visit 10 (month 6.5, 2 weeks post 4th vaccination), at visit 12 (month 12, 6 months post 4th vaccination), at visit 13 (month 12.5, 2 weeks post 5th vaccination), and at visit 15 (month 18, 6 months post 5th vaccination). MFI – Blank = negative control adjusted mean fluorescent intensity.

The response rate to the gp70 CaseA2 V1V2 antigen at Month 12 and Month 18 is also low (12.5% and 15%, respectively, Figure 4-7), whereas binding antibody response magnitude to this antigen was the primary correlate of risk identified in the RV144 trial.

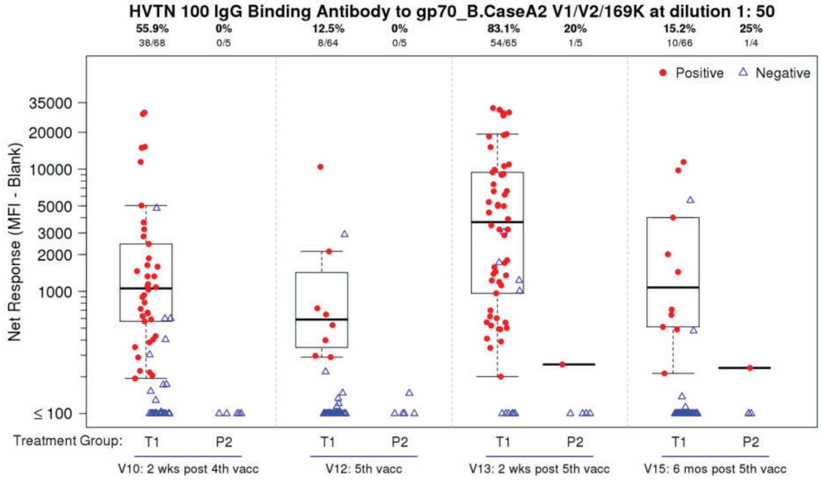

Figure 4-7 IgG antibody responses to gp70 CaseA2 V1V2 antigen at visit 10 (month 6.5, 2 weeks post 4th vaccination), at visit 12 (month 12, 6 months post 4th vaccination), at visit 13 (month 12.5, 2 weeks post 5th vaccination), and at visit 15 (month 18, 6 months post 5th vaccination). MFI – Blank = negative control adjusted mean fluorescent intensity.

In addition, the breadth of IgG responses was assessed against a panel of 16 clade C V1V2 antigens and the area under the magnitude-breadth curve (AUC) was calculated (54). The overall AUC analyses showed that following the 5th vaccination, responses at peak (2 weeks) and durability (6 months) timepoints were greater than compared to those timepoints following the 4th vaccination (Figure 4-8, panel A and B).

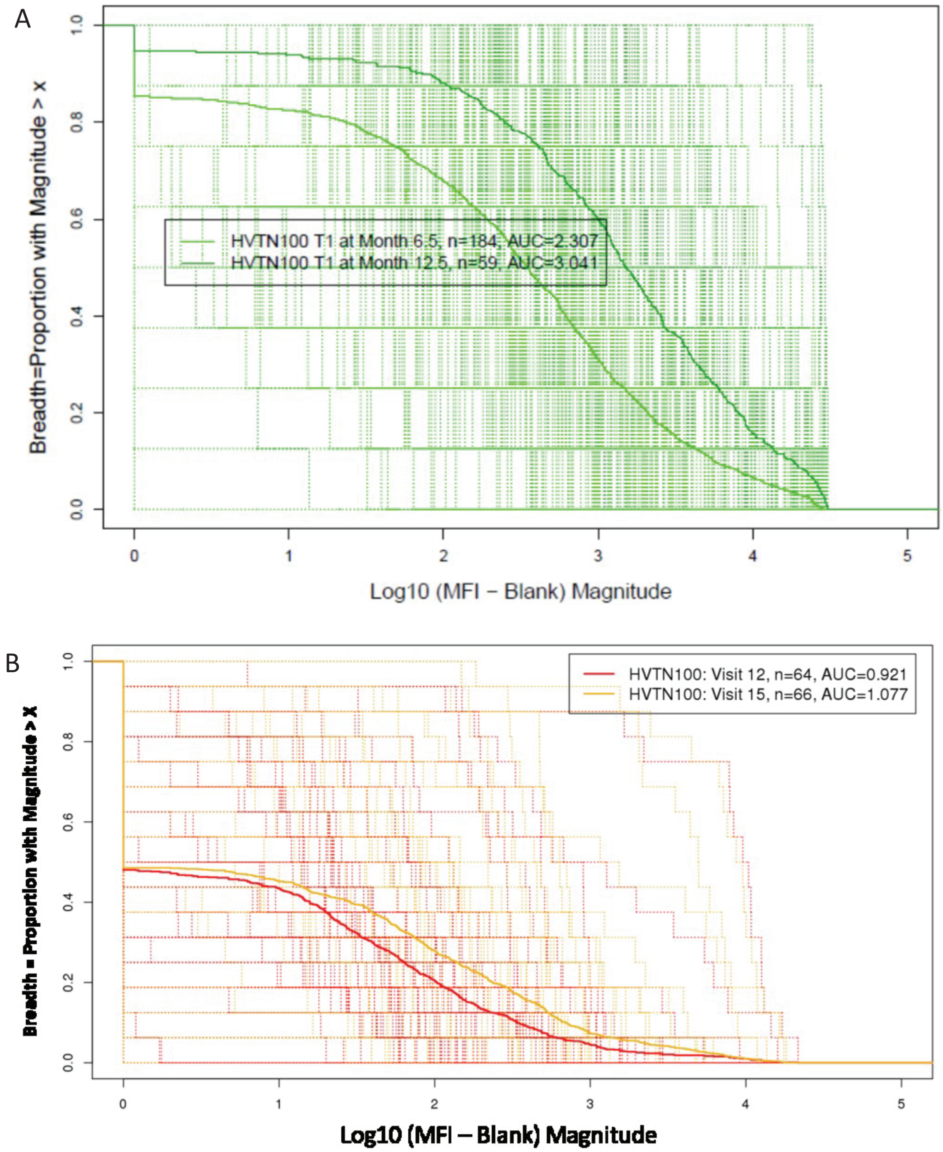

Figure 4-8 V1V2 Magnitude- breadth curves of IgG binding antibody responses to 16 different clade C V1V2 antigens among per-protocol HVTN 100 vaccine recipients at visit 10 (month 6.5, 2 weeks post 4th vaccination) versus visit 13 (month 12.5, 2 weeks post 5th vaccination) in panel A and at visit 12 (month 12, 6 months post 4th vaccination) versus visit 15 (6 months post 5th vaccination) in panel B. Solid curves are average breadth across individuals with breadth defined by the proportion of antigens in the panel with log10 (MFI – blank) greater than the threshold on the x-axis.

The Env-specific CD4$^+$ T-cell response rate declined 6 months after the 4th vaccination but was restored after boosting at month 12. Response rates declined less within 6 months following the 5th vaccination than within 6 months following the 4th vaccination (Figure 4-9).

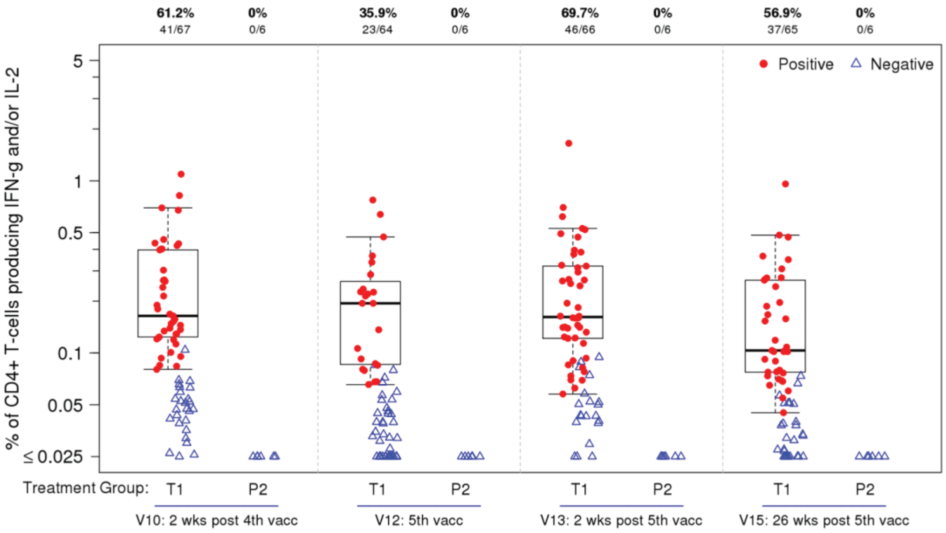

Figure 4-9 CD4$^+$ T cells expressing IFN-γ and/or IL-2 in response to stimulation with vaccine-matched Env antigens at visit 10 (month 6.5, 2 weeks post 4th vaccination), at visit 12 (month 12, 6 months post 4th vaccination), at visit 13 (month 12.5, 2 weeks post 5th vaccination), and at visit 15 (month 18, 6 months post 5th vaccination)

The boost given at month 12 was intended to enhance the durability of the immune responses. The data from the month 12 boost shows a steep decline in IgG binding antibody responses to V1V2 antigens by 6 months following each vaccination, yet also overall enhancement of these key immune responses at peak and durability timepoints following the last boost. Therefore, HVTN 702 is amended to add an additional booster vaccination at month 18 in an effort to increase V1V2 responses and correspondingly to optimize vaccine efficacy by month 24. Based on models projecting vaccine-matched and heterologous clade C V1V2 antibody response rates and magnitudes, working with observed HVTN 100 data at Months 6.5, 12, 12.5, and 18, the addition of a month 18 boost in HVTN 702 is expected to generate 0.26 - 0.41 higher expected log MFI V1V2 responses between Month 6.5-24, as compared to the regimen with no boost at Month 18 (Figure 4-10). While the role of V1V2 binding antibody response in predicting VE in HVTN 702 is not yet determined, the addition of a Month 18 boost will likely improve the opportunity for this regimen to demonstrate, for the first time, the capacity of an HIV vaccine to prevent HIV infection in South Africans.

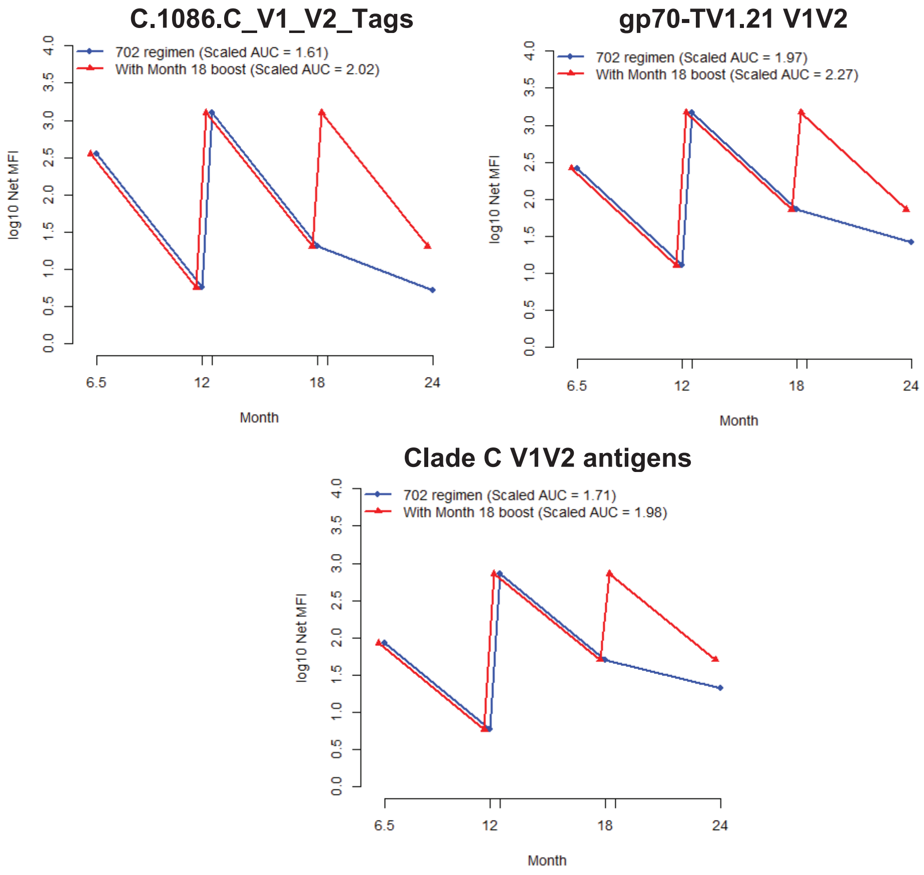

Figure 4-10 Predicted average immune response profiles for C.1086C_V1_V2 Tags, gp70-TV1.21 V1V2, and clade C V1V2 antigens. The area under the average immune response profile (AUC) is calculated using the trapezoidal rule, and divided by 17.5 (month 24 minus month 6.5); the resultant scaled AUC approximates the expected immune response at time of HIV exposure.

4.3 ALVAC-HIV (vCP2438)

ALVAC-HIV (vCP2438) is a preparation of live, attenuated recombinant canarypox-derived virus expressing products from the HIV-1 *env gp120* (clade C), *env gp41 TM* (clade B), *gag* (clade B), and *protease* (clade B) coding sequences and cultured in primary chicken embryo fibroblasts (CEFs).

4.3.1 Constructs

The original strain of canarypox virus (Rentschler strain) was attenuated by serial passages on CEFs. The attenuated virus was plaque isolated and designated as ALVAC. Details of the manufacturing process are provided in the Investigator's Brochure (IB).

The construct is summarized in Table 4-1. The inserted HIV-1 gene sequences are:

- The region of the *env* gene encoding the extracellular Env gp120 moiety of the 96ZM651 strain of HIV-1 linked to the sequence encoding the HIV-1 TM anchor sequence of gp41 (28 amino acids) from HIV-1 strain LAI. The *env* gene sequence is under the control of the vaccinia virus H6 promoter.

- The *gag* gene encoding the entire Gag protein and a portion of the *pol* sequences of the LAI strain of HIV-1 sufficient to encode the protease function. The *gag/protease* gene sequences are under the control of the same vaccinia virus I3L promoter.

Table 4-1 ALVAC-HIV (vCP2438) construct summary table

Inserted gene	Strain	Promoter	Insertion Locus
env (gp120 + gp41 TM)	96ZM651 (gp120)<br>LAI (gp41 TM)	H6 (vaccinia)	C6
gag + *pro*	LAI	I3L (vaccinia)	C6

4.3.2 Formulation characteristics

ALVAC-HIV (vCP2438) is formulated as a lyophilized vaccine for injection and is reconstituted with 1.0 mL of sterile sodium chloride solution (NaCl 0.4%) for injection as a single dose. The composition of one dose of ALVAC-HIV (vCP2438) is provided in Table 4-2.

Table 4-2 Composition of ALVAC-HIV (vCP2438)

Ingredient	Amount in one dose	Function
ALVAC-HIV (vCP2438)	$\geq 1 \times 10^6$ $CCID_{50}$ and $< 1 \times 10^8$ $CCID_{50}$	Immunogen
Tris-HCl	0.3 mg	Buffer
Lactose-monohydrate	26.325 mg	Component of lactoglutamate stabilizer
L-Glutamic acid	0.278 mg	Component of lactoglutamate stabilizer
$NaH_2PO_4.2H_2O$	0.15 mg	Component of lactoglutamate stabilizer
K_2HPO_4	0.55 mg	Component of lactoglutamate stabilizer
KOH	0.1 mg	Component of lactoglutamate stabilizer
Sucrose	50 mg	Component of freeze-drying stabilizer
Sodium glutamate monohydrate	5.5325 mg	Component of freeze-drying stabilizer
HCl	1.8 mg	Component of freeze-drying stabilizer
Non-essential amino acids	1.628 mg	Component of freeze-drying stabilizer
Essential amino acids	4.46 mg	Component of freeze-drying stabilizer

4.3.3 Manufacturing

ALVAC-HIV (vCP2438) Bulk Drug Substance is manufactured by IDT Biologika GmbH, Am Pharmapark, Dessau-Rosslau, Germany, under contract to

Sanofi Pasteur. ALVAC-HIV (vCP2438) Drug Product is manufactured at the Sanofi Pasteur SA, facility located in Marcy l'Etoile, France. The diluent used for reconstitution is manufactured at the Sanofi Pasteur Inc. facility located in Swiftwater, Pennsylvania (USA).

ALVAC-HIV (vCP2438) is produced by inoculating the viral seed lot into cultured primary CEFs derived from eggs produced by specific pathogen-free (SPF) flocks.

The manufacturing process for ALVAC-HIV (vCP2438) is similar to that for ALVAC-HIV (vCP1521) used in RV144.

4.4 Bivalent Subtype C gp120/MF59

4.4.1 Constructs

Bivalent Subtype C gp120/MF59, manufactured by Rentschler Biotechnologie (Laupheim, Germany), consists of two subtype C recombinant monomeric proteins, TV1.C gp120 and 1086.C gp120. These recombinant gp120s represent the receptor binding domain of the HIV envelope glycoprotein. Each gp120 is modified from its wild type full-length form (gp160) by replacement of the native signal sequence and deletion of the entire gp41 C-terminal portion of the glycoprotein containing the TM and cytoplasmic domains. The combination of the 2 subtype C gp120 proteins and the MF59 adjuvant is referred to as Bivalent Subtype C gp120/MF59.

4.4.2 Manufacturing and formulation

Each protein is expressed in Chinese hamster ovary (CHO) cells under conditions favorable for secretion of monomeric protein. Following fermentation, each protein is extensively purified from culture supernatants, including further enrichment for monomer.

Following clone selection, a fed batch cell culture at 250L scale is employed for cell propagation. Once the cells reach optimum cell density, the culture is harvested and purified using standard methods. The conditioned media is concentrated by ultrafiltration. The manufacturing process utilizes a weak cation exchange chromatography step, CM-Fractogel, which provides purification as well as viral reduction. Concentration (by ultrafiltration) is then used, followed by exchange into the formulation buffer.

After these process steps, both subtype C gp120 protein processes include viral reduction filtration (nanofiltration) followed by 0.22 μm filtration and bulk fill. Following formulation and vialing, both TV1.C and 1086.C drug substances are stored frozen at not more than -61°C. For both drug substances, the formulation contains 0.8 mg/mL Env antigen, sodium citrate, and sodium chloride, pH 6.5 for TV1.C and pH 7.0 for 1086.C.

The composition per dose of each subtype C gp120 vaccine protein is provided in Table 4-3.

Table 4-3 Qualitative composition of Subtype C gp120 drug substances vials

Ingredient	Function
gp120 protein	active
Sodium Citrate, Dihydrate	buffer
Citric Acid, Monohydrate	buffer
Sodium Chloride	tonicity modifying agent
Water for injections	solvent

Additional information is provided in the Bivalent Subtype C gp120/MF59 IB.

4.4.3 MF59 adjuvant

The Novartis MF59 adjuvant is an oil-in-water emulsion with a squalene internal oil phase and a citrate buffer external aqueous phase. Two non-ionic surfactants, sorbitan trioleate and polysorbate 80, serve to stabilize the emulsion. The bulk formula is shown in Table 4-4.

Table 4-4 Composition of MF59 per liter

Name of Ingredients	Quantity per Litre*	Function	Reference to Standards
Squalene	39.0 g	oil phase	In-house specification
Polysorbate 80	4.7 g	surfactant	USP/NF
Sorbitan Trioleate	4.7 g	surfactant	USP/NF
Sodium Citrate, dihydrate	2.65 g	buffer	USP/NF
Citric Acid, monohydrate	0.17 g	buffer	USP/NF
Water for Injection	q.s. 1 L	aqueous phase	Ph.Eur. and USP/NF
Nitrogen	overlay	inert gas	USP/NF

*An overage of up to 10% is included to compensate for manufacturing losses.

The full dose of MF59 utilized in the marketed Fluad vaccine (containing 9.75 mg of squalene) will be utilized for formulation with subtype C recombinant envelope gp120 proteins (described above).

The MF59 (full name: MF59C.1) manufacturing process consists of five manufacturing steps: raw materials dispensing and blending, premixing, emulsification, sizing filtration, and filling.

The MF59 bulk resulting at the end of the process is filled into the vials with an overlay of nitrogen and stored protected from light at 2-8° C.

Additional information is provided in the Bivalent Subtype C gp120/MF59 IB.

4.4.4 Bivalent Subtype C gp120/MF59 for injection

A final dose of 100mcg of each recombinant Env protein will be mixed with MF59 adjuvant. The composition of one dose of the resulting vaccine is shown in Table 4-5.

Table 4-5 Composition of 0.5 mL dose of Bivalent Subtype C gp120/MF59 for injection

Ingredient	Amount in one dose	Function
**Drug Substances**		
TV1.C gp120 protein<br>1086.C gp120 protein	100 mcg<br>100 mcg	active<br>active
**Adjuvant (MF59)**		
Squalene	9.75 mg	oil phase
Polysorbate 80	1.175 mg	surfactant
Sorbitan Trioleate	1.175 mg	surfactant
**Excipients**		
Sodium Citrate, Dihydrate<br>Citric Acid, Monohydrate<br>Sodium Chloride<br>Water for injections	1.39 mg<br>0.051 mg<br>4.38 mg<br>qs to 0.5 mL	buffer<br>buffer<br>tonicity modifying agent<br>solvent

4.5 Trial design rationale

Based on data supporting the hypothesis (tested in the HVTN 100 phase 1-2 trial) that 2 doses of ALVAC-HIV (vCP2438) followed by 2 doses of ALVAC-HIV (vCP2438) + Bivalent Subtype C gp120/MF59 would generate in a South African study population key immune responses at least comparable to those induced by the RV144 vaccine regimen, the current trial will assess whether this vaccine regimen can demonstrate efficacy in prevention of HIV-1 infection in HIV-seronegative adults in South Africa. The study is designed with sufficient power to provide conclusive evidence of vaccine efficacy to be used to support a potential Marketing Authorisation Application in the Republic of South Africa.

4.5.1 Dose (amount and number)

ALVAC-HIV (vCP2438): Viral titer $\geq 1 \times 10^6$ $CCID_{50}$ and $< 1 \times 10^8$ $CCID_{50}$ (nominal dose of 10^7 $CCID_{50}$) lyophilized vaccine to be reconstituted for IM injection.

This study will utilize doses of ALVAC-HIV that are within the same range as was used for the RV144 study and for HVTN 097 (Section 4.9.5). The ALVAC-HIV (vCP1521) dose targeted for study RV144 was $> 10^6$ $CCID_{50}$. The actual ALVAC-HIV titers from the 12 vaccine lots used in the RV144 study ranged from $10^{7.06}$ $CCID_{50}$ to $10^{7.41}$ $CCID_{50}$. The measured titer of the vaccine lot used in HVTN 097 was $10^{7.72}$ $CCID_{50}$ (ie, 2-4.5–fold higher than the dose used in RV144);

however, a dose effect on immunogenicity was not observed in a dose ranging study of an ALVAC HIV vaccine (HVTN 039).

Titers of the ALVAC-HIV (vCP205) construct used in studies ranged from $10^{5.6}$ $CCID_{50}$ to $10^{6.85}$ $CCID_{50}$. A dose response analysis was conducted with samples collected on Days 98 and 182 in the AIDS Vaccine Evaluation Group (AVEG) 022, 022A, 027, 032, 033, 034 and 034A studies. In summary, these data indicate that while there is not a positive dose-response relationship between ALVAC-HIV and cytotoxic T lymphocyte (CTL) responses, use of the lower titer is not optimal for induction of nAb responses. Therefore, many clinical studies in humans have targeted an ALVAC dose > 10^6 $CCID_{50}$.

HVTN 039 is the only study that has compared the safety and immunogenicity of ALVAC-HIV (vCP1452) given at the standard dose ($10^{7.25}$ $CCID_{50}$) to a dose 5.6 times higher (10^8 $CCID_{50}$), and placebo. The high-dose ALVAC-HIV (vCP1452) resulted in unacceptable levels of reactogenicity, without evidence of enhanced immunogenicity. Although extrapolation of these findings to other ALVAC-HIV vaccines requires caution, the study suggested that an ALVAC-HIV dose < 10^8 $CCID_{50}$ is desirable.

The desired improvements in overall and protective immune responses will rely on the use of a more potent protein adjuvant and adjustment of the vaccination schedule.

For the Bivalent Subtype C gp120/MF59 vaccine component, 100 mcg each of the two gp120 subtype C proteins (TV1.C and 1086.C) will be admixed with the oil-in-water emulsion MF59 (9.75 mg squalene) by the Pharmacist at each CRS prior to IM administration. The 200 mcg total dose Bivalent Subtype C gp120 proteins was selected based on previous clinical experience. Limited dose range studies performed with Novartis (formerly Chiron) subtype B SF2 gp120 and subtype E gp120 protein candidates indicated that 50 mcg and 100 mcg, totalling 150 mcg, doses with MF59 adjuvant were immunogenic and well tolerated (55).

4.5.2 Assumptions regarding HIV-1 incidence

HIV-1 incidence estimates from previous clinical trials among at-risk study populations in South Africa include the following:

- The Orange Farm male circumcision study (2002-2005), in which HIV-1 incidence among the untreated male population, 18-24 years of age was estimated at 2.1 per 100 person-years (56).

- The HVTN 503 (Phambili) HIV vaccine study (2007), in which HIV-1 incidence among placebo recipients, 18-35 years of age was estimated at 3.7 per 100 person-years (5.86 for females and 1.93 for males) (57).

- The CAPRISA 004 vaginal microbicide study (2007-2010), in which HIV-1 incidence was estimated at 9.1-11.2 per 100 person-years among female placebo recipients, 18-40 years of age (58).

- The FACTS 001 vaginal microbicide study (2011-2014), in which HIV-1 incidence was estimated at 4.0 per 100 person-years in women age 18-30 (59).

Additional estimates of HIV-1 incidence among at-risk study populations in Southern Africa come from:

- The VOICE study of vaginal microbicide and systemic prophylactic antiretroviral drugs (2009-2013), in which overall HIV-1 incidence was 5.7% and incidence in the 3 placebo arms of the trial varied from 4.2 to 6.8 per 100 person-years, while the annual incidence in single women in South Africa aged 25 or younger was up to 10% (60).

- The Fem-PrEP study of oral Truvada (2009-2012), in which HIV-1 incidence was estimated at 5.0 per 100 person-years overall and ranged from 3.4–6 per 100 person years in South African female placebo recipients aged 18-35 (61).

- HPTN 043 (2009-2011), a study of community mobilization, mobile testing, same-day results, and post-test support in sub-Saharan Africa and Thailand, in which HIV-1 incidence estimates were 1.2 and 2.4 per 100 person-years for African male and female participants, respectively (Thai sites were excluded from these incidence estimates) (62).

- The MIRA study of diaphragm, lubricant gel, and condoms in sexually active women in South Africa (2003-2007), in which HIV-1 incidence was estimated at 3.9-4.1 per 100 person-years, and was 7 per 100 person years in Durban and 3.3 per 100 persons years in Johannesburg female placebo recipients aged 18-49 (63).

Based on the above data, the overall estimate of background HIV-1 incidence is 4.0 per 100 person-years, and this level of incidence is expected for the duration of our study. Self-reported risk and presumably incidence decreases during entrance into HIV prevention studies as a result of risk reduction counseling provided to study participants. While incidence of HIV infection may be expected to decrease over time in trial populations due to extensive counseling and/or the increasing availability of alternative HIV prevention modalities, the established incidence of 4% per year in other prevention studies conducted recently in the area represents an average over the entire study period, which indicates that even if incidence declines, the power calculations do not underestimate sample size. In addition, some recent HIV prevention trials in Southern Africa have had steady or slightly increasing HIV incidence over the first 18 months (eg, HPTN 035) (64).

Recent developments in HIV prevention may have an impact on background HIV incidence. Oral Pre-exposure Prophylaxis (PrEP) using the combination antiretroviral drugs emtricitabine and tenofovir disoproxil fumarate (FTC/TDF)

has proven to be an efficacious user-controlled prevention intervention. While the MCC has approved FTC/TDF for use as PrEP, this combination antiretroviral agent is not yet available for this use in the public sector in South Africa. It is available by prescription, in demonstration projects, and in the private sector. Participation in this trial is not precluded by oral PrEP use. In addition, medical male circumcision will be advocated as part of risk reduction counseling at all sites. The START (Strategic Timing of AntiRetroviral Treatment) study has confirmed that immediate referral for ART is beneficial to participants who are newly diagnosed with HIV (65) and HPTN 052 demonstrated the value of treatment in reducing secondary transmission in discordant couples (66). An initiative to implement universal test and treat was announced in the Republic in September 2016. Additional modes of HIV prevention (eg, topical microbicides, injectable ARVs, etc.) are currently being studied in efficacy trials and may become available while HVTN 702 is underway. Study participants will be informed of such developments and uptake will be tracked in order to estimate their impact on HVTN 702.

4.5.3 Schedule

Safety and immunogenicity will be assessed for ALVAC-HIV (vCP2438) (months 0, 1, 3, 6, 12, 18) and for Bivalent Subtype C gp120/MF59 (months 3, 6, 12, 18).

The vaccine administration schedule encompasses the schedule used in RV144 but with additional boosts at months 12 and 18 in response to the apparent wane in efficacy from 12 to 36 months observed in RV144.

Addition of a booster vaccination at month 12 is supported by immunogenicity data from nonhuman primate (NHP) studies performed with another pox-protein regimen. Both neutralizing and binding Ab levels dropped after completion of the primary immunization, while the re-boost at week 49 was able to bring back the Ab responses to the higher level (67). In addition, administration of a late ALVAC-HIV/AIDSVAX boost (in RV 305) approximately 7-9 years after the final vaccination in RV144 demonstrated that immune responses were restored or improved compared to the responses observed at the year 3.5 primary timepoint in the RV144 trial (53). The addition of a boost at month 18 is supported by durability data from HVTN 100 (Section 4.2.4)

The selected dose schedule is therefore a 6-dose schedule; a 4-dose primary vaccination schedule with ALVAC-HIV administered at 0, 1, 3 and 6 months plus protein boost administered at 3 and 6 months (replicating the RV144 schedule); and additional booster vaccinations with both ALVAC-HIV (vCP2438) and Bivalent Subtype C gp120/MF59 administered at 12 and 18 months.

4.5.4 Prime-boost regimen

The prime-boost strategy consists of ALVAC-HIV (vCP2438) primes with ALVAC-HIV (vCP2438) plus Bivalent Subtype C gp120/MF59 boosts.

The concept of priming with one HIV vaccine followed by boosting with another heterologous HIV vaccine emerged in the 1990s as a strategy to enhance vaccine immunogenicity and, potentially, vaccine efficacy. While Env glycoproteins elicit strong humoral responses, they have not been associated with potent cellular immunity or efficacy when administered alone. On the other hand, although viral vector constructs, including ALVAC, often elicit both Ab and cell-mediated responses, it was observed that the level of Ab could be increased significantly by subsequent administration of Env glycoproteins. In addition, prime-boost vaccine regimens were explored as means of increasing the breadth of HIV strains to which vaccination might induce responses. Response breadth and the capacity to induce both potent CTL and potent Ab responses is a combination believed to be important in conferring protective immunity against HIV infection (68, 69). These desirable immune response characteristics were often observed in early phase clinical trials using a variety of prime-boost vaccine regimens (69-79).

Among the many prime-boost vaccine regimens tested during this period were canarypox-derived (ALVAC) vector primes containing differing arrays of recombinant HIV genes and HIV Env glycoprotein boosts. By 1998, early phase clinical trials of ALVAC-protein regimens had shown:

- These regimens with glycoproteins adjuvanted with alum or MF59 were well tolerated in several hundred HIV-uninfected, healthy volunteers;

- HIV-specific immune responses were not dampened in vaccinia-experienced individuals;

- The prime-boost regimens induced large repertoires of HIV-specific Ab, neutralizing activity against some laboratory HIV isolates, Ab capable of inducing antibody-dependent cellular cytotoxicity (ADCC), and HIV-specific CTL activity against a broad range of targets (69, 80).

In NHP studies, heterologous prime-boost ALVAC-protein regimens afforded better protection against viral challenge than did sequential injections of ALVAC-HIV vaccines alone, suggesting a potential connection between the characteristic responses elicited by the heterologous ALVAC-protein prime-boost regimens and protection from HIV infection (81).

On the basis of results from nonclinical challenge studies and early-phase clinical trials, ALVAC-HIV (vCP1521) and HIV gp120 Env became the first heterologous prime-boost vaccine regimen to enter efficacy testing in humans in the RV144 trial in Thailand. Comparison of the immunogenicity observed in the RV144 trial to that from the VAX003 efficacy study previously conducted in Thailand, showed that the nAb response after two doses of gp120 protein following priming with ALVAC-HIV (vCP1521) [19] exceeded the nAb response seen after two inoculations with the same bivalent gp120 protein without preceding ALVAC-HIV (vCP1521) (in VAX003) [55], pointing to a priming effect by ALVAC-HIV (vCP1521) that benefited the immune response in the RV144 study (82). Hence, one of the primary advantages to prime-boost regimens

posited by nonclinical studies and supported in early phase clinical trials, was borne out in comparison of immunogenicity results from 2 large later-phase clinical trials conducted in the same country.

Furthermore, recent data suggest that priming by ALVAC-HIV (vCP1521) may favourably influence the quality of Ab responses. The IgG3 HIV-1 Env responses induced by the ALVAC-protein prime-boost regimen were significantly higher than those induced by the homologous gp120 prime-boost regimen, indicating that ALVAC priming before a protein boost modifies the IgG subclass profile in a manner that may substantially change the HIV-1 inhibitory functions of the vaccine-elicited IgG responses (41, 42). IgG1 and IgG3 are the most functional of the IgG subclasses in that they have been associated with HIV-1 neutralization, complement fixation, phagocytosis, ADCC, and antibody dependent cellular viral inhibition (ADCVI) (83).

4.5.5 Choice of control

The control for ALVAC-HIV (vCP2438) and for Bivalent Subtype C gp120/MF59 is sodium chloride for injection, 0.9%.

4.6 Combination prevention of HIV acquisition

HVTN 702 embraces the highest South African Standards of Prevention for all participants. Participants will be provided with a comprehensive HIV prevention package consistent with all DAIDS-sponsored HIV prevention clinical trials. This package includes evidence-based behavioral risk reduction counseling (84), advocacy and referral for medical male circumcision as appropriate, free condoms and lubricant (where available), regular testing for sexually transmitted infections (STIs), counseling and referral for postexposure prophylaxis (PEP) when indicated, and, where appropriate, access to oral drugs for pre-exposure prophylaxis (PrEP, see below). Participants will be informed as new prevention modalities are proven effective and become available. These activities can be expected to reduce HIV acquisition below historical levels. The contribution of these efforts will be monitored. Further details on these efforts are provided in the HVTN 702 Study Specific Procedures (SSP) and the HVTN 702 website.

4.6.1 PrEP in South Africa

South Africa recently registered FTC/TDF for prevention and is developing guidelines for its use. These guidelines stipulate that PrEP must be delivered in the service delivery setting with the main guiding principle of informed and voluntary choice and equitable access. FTC/TDF for HIV prevention is not yet on the Essential Drug List in South Africa, which is a pre-requisite for use in the public sector through which the vast majority of South Africans secure health care. However, the South African government through the National Department of Health (NDOH) intends to conduct demonstration projects, in conjunction with

key donors, in key populations such as sex workers and men who have sex with men (MSM), as well as in geographic locations that have a high burden of HIV.

The World Health Organization has issued updated PrEP guidelines (85) and an investment case evaluating these guidelines has been undertaken by the South African government (86). While the investment case for universal test and treat is clear, and this will be rolled out as a priority, the investment case for PrEP is less certain and is being evaluated.

For these reasons, the protocol team plans to work collaboratively with national regulatory authorities and other government agencies to develop mechanisms for PrEP access that are supportive of and consistent with national priorities and strategic objectives.

Given the potential of FTC/TDF to prevent HIV acquisition, it could be argued that substantial uptake of PrEP could prevent the HVTN 702 trial from detecting any benefit of vaccine administration beyond the universal prevention package. This possibility is addressed in the sample size and power calculations (see Section 6.4.1) and in plans for trial monitoring by the DSMB (see Sections 6.5.5 and 6.5.6). If the background HIV incidence rate is too low to support evaluation of vaccine efficacy, the DSMB will detect this and will make recommendations to the team accordingly. The study team will monitor PrEP availability and roll-out in-country and within the protocol will record use of PrEP drugs as concomitant medications and will evaluate individual and study population PrEP use through direct biomedical measures (see Section 11.7). In support of a comprehensive package of prevention, PrEP will be discussed in the informed consent process, in risk reduction counseling sessions, and referral systems into demonstration projects, health service delivery settings, and the private sector will be developed to support those who wish to access PrEP.

4.7 Nonclinical studies

4.7.1 ALVAC-HIV

4.7.1.1 IM local tolerability and systemic toxicity study in New Zealand White Rabbits (Study AB20670)

The objective of the study was to determine the local tolerability and systemic toxicity of ALVAC-HIV (vCP2438)/ALVAC-HIV (vCP2438) with gp120+MF59 vaccines and DNA-HIV-PT123 with gp120+MF59 vaccines administered by the IM route to New Zealand White Rabbits 7 or 6 times, respectively, at 2-week intervals, followed by a 2-week recovery period.

There were no deaths during the study. No treatment-related clinical signs were reported during the study and treatment was locally well tolerated. Body temperature was slightly increased mostly after the 1st injection but returned to normal within 48 hours. There were no effects of treatment on body weight or

food consumption. No treatment-related ophthalmological findings were observed at the end of the treatment period.

When compared to the control group, a transient increase in C-reactive protein, globulin, fibrinogen, or neutrophil count was observed after one or more of the immunizations. These effects correlated with the inflammatory findings observed histopathologically at the injection sites. There were no other treatment-related differences from the controls amongst the biochemistry or hematological parameters.

At necropsy, at the end of treatment, the only histologic changes due to the test items were in the injection sites and iliolumbar and sacral lymph nodes. In the sites injected with ALVAC or gp120s+MF59, the changes comprised inflammatory cell infiltrates, necrosis, fibrosis, hemorrhage, acellular material and mineralization. These findings were often only minimal or slight. In the lymph nodes which drained the injected sites, there was minimal or slight increased lymphoid follicle development, increased paracortex and granulocyte infiltrate. There was evidence of partial resolution of the described changes at both injection site and lymph nodes, based on necropsy observations after the recovery period.

In conclusion, under the defined study conditions, 7 intramuscular administrations of ALVAC-HIV (vCP2438) vaccine associated with gp120s/MF59 (last 4 injections) to the New Zealand White Rabbit at two-week intervals were clinically and locally well tolerated. The study supports the use of this vaccine regimen in human clinical trials.

4.7.1.2 Other supportive nonclinical safety studies

The nonclinical safety data from a variety of ALVAC constructs further inform the safety profile of ALVAC-HIV (vCP2438). These studies include the following:

- Platform biodistribution study of ALVAC-HIV in rats
- ALVAC viral replication in different cell lines
- Virulence of the ALVAC vector versus Vaccinia strains
- Single dose toxicity studies by intravenous route with various ALVAC recombinants in mice and rats
- Repeated dose toxicity studies using several routes of administration (including IM) with various ALVAC recombinants in cynomolgus and rhesus macaques
- Local tolerance and sensitization studies with various ALVAC recombinants in rabbits
- Hypersensitivity study with ALVAC-HIV (vCP125) in guinea pigs

The results of these studies show a satisfactory nonclinical safety profile and support the administration of the ALVAC-HIV (vCP2438) construct to humans. For additional information, see the ALVAC-HIV (vCP2438) IB.

4.7.2 Toxicity studies of HIV Env vaccines

The nonclinical safety of 4 doses of the gp120 proteins when co-administered with ALVAC-HIV (vCP2438) as a boost after 3 doses of ALVAC-HIV (vCP2438) given alone (prime), was evaluated in study AB20670 in New Zealand White Rabbits, as mentioned in Section 4.7.1.1. Overall, the immunizations were clinically and locally well tolerated.

The nonclinical safety of 6 doses of the gp120 proteins, when co-administered with DNA-HIV-PT123, was also evaluated in study AB20670 in New Zealand White Rabbits. There were no deaths during the study. No treatment-related clinical signs were reported during the study and treatment was locally well tolerated. There was no obvious effect on body temperature. A lower body weight gain was noted in males only over the study period. However this was not related with lower food consumption. No treatment-related ophthalmological findings were observed at the end of the treatment period. When compared to the control group, a slight transient increase in C-reactive protein concentration was noted mainly after the first administration. These effects correlated with the inflammatory findings observed histopathologically at the injection sites. An increase in creatine kinase was noted after the first administration only. At necropsy, at the end of treatment, the only histologic changes due to the test items were in the injection sites and iliolumbar lymph nodes. In the sites injected with DNA-HIV-PT123 or gp120s+MF59, changes comprised fibrosis, hemorrhage, acellular material and inflammatory cell infiltrate usually minimal or slight, but occasionally more severe. In the lymph nodes which drained the injected sites, there was minimal to moderate increased paracortex and increased lymphoid follicle development and minimal granulocyte infiltration. There was evidence of partial resolution of the described changes at both injection site and lymph nodes, based on necropsy observations after the recovery period. In conclusion, under the defined study conditions, 6 intramuscular administrations of DNA-HIV-PT123 vaccine associated with gp120 proteins adjuvanted with MF59 to New Zealand White Rabbits at two-week intervals were clinically and locally well tolerated.

In addition, nonclinical *in vivo* Good Laboratory Practice (GLP) toxicology studies were conducted with early candidate subtype B and E gp120 Env protein vaccine candidates that were subsequently advanced to phase 1-2 clinical trials. More recently, similar subtype B gp140 and subtype C gp140 vaccine candidates with MF59 have been tested in nonclinical safety studies. The subtype C gp140 previously tested was from the same strain (HIV-1 TV1) as one of the components (TV1.C gp120) in the proposed Bivalent Subtype C gp120/MF59 vaccine, and hence is very similar in sequence. Overall, toxicology studies indicated that both the subtype B gp140 and subtype C gp140 vaccines with MF59 were well tolerated and testing revealed no adverse local or systemic effects.

Data from the following nonclinical studies are included in the IB:

- Subchronic IM toxicity study of Biocine HIV Thai E gp120/SF2 gp120 vaccine in rabbits
- Repeat dose toxicity of IM HIV DNA/PLG prime followed by IM subtype B gp140/MF59 in rabbits
- Repeat dose toxicity of intranasal (IN) subtype B gp140 with an LTK63 adjuvant followed by IM subtype B gp140 with MF59 in rabbits
- Repeat dose toxicity of IM South African AIDS Vaccine Initiative (SAAVI) DNA-C2 followed by IM SAAVI MVA-C with subtype C gp140/MF59 in rabbits

4.7.2.1 Toxicity studies of MF59

The nonclinical safety of MF59 co-administered with the gp120 proteins included in HVTN 702 in the context of ALVAC-HIV (vCP2438) was evaluated in study AB20670 as described in Sections 4.7.1.1 and 4.7.2. Overall, the immunizations were clinically and locally well tolerated in New Zealand White Rabbits.

MF59 is not associated with any potential for systemic toxicity and it has a low order of local reactogenicity. In repeat-dose rabbit studies, clinical pathology findings of increased fibrinogen and minor inflammatory and degenerative changes at the injection site are consistent with the effects of IM injections of an immunological adjuvant. These findings are readily reversible within days to 1 to 2 weeks. In repeat-dose toxicology studies in dogs, there were no effects on cardiovascular or central nervous system (safety pharmacology) parameters. MF59 is not genotoxic (Ames test) or clastogenic (mouse micronucleus), is not a dermal sensitizer (Guinea pig), and was not teratogenic (rat and rabbit) or a developmental toxicant (rat).

Pivotal toxicology studies performed with MF59 include:

- single- and repeat-dose toxicity (including local tolerability),
- genotoxicity,
- sensitization, and
- embryofetal and developmental toxicity.

4.8 Plans for future product development and testing

HVTN 702 is designed to provide the efficacy data in support of a potential Marketing Authorisation Application in the Republic of South Africa. It is

anticipated that the efficacy data may be supplemented with data from additional safety and immunogenicity studies evaluating clinical lot-to-lot consistency, bridging to the adolescent age group, and, if required, addressing changes in vaccine manufacturing process and formulation.

4.9 Clinical studies

4.9.1 Clinical studies with related ALVAC-HIV vaccines

The proposed ALVAC-HIV vaccine candidate specific for southern Africa is ALVAC-HIV (vCP2438). The vaccine is closest to ALVAC-HIV (vCP1521) (the ALVAC-HIV used in the RV144 trial) since it contains the same *gag, pro* and *gp41env TM* components. However, it has been adapted to include ZM96 gp120 *env* insert (subtype C) (rather than the TH023 gp120 *env* insert [subtype E] used for the RV144 study regimen in Thailand).

ALVAC-HIV (vCP2438) is currently being investigated in the ongoing trial HVTN 100 (Section 4.9.6).

Extensive previous experience with other ALVAC-HIV vaccines informs the expected safety, tolerability, and immunogenicity profile of the new vaccine (Table 4-6). In all, more than 10,000 clinical trial participants have received ALVAC-HIV vaccines. The majority of these trials were performed with ALVAC-HIV (vCP205), (vCP1452), or (vCP1521). In these vaccines, different HIV gene inserts have been introduced into the ALVAC vector.

ALVAC-HIV (vCP205) is an ALVAC vector vaccine with genetic inserts of the HIV-1 *gag* gene (expressing the Gag p55-polyprotein of the HIV-1 LAI strain [clade B]), a fragment of the *pol* gene (that expresses the p15 Protease of the HIV-1 LAI strain), and a portion of the *env* gene (expressing the gp120 Env glycoprotein of the HIV-1 MN strain [clade B], and the anchoring TM region of gp41 of the HIV-1 LAI strain). The HIV genes are inserted in the C3 locus.

ALVAC-HIV (vCP1452) vaccine is a preparation of a modified recombinant canarypox virus expressing the products of the HIV-1 *env* (Env gp160 protein of the HIV-1 MN Strain [clade B]) and *gag* (HIV-1 LAI strain [clade B]) genes, the protease portion of the *pol* gene on a synthetic polynucleotide encompassing the known human CTL epitopes from the *nef* (BRU Strain), and the *pol* (LAI strain) gene products. The C3 locus was used for the insertion of the HIV-1 *env* and *gag* gene sequences and the C5 locus was used for the insertion of the sequences encoding the HIV-1 Nef and Pol CTL epitopes.

Table 4-6 Recombinant ALVAC-HIV vaccine in human adult prevention trials

Candidate vaccine	# receiving ALVAC-HIV	Protocol	Status
ALVAC-HIV (vCP125)	20	ANRS VAC01	Completed
ALVAC-HIV (vCP125) (low and high dose)	92	AVEG 012A/012B	Completed
ALVAC-HIV (vCP205)	25	ANRS VAC03	Completed
ALVAC-HIV (vCP205) (low and high dose)	185	AVEG 022/022A	Completed
ALVAC-HIV (vCP205)	22	AVEG 029	Completed
ALVAC-HIV (vCP205)	56	AVEG 027	Completed
ALVAC-HIV (vCP205)	290	HVTN 203	Completed
ALVAC-HIV (vCP205)	56	AVEG 032	Completed
ALVAC-HIV (vCP205)	280	AVEG 202/ HIVNET 014	Completed
ALVAC-HIV (vCP205)	30	AVEG 033	Completed
ALVAC-HIV (vCP205)	20	HIVNET 007	Completed
ALVAC-HIV (vCP300)	20	ANRS VAC07	Completed
ALVAC-HIV (vCP300)	119	AVEG 026	Completed
ALVAC-HIV vector (vCP205, 1433, 1452)	20/35/35	AVEG 034	Completed
ALVAC-HIV (vCP205/1452)	15/40	AVEG 034A	Completed
ALVAC-HIV (vCP1452)	22 + 3	ANRS 010	Completed
ALVAC-HIV (vCP1452)	160	HVTN 203	Completed
ALVAC-HIV (vCP1452)	120	HIVNET/HVTN 026	Completed
ALVAC-HIV (vCP1452)	100	HVTN 039	Completed
ALVAC-HIV (vCP1521)	203	RV132/135	Completed
ALVAC-HIV (vCP1521)	8197	RV144	Completed
ALVAC-HIV (vCP1521)	135*	RV 305	Completed
ALVAC-HIV (vCP1521)	327**	RV 306	In progress
ALVAC-HIV (vCP1521)	80*	HVTN 097	Completed
ALVAC-HIV (vCP2438)	210	HVTN 100	In progress
**Total**	**10.917**		

* Primary study completed; extension in progress.

** Planned study product sample size. Study remains blinded.

ALVAC-HIV (vCP1521) vaccine was generated by co-insertion of genes encoding HIV-1 gene products into the ALVAC genome in the C6 locus. The inserted HIV-1 gene sequences are: the region of the *env* gene encoding the extracellular Env gp120 moiety of TH023 strain of HIV-1 (clade E) linked to the sequences encoding the HIV-1 TM anchor sequence of gp41 HIV-1 LAI strain (clade B); the *gag* gene encoding the entire Gag p55-polyprotein of the HIV-1 LAI strain; and a portion of the *pol* sequences of the LAI strain of HIV-1 sufficient to encode the protease function.

ALVAC-HIV (vCP2438) is described in Section 4.3.

4.9.2 Clinical safety experience with related ALVAC-HIV vaccines

4.9.2.1 Summary of safety, reactogenicity, and tolerability from related human experience

The tolerability and safety of ALVAC-HIV (vCP1521) were evaluated initially in 2 phase 1-2 studies in Thailand (87, 88). The most relevant data, however, come from the large RV144 efficacy study performed in Thailand (19, 89), during which more than 8000 participants received the vaccine and ALVAC-HIV (vCP1521) was found to be safe and well tolerated. Vaccine recipients experienced local and/or systemic reactions significantly more frequently than placebo recipients; the frequencies of local reactions such as pain and tenderness were higher than those of systemic reactions such as headache, fatigue, arthralgia and myalgia; fever was rarely reported; ALVAC-HIV (vCP1521) was associated with a higher frequency of local reactions compared to the protein subunit used in the study (AIDSVAX B/E); the frequency of both local and systemic reactions gradually declined with subsequent vaccine administrations; most local and systemic reactogenicity symptoms were mild to moderate, resolving rapidly and spontaneously in the vast majority of cases; and the frequencies of adverse events (AEs) and serious adverse events (SAEs) were not different between vaccine and placebo groups (89). Overall, results with ALVAC-HIV (vCP1521) are consistent with other ALVAC-HIV constructs, supporting the conclusion that the safety, reactogenicity, and tolerability profile of ALVAC-HIV is determined in greater measure by the vector than by the HIV genetic material inserted into it.

De Bruyn et al (90) characterized the tolerability and safety profile of ALVAC-HIV (vCP205) and ALVAC-HIV (vCP1452) (along with other ALVAC vectors) based on data from more than 1,000 clinical trial participants. The authors concluded that:

1. ALVAC-HIV vaccines were safe and well tolerated, with a reactogenicity profile comparable to that of existing vaccines licensed for use in adults; and
2. Reactogenicity was similar for different ALVAC-HIV constructs, suggesting that reactogenicity is determined in greater measure by the vector than by the additional genetic material inserted into the vector.

Of interest as well was the observation that reactogenicity seemed to differ according to certain demographic variables: Black, non-Hispanic participants reported significantly less reactogenicity than did White, non-Hispanic participants; and males reported less pain than females.

Additional information is available in the ALVAC-HIV (vCP2438) IB.

4.9.2.2 Summary of pregnancy occurrence and outcomes

In study RV144 a total of 967 (30.6%) vaccine (vCP1521) and 955 (30.1%) placebo recipients reported a pregnancy during the study while 139 vaccine and 116 placebo recipients reported more than one pregnancy. Birth was reported for 1843 infants, 14 of them representing 7 twin pairs. Of these, 277 births (137

vaccine and 140 placebo recipients; 1 twin pair per treatment) occurred within 450 days of study entry. For these infants, birth weight, gestational age, and Apgar scores were similar between the vaccine and placebo groups. Three congenital abnormalities (1 vaccine and 2 placebo recipients) were reported among these 277 births, the vaccine group abnormality being a respiratory distress syndrome with patent *ductus arteriosus*. Abnormal pregnancy outcomes were experienced in 165 out of 3165 (5.2%) vaccine female recipients and 139 out of 3169 (4.4%) placebo female recipients (p = 0.13), and in 17.1% and 14.6% (p = 0.13) of vaccine and placebo pregnancies, respectively (89).

A total of 15 of the 245 female participants became pregnant during the phase 1-2 AVEG studies. Twelve participants received ALVAC-HIV (vCP205) and 3 received placebo. Of these 15 participants aged 19 to 37 years, 9 participants had live births, 3 participants had elective abortions, 1 subject had a spontaneous abortion, and the outcome of 2 pregnancies remains unknown. Of the 9 live births, 3 were by cesarean section. Overall, no complications during pregnancy or congenital abnormalities at the time of birth were reported.

In HVTN 203 (91) there were 2 participants with miscarriages among the total of 80 female study participants who received ALVAC-HIV (vCP1452). These events were classified as unrelated to the study product by the investigators.

4.9.2.3 Summary of safety and tolerability data in African studies that have used ALVAC-HIV

To date, 3 studies have been completed in Africa with ALVAC-HIV: HIVNET 007, HPTN 027, and HVTN 097.

HIVNET 007 (92) was a randomized, double-blind, placebo-controlled clinical trial conducted in Kampala, Uganda. In this study, 40 HIV-seronegative Ugandan volunteers were randomly assigned to receive ALVAC-HIV (vCP205) (n = 20), control ALVAC containing the rabies virus glycoprotein G gene (n = 10), or saline placebo (n = 10). Adverse reactions to immunizations were similar to those in previous trials with these vaccines in HIV-seronegative volunteers in the United States. No severe (grade 3 or 4) adverse reactions attributable to receipt of the vaccine were observed.

HPTN 027 (93) was a phase 1 randomized, single center, double-blind, placebo-controlled trial that evaluated the safety and immunogenicity of ALVAC-HIV (vCP1521) in infants born to HIV-1–infected women in Uganda. 60 infants were enrolled with 48 in the active group and 12 in the placebo group. Forty-seven infants received all 4 vaccinations and completed follow-up (38 in the vaccine group and 9 in the placebo group). There were 3 deaths in the HPTN 027 study (2 in the vaccine group and 1 in the placebo group); all were reported as unrelated to the vaccine. The deaths included pneumonia-like illness, cor pulmonale secondary to congenital heart disease complicated by pneumonia, and gastroenteritis complicated with electrolyte imbalance. The rate of SAEs was similar between groups (56% in the vaccine group and 50% in the placebo group). In addition to

the 3 deaths, 4 were discontinued from vaccination due to HIV infection (of whom 3 were HIV-positive at birth and 1 HIV-positive at 2 weeks; no other infant was found to be HIV-positive in the study); 1 was lost to follow-up; 6 (5 vaccine, 1 placebo) had AE requiring discontinuation (3 CD4 <25, anemia, gastroenteritis, increased liver enzymes). Thirteen infants in HPTN 027 experienced an AE that led to discontinuation of vaccinations: 10 participants in the vaccine group and 3 in the placebo group. There were no severe or life-threatening reactogenicity events. Mild reactogenicity events were common in both study arms, with only 1 moderate event (irritability) in the placebo group and 7 in the vaccine group (erythema, induration, pain, fever, and irritability).

In addition, the primary protocol for HVTN 097, which administered the RV144 vaccine regimen in a "low risk" South African population, is complete (Section 4.9.5).

4.9.2.4 Previous human experience with ALVAC-HIV used in combination with subunit protein boost adjuvanted with MF59

In addition to the ongoing HVTN 100 trial (Section 4.9.6), there has been meaningful previous human experience with the use of ALVAC-HIV vaccines in combination with recombinant gp120 proteins adjuvanted with MF59. In all, 440 participants had received this combination across 7 clinical trials before initiation of HVTN 100 (Table 4-7). No safety signal of concern was identified in these studies.

Table 4-7 Clinical studies performed with ALVAC-HIV and gp120+MF59

Study (Country)	ALVAC-HIV	Protein	Participants[a]
RV132 (88) (Thailand)	vCP1521	gp120 + MF59 clades B/E made in CHO cells	n = 45
AVEG 022A (94) (USA)	vCP205	gp120 + MF59 clade B made in CHO cells	n = 47
AVEG 029 (95) (USA)	vCP205	gp120 + MF59 clade B made in CHO cells	n = 22
AVEG 202/HIVNET 014 (96, 97) (USA)	vCP205	gp120 + MF59 clade B made in CHO cells	n = 145
AVEG 032 (98) (USA)	vCP205	gp120 +/- p24 + MF59 clade B gp120 made in CHO cells p24 made in *S. cerevisiae*	n = 56
AVEG 026 (99) (USA)	vCP300[b]	gp120 + MF59 clade B made in CHO cells	n = 85
AVEG 012A 012B (100) (USA)	vCP125[c]	gp120 + MF59 clade B made in CHO cells	n = 40
**Total**			**N = 440**

[a] Number of participants who received both the ALVAC-HIV prime and the gp120/MF59 boost.

[b] ALVAC-HIV (vCP300) is similar to vCP205 and contains additional sequences encoding Pol and Nef epitopes.

[c] ALVAC-HIV (vCP125) contains the gene for gp160 from clade B.

4.9.3 Immunogenicity from related human clinical trial experience

Immunogenicity measures in ALVAC-HIV studies have evolved over a period of more than 2 decades, informed by evolution in knowledge about relevant immune responses. Initial studies focused on the measurement of CTL activity, CD4+ T cell lymphoproliferation, and nAb activity. Subsequent studies have focused on Ab binding to the Env glycoproteins and on intracellular cytokine staining (ICS) as well. Most recently, a large collaborative consortium performed a case-control study to evaluate immune CoR based on the RV144 study that used the prime-boost regimen of ALVAC-HIV (vCP1521) and the gp120 protein AIDSVAX B/E (Section 4.2.2) (21).

As the development of assays for measuring immunogenicity has evolved during more than 20 years of testing in humans, immunogenicity data cannot be fully integrated. However, extensive data from previous studies with ALVAC-HIV can inform many relevant immunogenicity-related issues, as described below.

4.9.3.1 ALVAC-protein schedule and immunogenicity

The selection of a vaccination schedule for the large efficacy trial in Thailand (RV144) was based on existing scientific knowledge at the time (101). In addition to safety, the key parameters taken into consideration were the CTL immune responses and the nAb responses. The HIV vaccine field has evolved significantly since then and the relevance of these immune measures is currently debated. However, the demonstration of vaccine protection in RV144 mandates the conservation of vaccination regimen features that are believed to have contributed to vaccine protection, even when the mechanism of protection has not been definitely established. The following paragraphs summarize the considerations that were taken into account in the selection of a vaccine regimen and schedule for the RV144 study. Section 4.5.3 explains the rationale for schedule modifications to address the goal of improving upon the RV144 results.

Prior to RV144, several schedules of administration with ALVAC primes and protein boosts were examined in multiple clinical trials (80, 94, 97, 102, 103). While the studies were not designed or powered to discriminate statistically between the various vaccination schedules, an analysis of the data suggested that 4 doses of ALVAC induced better CTL responses than 3 or 2 doses. Specifically, net point prevalence CTL response rates on Days 182 and 273 using 4-dose immunization regimens (Months 0, 1, 3, and 6 or Months 0, 1, 6, and 9) produced higher response rates than the 3-dose regimen (Months 0, 1, and 6) on Days 182 and 273. Regarding neutralization data, both ALVAC schedules (ALVAC alone and ALVAC plus subunit protein boost), showed significantly higher neutralization response rates compared to the control schedule.

The addition of a subunit protein boost to ALVAC did not appear to alter the CTL response rates (103). However, the protein boost did have a significant effect on Ab responses. The ALVAC plus subunit protein boost schedule had significantly higher nAb response rates when compared to the ALVAC alone schedule.

On the basis of these observations, a 4-dose regimen (Months 0, 1, 3, and 6) of ALVAC was proposed in order to maximize CTL responses. In addition, 2 doses of the protein boost were proposed at months 3 and 6 to maximize Ab responses. The RV144 study implemented this vaccination schedule.

4.9.3.2 Immunogenicity of ALVAC used in combination with protein boost plus MF59

The vaccine regimen proposed for development in South Africa combines ALVAC-HIV (vCP2438) with a bivalent recombinant gp120 protein (total of 200mcg, 100mcg of each protein) adjuvanted with MF59. This vaccine regimen is being evaluated in an ongoing study (HVTN 100), but no data are yet available to estimate its expected peak immunogenicity. However, previous studies with related vaccines provide useful preliminary information on whether peak immunogenicity is expected to be at least similar to that elicited by the RV144 regimen and whether the use of MF59 could be dose-sparing for the protein.

Study RV132 (88) used ALVAC-HIV (vCP1521) in the same dose and schedule as in study RV144 but 45 participants in one of the study arms received a bivalent recombinant gp120 protein manufactured in CHO cells by Novartis Vaccines and Diagnostics and adjuvanted with MF59 as a protein boost. The dose of the proteins was 150 mcg in total (100 mcg of the CM235 protein and 50 mcg of the SF2 protein). Study RV135 (87) used ALVAC-HIV (vCP1521) in the same dose and schedule as in study RV144, and 97 participants in two study arms received a bivalent recombinant gp120 protein manufactured in CHO cells by VaxGen/Global Solutions for Infectious Diseases (GSID) and adjuvanted with alum as a protein boost. The study explored two doses of the proteins: a total dose of 200 mcg (100 mcg for the A244 protein and 100 mcg for the MN protein) and a total dose of 600 mcg (300 mcg each of the same proteins in the lower dose formulation). The vaccine regimen with ALVAC-HIV and 600 mcg of gp120 protein adjuvanted with alum was utilized in study RV144. Table 4-8 summarizes the nAb response rates for the pertinent study arms from these studies.

Table 4-8 nAb response rates for selected regimens in RV132 and RV135 studies

Study	gp120 dose	Adjuvant	N	NPO3 strain	SF2 strain	CM244 strain	MN strain	Any clade E
RV132 [78]	100mcg CM235* 50 mcg SF2**	MF59	45	89%	61%	95%	19%	100%
RV135 [87]	100mcg A244* 100mcg MN**	alum	50	23%	--	44%	100%	47%
	300mcg A244* 300mcg MN**	alum	47	31%	--	64%	98%	71%

* clade E Strain

** clade B Strain

The GM nAb titers were also reported in these studies for 2 of the clade E strains. Data are summarized in Table 4-9.

Table 4-9 nAb GM titers to clade E strains

Study	gp120 dose	Adjuvant	N	NPO3 Strain	CM244 Strain
RV132 [88]	100mcg CM235*<br>50 mcg SF2**	MF59	45	45	32.66
RV135 [87]	100mcg A244*<br>100mcg MN**	alum	50	12.3	7
	300mcg A244*<br>300mcg MN**	alum	47	14.8	5.4

* clade E Strain
** clade B Strain

Although the data should be interpreted with caution, they suggest that 100mcg of gp120 protein adjuvanted with MF59 can induce Ab responses after ALVAC prime at least comparable to and possibly greater than 300mcg of gp120 protein adjuvanted with alum after ALVAC prime. These data suggest that MF59 allows for protein dose sparing compared with the alum adjuvant.

4.9.4 Clinical studies with Novartis HIV-1 subunit protein vaccines

Although clinical experience with Bivalent Subtype C gp120/MF59 vaccine is limited to the ongoing HVTN 100 trial (Section 4.9.6), other closely related recombinant monomeric (gp120) subunit vaccine formulations from Novartis Vaccines and Diagnostics (formerly Chiron, now GSK) have been tested in many clinical trials. In addition, recombinant oligomeric (o-gp140) Env proteins for subtypes B and C from Novartis have been or are currently in clinical trials. Overall, in these studies, recombinant HIV-Env proteins manufactured by Novartis were well tolerated and immunogenic. In most cases, recombinant HIV-Env proteins (either gp120 or gp140) were CHO-based and administered with MF59 (50). MF59 safety has been established in clinical studies as well as in commercial products (47, 48). A seasonal influenza vaccine adjuvanted with MF59 (Fluad) is licensed in European Union (EU) countries and in other countries for use in the elderly (104). MF59 is also used in a prepandemic H5N1 influenza vaccine (Aflunov) licensed in EU for use in adults, and in two pandemic H1N1 influenza vaccines (Focetria and Celtura), licensed in EU and other countries for use in adults and children (52). More than 100 million doses of MF59-adjuvanted influenza vaccines have been distributed in licensed products.

Recombinant monomeric (gp120) vaccine candidates studied include Chiron's early gp120-based candidate proteins from subtypes B and E, most of which were CHO-based and administered with MF59. More than 1200 clinical trials participants contributed in the evaluation of the Chiron HIV SF2 gp120/MF59 vaccine and the Chiron HIV CM235 Thai E gp120/MF59 vaccine [55,88,99,105-107]. Three clinical trials were conducted using Novartis CHO-based subtype B gp140 recombinant Env protein with MF59. There are 2 ongoing phase 1 studies with Novartis CHO-based subtype C gp140/MF59 being conducted by the NIH-sponsored HVTN in the US and the RSA. Table 4-10 summarizes clinical trial experience with Novartis gp120 and gp140 recombinant vaccine candidates.

Table 4-10 Novartis recombinant gp120 and gp140 vaccines in human clinical trials* [106]

Candidate vaccine	# receiving Novartis protein	Protocol	Status
Yeast derived recombinant subtype B SF2 Env 2-3 protein with MF59 and MTP-PE	60	AVEG 005 A/B/C	Completed
SF2 gp120 (CHO) with MF59 and MTP-PE	50	AVEG 007 A/B/C	Completed
SF2 gp120 (CHO)/MF59 and ALVAC	40	AVEG 012A 012B	Completed
SF2 gp120 (CHO) with MF59, SAF/2, SAF2 + MDP	107	AVEG 015	Completed
SF2 gp120 (CHO)/MF59 and ALVAC	47	AVEG 022A	Completed
SF2 gp120 (CHO) with MF59	24	AVEG 024	Completed
SF2 gp120 (CHO)/MF59 and ALVAC	85	AVEG 026	Completed
SF2 gp120 (CHO)/MF59 and ALVAC	22	AVEG 029	Completed
SF2 gp120 (CHO) +/- yeast derived p24/MF59 and ALVAC	56	AVEG 032	Completed
SF2 gp120 (CHO) with MF59	126	AVEG201	Completed
SF2 gp120 (CHO)/MF59 and ALVAC		AVEG 202	Completed
SF2 gp120 & CM235 gp120 (CHO)/MF59 and ALVAC	45	RV132	Completed
Subtype B (SF162) gp140 (CHO)/MF59 and Subtype B DNA/PLG	90	HVTN 049	Completed
Subtype B (SF162) gp140 (CHO)/MF59 IN with LTK63	20	C86P1	Completed
Subtype C (TV1) gp140 (CHO) & ISS TAT	30	ISS P-002	Completed
Subtype C (TV1) gp140 (CHO)/MF59	20	HVTN 088	Completed
Subtype C (TV1) gp140 (CHO)/MF59 and SAAVI DNA-C2 and SAAVI MVA-C	24	HVTN073E	Completed
Subtype C (TV1) gp140 (CHO)/MF59 and SAAVI DNA-C2 and SAAVI MVA-C	114	HVTN 086	Ongoing
Bivalent Subtype C gp120/MF59 (TV1.C and 1086.C) and ALVAC	210	HVTN 100	Ongoing
**Total**	**1170**		

In general, these recombinant protein vaccines were immunogenic and well tolerated with no unusual or serious vaccine-associated AEs reported. Most of the reactions were mild to moderate in nature, and of short duration (19, 55, 88, 99, 105-109).

4.9.4.1 Summary of safety, reactogenicity, and tolerability from recent human experience

Five clinical trials have been conducted recently using Novartis Chinese hamster ovary (CHO)-based subtype B gp140 with MF59. A sixth trial using Novartis CHO-based subtype C gp140, HVTN 086, is in extended follow-up (ie, annual health contacts). In addition, the HVTN 100 trial, which administers Bivalent Subtype C gp120/MF59, is currently ongoing (Section 4.9.6).

Among the clinical trials that have been completed, a phase 1 single-center trial (C86P1) was conducted using Novartis CHO-based subtype B gp140 recombinant Env protein in Great Britain by the Mucosal Vaccines for Poverty Related Diseases (MUVAPRED) Consortium to assess safety, tolerability, and immunogenicity of intranasal (IN) administration of subtype B gp140 with and without the mucosal adjuvant LTK63 (detoxified mutant heat labile protein) followed by IM boosting with subtype B gp140/MF59. This study enrolled 30 healthy volunteers aged 18-45, with 20 to receive gp140. The protocol was amended to halt further IN administration of LTK63 following a report of an AE (facial nerve paralysis) with a possible association with the LTK63 adjuvant in another study (110). During the study, there was 1 SAE reported of Bell's Palsy (facial nerve paralysis) considered possibly related to the study vaccine LTK63 in a subject who never received any subtype B gp140 protein or any protein with MF59 adjuvant. IN vaccination was reactogenic resulting in upper respiratory tract symptoms including nasal congestion, nasal discomfort, pharyngolaryngeal pain, and rhinorrhea. The subtype B gp140 MF59 was well tolerated following IM boost.

Another completed study with Novartis subtype B gp140 MF59 was a multicenter, placebo-controlled trial (HVTN 049) conducted by the HVTN in the United States (111). Participants received one of three doses of a DNA/PLG vaccine (subtype B *gag* DNA/PLG and subtype B *env* DNA/PLG microparticles, at doses of 250/250, 500/500, or 1000/1000mcg) or placebo (5 to 1 ratio) as a single IM injection at 0, 1 and 2 months, followed by a boost of subtype B gp140 with MF59 (or placebo) at 6 and 9 months. An additional group of participants received subtype B gp140 with MF59 without DNA prime, administered at 0, 3, and 9 months. Overall 96 healthy, HIV-1–uninfected adult participants were enrolled and 86 participants completed all planned vaccinations. There were no SAEs reported as related to study vaccine. There were four events reported as SAEs that were not considered related to the study vaccine. A death attributed to cocaine overdose occurred in one subject, 10 days after receipt of the second dose of the placebo. One subject had a Grade 3 increase in creatine phosphokinase (CPK) to 2311 U/L, 14 days after the first DNA prime vaccination, which resolved within a week. Another subject had a Grade 4 increase in CPK to 4806 U/L 15 days after the first DNA prime vaccination, which resolved within two weeks. Both participants reported to have initiated new exercise programs. One subject experienced severe fatigue 20 days after the fourth immunization (including one dose of subtype B gp140/MF59), attributed to working two jobs and long hours. Overall, the regimens were generally well tolerated.

A third trial, HVTN 088, was conducted in the United States in order to evaluate the safety and immunogenicity of a long-interval, cross-clade subtype C gp140/MF59 boost in participants administered subtype B gp120/MF59 or subtype B gp140/MF59 in previous trials. This included participants from the HVTN 049 DNA/PLG prime, gp140/MF59 boost study described above. The study enrolled 16 previously vaccinated participants and 20 naive controls. Individuals were identified who had received a clade B Env protein with MF59 4-17 years earlier, most in combination with a DNA or ALVAC prime. These

individuals were enrolled in HVTN088 to receive a clade C protein boost in an open label phase 1 trial. The study was modified to add an additional late boost of the clade C gp140/MF59 vaccine. There were 3 SAEs reported in this trial, one involving traumatic injury, one instance of gastroenteritis, and one of appendicitis. All of these were assessed as unrelated to study agents.

Another study, HVTN 073E, was conducted in the US and RSA as an extension to the previous HVTN 073/SAAVI03 study. This extension study examined the safety and immunogenicity of two boosting doses of Novartis subtype C gp140/MF59 or placebo in participants who previously received 3 vaccinations of SAAVI DNA-C2 and two vaccinations of SAAVI MVA-C. This study enrolled 27 participants. There was one report of endometrial intra-epithelial neoplasia resulting in hospitalization for hysterectomy, which was assessed as unrelated to study agents.

The Istituto Superiore di Sanità (ISS) study (ISS P-002) conducted in Italy examined the safety and immunogenicity of subtype C gp140 co-administered with ISS TAT compared to subtype C gp140 alone or TAT alone. The study includes intradermal and IM injections (100 mcg for subtype C gp140 and 7.5 mcg for ISS TAT). This study did not include MF59. No SAEs were reported in this study.

The study in extended follow-up, HVTN 086, is being conducted in the RSA. It is evaluating the safety and immunogenicity of various combinations of SAAVI DNA-C2, SAAVI MVA-C, and Novartis subtype C gp140/MF59. This study enrolled 184 participants. To date, 6 SAEs have been reported in this study, one case of acute tonsillitis that required hospitalization, one of schizophrenia requiring hospitalization (later determined to be a pre-existing condition), one of pelvic inflammatory disease, one soft-tissue injury, one instance of anemia, and one instance of alcohol-related cardiomyopathy. All were assessed as not related to the study products. During the annual health contact period, suspected unexpected serious adverse reactions (SUSARs) are collected; none have been reported thus far for HVTN 086.

4.9.4.2 Summary of immunogenicity from recent human experience

The immunogenicity of Novartis recombinant proteins has been demonstrated consistently in all clinical trials and in both of the recently completed studies using Novartis CHO-based subtype B gp140 MF59. In the HVTN 049 DNA/PLG prime protein boost study, the primary cellular immunogenicity endpoints included interferon gamma (IFN-γ) enzyme-linked immunospot (ELISpot) and ICS responses. Immunogenicity was assessed 14 days after each vaccination. Env-specific IFN-γ ELISpot response rates did not increase substantially compared to baseline after the three DNA/PLG prime vaccinations, but did rise after the first protein/MF59 boost. nAb titers against the homologous SF162 isolate were detectable in two vaccinees after the third DNA/PLG priming vaccination and in 13 vaccinees after the first protein boost. Neutralization was boosted to high titer in all but one vaccinee following the second protein boost.

Similarly in the group of participants who received subtype B gp140/MF59 without a DNA/PLG prime, a nearly complete response to the SF162 isolate was observed at the second vaccination (all but one vaccinee) which lasted through the third vaccination. Binding Ab titers against Env, measured by enzyme-linked immunosorbent assay (ELISA), were detected following the first subtype B gp140/MF59 boost and were very high following the second boost administration.

The C86P2 Mucosal Vaccines for Poverty Related Diseases (MUVAPRED) IN study demonstrated immunogenicity with considerable IgG and IgA Ab responses to subtype B gp140 in serum, cervical, and vaginal secretions of participants following IN administration of subtype B gp140 with the adjuvant LTK63 and an IM boost with subtype B gp140 and M59 adjuvant. nAb responses against the homologous SF162 were also detected in all groups following IM boost with subtype B gp140 and MF59 adjuvant.

In the subtype C gp140/MF59 HVTN 088 long interval boost study, immunogenicity results are available for the vaccinations administered in Version 1 of the protocol. Sixteen previously primed volunteers and 20 naïve volunteers each received 2 doses of the subtype C gp140/MF59 given 6 months apart. HIV-1 specific CD4+ and CD8+ T-cell responses were measured by an ICS assay. Ab responses were measured with a Luminex binding Ab assay and a nAb assay in TZM-bl Cells. Despite the long interval (4-17 years from prior protein/MF59 administration), 31% of primed participants demonstrated CD4+ T-cell responses to Env at baseline, which increased to 75% after a single protein boost. IgG and IgA responses to subtype C gp140/MF59 were present in 64% (IgG) and 7% (IgA) of primed participants at baseline, and rose to 93% and 85%, respectively, after one dose of protein. 71% of primed participants demonstrated nAb against Tier 1 clade B isolate MN at baseline. After a single booster dose of protein, 100% of the primed participants neutralized MN and 93% showed neutralizing activity against a clade C isolate, MW965.26. Unprimed participants did not demonstrate CD4+ responses or Ab responses to Env until after the second dose, which elicited IgG and IgA responses to TV1 Env in 88% and 50%, respectively. nAb developed to MN in 38% and to MW965.26 in 88% of the unprimed participants.

4.9.5 HVTN 097

As noted, development of the proposed vaccine regimen for South Africa was predicated on the results obtained in the RV144 trial in Thailand. The HVTN 097 study was designed to confirm that the Thai prime boost would be comparably immunogenic in a South African study population. Hence, immune responses elicited in the South African population were evaluated and compared with those obtained in RV144 vaccinees. Note that, while the ALVAC-HIV (vCP1521) lots administered in the RV144 trial ranged from $10^{7.06}$ $CCID_{50}$ to $10^{7.41}$ $CCID_{50}$, the measured titer of the vaccine lot used in HVTN 097 was $10^{7.72}$ $CCID_{50}$.

The HVTN 097 trial enrolled 100 healthy, HIV-1–uninfected participants aged 18-40 years, 51 male and 49 female. Ninety-one participants completed

vaccinations and follow-up. The study opened in June 2013; it is now complete. The original HVTN 097 schema is shown in Table 4-11. Safety and immunogenicity results reported below are for the original study.

Table 4-11 HVTN 097 schema

Group	N	Month 0 (Day 0)	Month 1 (Day 28)	Month 2 (Day 56)	Month 4 (Day 112)	Month 7 (Day 196)	Month 7.5 (Day 210)	Month 8.5 (Day 238)	Month 13 (Day 394)
1 (T1)	60	Tetanus toxoid	ALVAC	ALVAC	ALVAC + AIDSVAX B/E	ALVAC + AIDSVAX B/E	HBV vaccine	HBV vaccine	HBV vaccine
2 (T2)	20	placebo	ALVAC	ALVAC	ALVAC + AIDSVAX B/E	ALVAC + AIDSVAX B/E	placebo	placebo	placebo
3 (T3)	20	Tetanus toxoid	placebo	placebo	placebo	placebo	HBV vaccine	HBV vaccine	HBV vaccine

4.9.5.1 Safety data

Four participants discontinued vaccinations (DOV), 2 due to pregnancy (both received active vaccine) and 2 due to “other” reason (1 received active vaccine); there were no DOVs due to AEs or reactogenicity. There were 7 early terminations due to participant refusal (3), nonadherence to schedule (3), and unable to contact (1). No participant was terminated early due to an AE.

Local and systemic reactogenicity was assessed for the investigational ALVAC and AIDSVAX vaccinations. Local injection site reactions of pain and/or tenderness were more common in participants receiving active HIV vaccinations versus placebo injections. Most pain and/or tenderness reactions to the HIV vaccinations (T1 and T2 combined) were mild (48%), 28% were moderate, and ~9% were severe. In placebo recipients the maximum pain and/or tenderness reactions were mild (52%). The majority of participants in both active (T1 and T2 combined) and placebo groups experienced no erythema and/or induration reactions (84% and 95%, respectively); with all but 1 reaction (> 9 cm erythema/induration) being non-gradable by the DAIDS AE Grading table (0-25 cm^2), occurring in T1.

Moderate or severe systemic reactions associated with vaccine administration included malaise and/or fatigue (15% vs 5.3% in placebo), myalgia (12.5% vs 0% in placebo), headache (7.5% vs 21% in placebo), nausea (1.25% vs 0% in placebo), chills (3.75% vs. 5.3% in placebo) and arthralgia (6.25% vs 0% in placebo). The maximum temperature elevations were grade 2, which occurred in 2 vaccinees compared to 0 in placebo.

Overall, 88.75% of vaccine recipients experienced at least 1 AE compared to 85% of placebo recipients. There were 2 SAEs and both were unrelated to treatment: thermal burn in a vaccinee and substance-induced psychotic disorder in a placebo recipient. There were no grade 4 (life threatening) or 5 (death) AEs. There were 5 grade 3 AEs in vaccine recipients (6.25%) and 2 grade 3 events in one placebo recipient (5.3%), all deemed unrelated to study treatment. These included alanine

aminotransferase (ALT) increase, headache, hypertension, abnormal loss of weight, and thermal burn in vaccinees and substance induced psychotic disorder and abnormal loss of weight in one placebo recipient. Moderate AEs were experienced by 61.25% of vaccinees and 50% of placebo recipients.

AEs considered related to the vaccine included itching at the injection site, lymph node swelling, faster heartbeat, abdominal pain, flu-like illness, diarrhea, injection site skin lump, and muscle spasms. Each of these reactions was mild or moderate, only occurred in one person (except for the skin lump which occurred in three participants), and was transient. The participants who had them recovered without any problems. A few participants had changes in their laboratory, blood, and urine test results that were considered related to the vaccinations and all returned to normal.

4.9.5.2 Immunogenicity

Cellular/ICS

HIV-specific T-cell responses were measured by intracellular cytokine staining (ICS) at day 210, corresponding to 2 weeks after the second ALVAC + AIDSVAX B/E vaccination (prior to the hepatitis B vaccination). CD4+ and CD8+ T-cell response rates and magnitudes (as measured by production of IL-2 and/or IFN-γ) were evaluated for 92TH023-Env and LAI-Gag peptide pools.

For CD4+ T cells, the vast majority of responses were generated to the antigen 92TH023-Env in groups T1 (Group 1) (72%) and T2 (Group 2) (60%); responses were extremely low to the other two antigens (LAI-Gag and HBsAg) across all treatment groups (< 6%) (Figure 4-11). The response rate to 92TH023-Env was significantly higher in the combined treatment group "T1+T2" compared to group T3 (Group 3) ($p < 0.0001$) and there was no response difference between groups 1 and 2 ($p = 0.4$). Differences in magnitudes of positive responders to antigen 92TH023-Env were non-significant in all group comparisons.

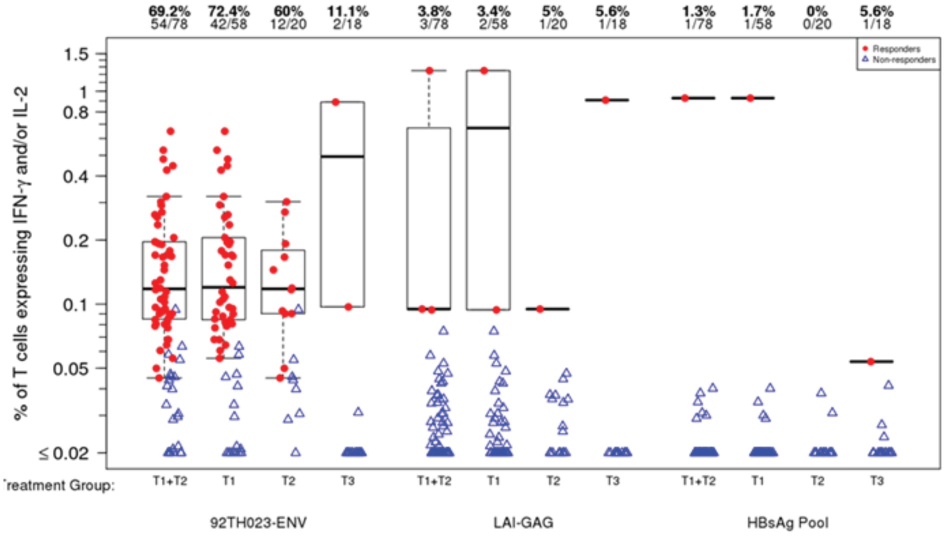

Figure 4-11 HIV-specific CD4+ T cell responses 2 weeks post second ALVAC + AIDSVAX vaccination

Comparisons of T-cell immune responses in vaccinees in HVTN 097 to responses from a random selection of samples from RV144 vaccine recipients adjusting for placebo responses, showed similar results (Table 4-12).

Table 4-12 Comparison of HVTN 097 and RV144 ICS results adjusting for placebo responses % responder (vaccine) - % responder (placebo)

	HVTN 097	RV144	P-value HVTN 097 vs RV144
**CD4+ Env**	58.1% (95% CI = 40.4%, 75.9%)	50.3% (95% CI = 42.9%, 57.7%)	0.425
**CD4+ Gag**	-1.7% (95% CI = -13.1%, 9.7%)	2.5% (95% CI = -2.3%, 7.3%)	0.506
**CD8+ Env**	5.1% (95% CI = 0.2%, 10.0%)	0.6% (95% CI = -0.5%, 1.6%)	0.074
**CD8+ Gag**	2.6% (95% CI = -0.9%, 6.1%)	5.0% (95% CI = -1.8%, 11.8%)	0.530

For CD8+ T cells, only a few responses were observed to Env and Gag, which is also similar to results from RV144 vaccinees.

Another goal of HVTN 097 was to evaluate the effect of body mass index (BMI) on immune responses. Neither BMI nor demographic factors of gender and age significantly affected T cell immune responses.

Binding antibody

The frequency and magnitude of IgA and IgG antibody binding were measured by the HIV-1 binding antibody multiplex assay (BAMA) on serum specimens obtained at baseline (day 0), on day 210, 2 weeks after the second ALVAC + AIDSVAX vaccination, and on day 394, 6 months after the second ALVAC + AIDSVAX vaccination. Antigens for primary analysis include ConS gp140 CFI, MN gp120, A244 gp120, clade A, B, and C consensus Env and V1/V2 antigens.

IgG results:

In general, response rates were significantly higher in group T1+T2 compared to group T3 and there are no significant differences between groups T1 and T2 across all antigens at 2 weeks after the second ALVAC + AIDSVAX vaccination or 6 months after the second ALVAC + AIDSVAX vaccination). In groups T1 and T2, the IgG response rates at peak in general are high for all tested antigens (82-100%) and IgG responses to V1V2 cross-clade antigens (clade A/AE, B, and C) were also high (66-99%), with the greatest magnitude of responses towards clade A/AE V1V2 antigens. At 6 months post the last HIV-specific vaccination, the response remained high to antigen A244 D11gp120_avi, but dropped significantly for all other antigens. The response magnitudes among positive responders at peak waned significantly 6 months post the second ALVAC + AIDSVAX vaccination in all groups. In general, no significant IgG antibody response was observed in group T3 across all antigens. Among IgG responders, and among group pairs where there were sufficient numbers of positive responders for statistical testing, there were no significant differences in magnitudes of responses to any antigen between groups T1 and T2.

Comparisons of binding antibody responses in HVTN 097 vaccine recipients to immune responses from a random selection of RV144 vaccine recipients showed comparable response rates to gp120, V1V2, and gp140 antigens. Binding antibody responses were of higher magnitude in HVTN 097 compared to RV144 vaccine recipients (Figure 4-12).

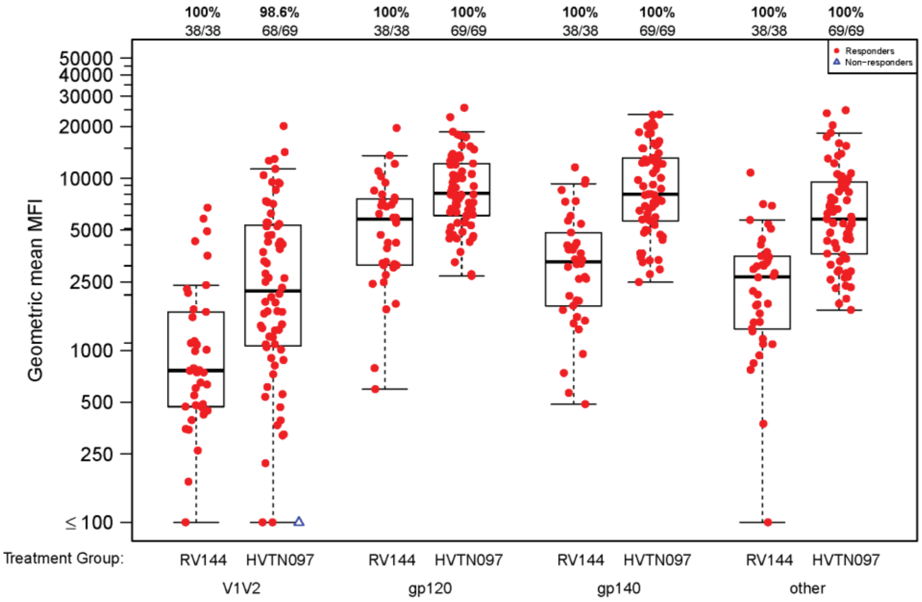

Figure 4-12 Comparison of RV144 and HVTN 097 peak IgG response rates and magnitudes to V1V2, gp120, gp140, and other antigens overall. GM MFI (mean fluorescent intensity) differences were statistically significant between RV144 and HVTN 097 for each antigen group.

As with CD4+ T-cell responses, there were no significant differences in IgG responses to V1V2 antigens within BMI, gender, or age strata in HVTN 097 (Table 4-13).

Table 4-13 IgG responses to V1V2 by BMI, gender or age stratum in vaccine recipients in HVTN 097

Stratum	Responses	Response rate %	95% CI
BMI ≤ 25	40/42	95.2%	(84.2%, 98.7%)
BMI 25-30	16/17	100.0%	(73.0%, 99.0%)
BMI > 30	5/5	100.0%	(56.6%, 100.0%)
Female	28/28	91.7%	(78.2%, 97.1%)
Male	33/36	100.0%	(91.7%, 100.0%)
Age ≤ 20	19/19	100.0%	(83.2%, 100.0%)
Age 21-25	25/28	89.3%	(78.2%, 96.3%)
Age ≥ 26	17/17	100.0%	(81.6%, 100.0%)

IgA results:

At peak , 2 weeks post the last HIV-specific vaccination, IgA responses to most of the antigens are below 50% in group T1+T2, except for antigens A44 D11gp120_avi (85%) and AE.01.con_env03 gp140CF_avi (78%). The differences in response rates and magnitudes across antigens are minimal between groups T1 and T2. At visit 17, 6 months post the last HIV-specific vaccination, response rates range from 0% to 11% and magnitudes dropped to very low levels in the vaccine groups. There is no response observed in group T3 across all IgA antigens at these timepoints.

Neutralizing antibody assays

Neutralization ID_{50} titers were measured by HIV neutralizing antibody assay from specimens obtained at visit days 28 and 210 (visits 3 and 14, respectively), corresponding to the first ALVAC vaccination and 2 weeks after the second ALVAC + AIDSVAX vaccination (Figure 4-13). Neutralizing antibodies against HIV-1 were measured as a function of reductions in Tat-regulated luciferase (Luc) reporter gene expression in TZM-bl cells. The assay measured neutralization titers against a panel of heterologous Env-pseudotyped viruses that exhibit a Tier 1 neutralization phenotype (clade B: BaL.26, MN.3, SF162.LS; clade C: MW965.26; CRF01_AE: NP03.13, TH023.6). Data from blood draw dates outside the allowable visit window and assay results deemed unreliable for analysis by the lab were excluded from the analysis.

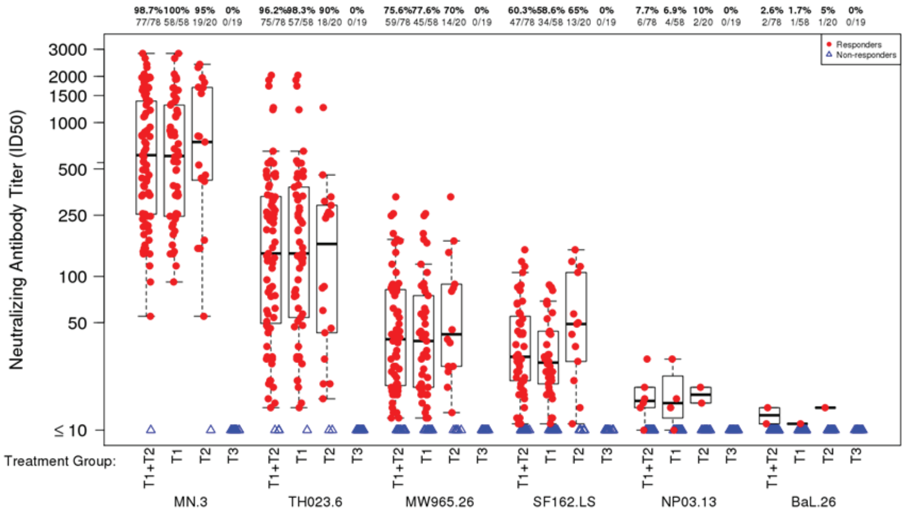

Figure 4-13 Neutralizing antibody responses at visit 14 (2 weeks post second ALVAC + AIDSVAX vaccination)

There were no neutralizing antibody responses to any of the 6 viruses at visit 3. At visit 14, response rates to viruses MN.3, TH023.6, MW965.26 and SF162.LS, were all significantly higher in the groups "T1+T2" and T1 compared to group T3, but no difference was observed between groups T1 and T2. Response rates against Bal.26 and NP03.13 viruses were extremely low (0-10%) and there was no difference between groups. Differences in magnitudes of positive responders were non-significant across all group comparisons. Groups "T1+T2" and T1 had significantly higher AUC- MB when compared to group T3 ($p < 0.0001$ for T1+T2 vs T3, T1 vs T3), and no difference in AUC-MB was detected between groups T1 and T2 ($p = 0.9909$).

Neutralizing antibody responses also appeared to be relatively similar in HVTN 097 compared to RV144 vaccine recipients; although a direct comparison cannot be made as there were differences in the assay techniques (Figure 4-14).

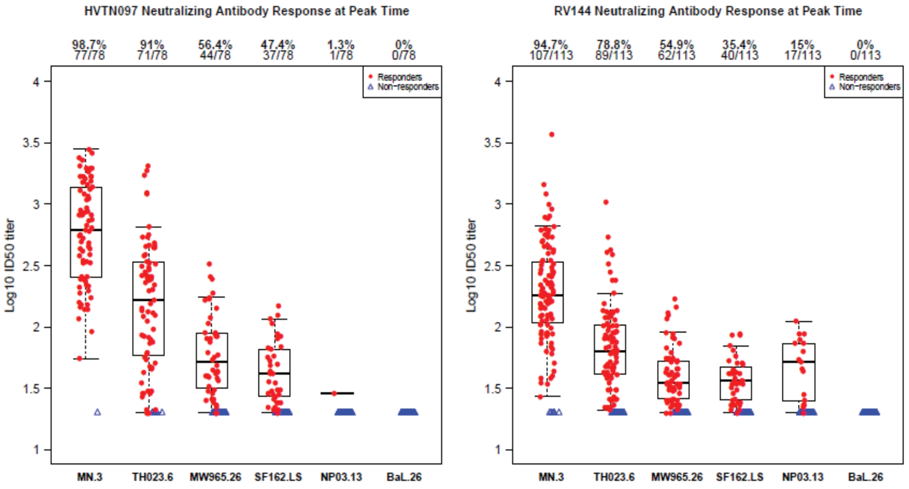

Figure 4-14 Neutralizing antibody responses in HVTN 097 and RV144 vaccinees

4.9.6 HVTN 100

HVTN 100 is a phase 1-2 trial of the ALVAC-HIV (vCP2438) and Bivalent Subtype C gp120/MF59 vaccine regimen proposed for HVTN 702. HVTN 100 is currently being conducted at CRSs in the Republic of South Africa. Evaluation of the safety and immunogenicity of the primary vaccine regimen in HVTN 100 has informed a decision to initiate pivotal efficacy testing in HVTN 702. Immunogenicity criteria for initiating HVTN 702 were selected such that, if the V1V2 IgG binding antibody correlate of risk identified in the RV144 trial is actually a perfect correlate of vaccine protection against HIV-1 infection and if HVTN 100 vaccinees attain a threshold rate and magnitude of these binding antibodies, then similar immune responses in HVTN 702 would predict attainment of a target level of vaccine efficacy in HVTN 702. These criteria included binding antibody response rates and magnitudes to vaccine-matched Env proteins, CD4 response rates to the ALVAC-insert Env, and response rates to vaccine-matched Env V1V2 scaffold proteins (see Section 4.9.6.4).

HVTN 100 enrolled 252 healthy HIV-1–uninfected volunteers aged 18 to 40 years 142 male, 109 female, and one reported other. The first participant enrolled in February 2015 and enrollment was completed in May 2015. The HVTN 100 schema is shown in Table 4-14. All Month 12 injections were completed by 2 June 2016. The HVTN 100 protocol has been amended (Version 3.0, May 2017) to gather additional safety data and to explore the ability of late boost vaccinations administered at month 30 post-enrollment to boost immune responses in HVTN 100 vaccinees. These additional booster injections are expected to start in August 2017 (see Table 4-15).

Table 4-14 HVTN 100 schema

Group	N	Primary vaccine regimen				Booster
		Month 0	Month 1	Month 3	Month 6	Month 12
1	210	ALVAC-HIV (vCP2438)	ALVAC-HIV (vCP2438)	ALVAC-HIV (vCP2438) + Bivalent Subtype C gp120/MF59	ALVAC-HIV (vCP2438) + Bivalent Subtype C gp120/MF59	ALVAC-HIV (vCP2438) + Bivalent Subtype C gp120/MF59
2	42	Placebo	Placebo	Placebo + Placebo	Placebo + Placebo	Placebo + Placebo
Total	252					

Table 4-15 Schema for HVTN 100 Part B

			Booster vaccination
Part A Group	Part B Group[†]	N	Month 30
1	1a	~30	ALVAC-HIV (vCP2438) + Bivalent Subtype C gp120/MF59
	1b	~30	Placebo + Bivalent Subtype C gp120/MF59
2	2	~12	Placebo + Placebo
	Total	72	

[†] Vaccine recipients in Part A (Group 1) are randomized to Group 1a or 1b. Part A placebo recipients (Group 2) continue to receive placebo injections in Part B.

4.9.6.1 Interim summary of blinded safety and tolerability data

Blinded safety data reported as of November 2016 are summarized here. A total of 1198 ALVAC or placebo injections were given to participants in the left deltoid and 703 Bivalent Subtype C gp120/MF59 or placebo injections were given to participants in the right deltoid.

The vaccine regimen is very well tolerated thus far with the vast majority experiencing no or mild local reactogenicity symptoms. Mild pain and/or tenderness was reported by 58% of participants for injections in the left deltoid and by 48% for injections in the right deltoid. Moderate pain and/or tenderness was reported by 18% of participants for injections in the left deltoid and by 11% for injections in the right deltoid. Severe pain and/or tenderness was reported from a left deltoid injection by 1 participant (following the 1st injection) and for a right deltoid injection by 2 participants (following the 3rd or 5th injection).

For the left deltoid, grade 1 (mild) erythema and/or induration injection site reactions were reported by 6% of participants and grade 2 (moderate) reactions by 5% of participants. For the right deltoid, grade 1 erythema and/or induration injection site reactions were reported by 4% of participants and grade 2 reactions by 2% of participants. Three participants have reported erythema and/or induration reactions meeting grade 3 criteria (severe) based on size (≥ 10cm diameter or ≥ 100cm^2 surface area) with no complications (such as ulceration, secondary infection, phlebitis, sterile abscess, or drainage). One person reported a grade 3 right deltoid erythema reaction (> 100 cm^2) occurring on Day 2 of the 4th injection timepoint, which resolved within 4 days. This participant also self-reported severe induration on Day 3, which resolved within 1 day. The participant returned to clinic on Day 4 for examination, and clinic staff observed severe erythema alone without induration or swelling. Antibiotics, analgesics, and antihistamines were prescribed and the participant was discontinued from further vaccinations but continued in follow-up. Another person reported grade 3 induration and erythema in the right deltoid on Day 3 after the 5th vaccination timepoint, resolving by Day 5. Antibiotics, anti-inflammatory and analgesic medications were prescribed and were taken for 3 days. Another person reported severe erythema and induration reactions in the left deltoid occurring on Day 0 after the 5th vaccination timepoint and resolving by Day 3. Antihistamine, oral steroid and analgesic/anti-inflammatory medications were taken. In all 3 participants, the needle used for injection was < 1.5” long, consistent with weight-based guidance for needle length choice provided to sites in the SSPs (112, 113).

Systemic reactions have been reported in 69% of participants thus far, with the vast majority of those reactions being mild in intensity. Malaise and/or fatigue, headache, myalgia and arthralgia appear to be the most common reactions, occurring in 42%, 41%, 37% and 30% of participants, respectively thus far. Other systemic reactogenicity symptoms have included nausea (15%), chills (10%), fever (8%), and vomiting (4%). Maximum severity of systemic symptoms of moderate intensity has been reported in a total of 19% of participants; 7% reported systemic symptoms of moderate intensity after the 1st injection, 3% after

the 2nd, 5% after the 3rd, 2% after the 4th and 5% after the 5th. Severe systemic symptoms have occurred in 2% of participants (4 participants): 2 participants with severe arthralgia occurring after the 1st injection, 1 person with severe malaise and/or fatigue and 1 with severe headache, each after the 5th injection.

4.9.6.2 Interim summary of Adverse Events (AEs) and Serious Adverse Events (SAEs)

As of November 2016, 467 adverse events (AEs) have occurred in 181 participants (72% of participants), of which 282 AEs (60%) were mild, 165 were moderate, (35%), 13 (3%) were classified as severe, 3 (0.6%) were classified as potentially life-threatening, and 2 (0.4%) were fatal. AEs were reported by participants most frequently in the Systems Organ Class (SOC) Infections and infestations (132 participants [52% of enrolled participants]), followed by the SOC Investigations (71 participants [28%]). Fourteen AEs occurring in 11 participants have been assessed by the site investigator as being related to study product; 11 were mild, 3 were moderate and none were severe. These include injection site pruritus in 3 individuals (mild in 2, moderate in 1), lymphadenopathy in 2 individuals (both mild), abdominal pain (moderate), generalized pruritus (moderate), and mild events of diarrhea, injection site nodule, gastritis, dizziness, headache, neutrophil count decreased, and oral paresthesia in 1 individual each.

Eight SAEs, including 2 deaths, have occurred in 6 participants during the trial, all unrelated to study product. One participant experienced 3 separate SAE events resulting from 3 separate assault attacks: severe soft tissue injuries due to assault, potentially life-threatening subdural hematoma, and then multiple injuries to the head and chest that were fatal. SAEs in other participants were: gastrointestinal infection, bi-polar mood disorder, acute rheumatic fever, transient ischemic attack, and completed suicide.

4.9.6.3 Discontinued vaccinations and early terminations

As of 21 November 2016, 232 participants have terminated the study: 207 at scheduled study exit (on-time) and 25 participants have terminated the study prematurely. Twenty-two participants have discontinued vaccinations. Clinical events leading to early terminations and discontinuation of vaccinations (DOV) occurred in 6 participants: death from multiple injuries (unrelated to study product); death from completed suicide (unrelated to study product); severe local reactogenicity (DOV); hypertension (DOV for AE unrelated to study product); mild vomiting occurring on the day of 1st vaccination only (participant declined further study participation); psychiatric diagnosis (DOV and early termination for investigator discretion). Reasons for DOV or early termination in other participants included HIV infection, participant refusal, unable to contact, unable to schedule within visit windows, unable to adhere to study schedule, relocation, desiring to fall pregnant, desiring to donate eggs, unwilling to use contraception, and pregnancy. Two pregnancies have been reported to date, with one participant

reporting a full term live birth and the other pregnant participant is continuing in follow-up.

4.9.6.4 Summary of interim immunogenicity data from HVTN 100

Section 6.1.2 of version 1.0 of HVTN 100 (Part A) describes the immunological criteria guiding the decision whether to advance development of the ALVAC-HIV (vCP2438), Bivalent Subtype C gp120/MF59® regimen. Except for the increased stringency of the V1V2 response rate criteria (LL of the 95% CI increased from ≥ 45% to ≥ 56%), these same criteria ultimately formed the basis of the decision to proceed with HVTN 702, A *pivotal phase 2b/3 multi-site, randomized, double-blind, placebo-controlled clinical trial to evaluate the safety and efficacy of ALVAC-HIV (vCP2438) and Bivalent Subtype C gp120/MF59 in preventing HIV-1 infection in adults in South Africa* (Table 4-16). Immunogenicity data from samples collected from HVTN 100 participants at the month 6.5 timepoint (2 weeks post 6 month vaccination) were compared to data from a new, randomly selected subset of stored samples from RV144 vaccine recipients who were HIV-1 uninfected upon completion of follow-up (the RV144 "comparator arm"). Samples from the RV144 comparator arm and HVTN 100 participants were analyzed contemporaneously using qualified assays in the same laboratories (along with placebo samples for blinding).

All four immunogenicity Go criteria were met.

Table 4-16 Go/No-Go criteria for advancement of the HVTN 100 vaccine regimen to efficacy testing

Variable Measured at Month 6.5	Rationale	Go Criteria Threshold (LL of 95% CI)
1. Env Ab Response Rate (≥ 2 of 3 antigens)	Adequate Ab take to vaccine Env	≥75%
2. Env Ab Magnitude(≥ 2 of 3 antigens)	*Non-inferior* Ab magnitude vs. RV144	GM ratio (new/RV144) ≥50%*
3. Env CD4 Response Rate (1 of 1 antigen)	*Non-inferior* CD4 T-cell take vs. RV144	Difference within 30%*
4. Env V1V2 Response Rate (≥ 1 of 3 antigens)	Adequate to predict achieving estimated VE=50% for 2 years if V1V2 Ab is a predictive immune correlate	≥ 56%

*Non-inferior to RV144 response based on contemporaneous assessment of clade C vaccine samples vs. RV144 vaccinee samples by the same lab.

Binding antibody responses to Env (criteria 1 and 2):

At the month 6.5 timepoint, 100% of vaccinees in HVTN 100 part A developed binding antibodies to the gp120 Clade C strain Env antigens in the ALVAC

vector, as well as the two Clade C strains in the bivalent gp120 protein boost (Figure 4-15). Antibody magnitude values measured by geometric mean titers were 3.6-8.8 fold greater than IgG binding antibody to vaccine-matched responses to Env antigens included in RV144 (Table 4-17). Hence criteria 1 and 2 were met.

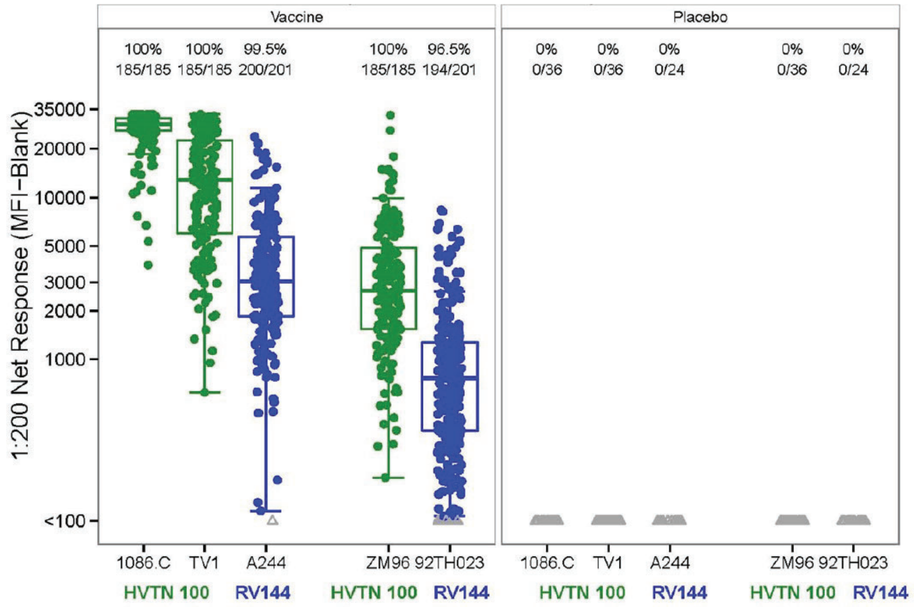

Figure 4-15 Box plots of the binding antibody titers to the vaccine antigens used in RV144 and HVTN 100. The midline of the box plot indicates the median, the ends of the box indicate the 25th and 75th percentiles. Closed dots represent positive responses, open triangles represent negative responses.

Table 4-17 HVTN 100 vs. RV144 Peak bAb magnitudes to gp120 among vaccinees

Protocol	Antigen	n	Geometric Mean Titer	GMR (100/RV144)	GMR 95% CI
**100**	1086	185	26,257.5	8.8	(7.64, 10.2)
**RV144**	A244	201	2,968.9		
**100**	TV1	185	10,726.7	3.6	(3.01, 4.34)
**RV144**	A244	201	2,968.9		
**100**	ZM96	185	2,685.4	3.6	(2.95, 4.39)
**RV144**	92TH023	201	746.1		

CD4 responses (criterion 3)

The CD4+ T cell response rate to vaccine-matched Env sequences in ALVAC (vCP2438), (Env peptide pool ZM96) in HVTN 100 participants was 58%. This was compared to the CD4+ T cell response rate to vaccine-matched Env sequences in ALVAC (vCP1521), (Env peptide pool 92TH023) in RV144 participants of 41%. The response rate difference (100-RV144) was 16% (95% CI: 6, 26%; p = 0.0019). This exceeded the LL response rate difference of -30% and meeting criterion #3.

V1V2 antibody response rates (criterion 4)

The binding antibody response rate to the vaccine-matched 1086.C V1V2 antigen was 71% (95% CI 64% - 77%), thereby meeting criterion #4 that the prevalence of IgG antibodies to the Clade C V1V2 loop in at least one vaccine antigen must be at a lower limit threshold of > 56% (which equates to a response rate of 63%) (Table 4-16). The cumulative V1V2 response is 80%; well above the 63% threshold that was established for modeling a 50% efficacy if V1V2 was the sole Correlate of Protection.

Table 4-18 HVTN 100 Binding Antibody Env V1V2 Response Rates, Per-Protocol Cohort (Go/No-Go Criterion 4)

Antigen	Treatment	Response Rate	95% CI	LL of CI ≥56%	Criterion 4 Passed?
1086.C.V1V2 tags	T1 (n=183)<br>P2 (n=35)	71%<br>0%	(64%, 77%)<br>(0%, 10%)	Yes	Yes
gp70-V1V2CladeB CaseA2	T1 (n=183)<br>P2 (n=35)	50%<br>0 %	(43%, 57%)<br>(0.0%, 9.9%)	No	
gp70-V1V2.TV1	T1 (n=183)<br>P2 (n=35)	62%<br>0%	(55%, 69%)<br>(0%, 10%)	No	
1086.C.V1V2 tags or gp70-V1V2.TV1	T1 (n=183)	80%	(74%, 85%)	N/A	
1086.C.V1V2 tags or gp70-V1V2CladeB CaseA2 or gp70-V1V2.TV1	T1 (n=183)	80%	(74%, 85%)	N/A	

Based on the immunogenicity results of the month 6.5 timepoint in HVTN 100, the decision was made to proceed with HVTN 702.

4.10 Potential risks of study products and administration

Table 4-19 includes general risks of vaccine administration along with risks known from prior clinical studies of ALVAC-HIV products and of envelope protein vaccines adjuvanted with MF59.

Table 4-19 Summary of potential risks of study products and administration

Common (> 10%)	Mild to moderate injection site pain, tenderness, erythema, or swelling/induration/edema<br>Malaise/fatigue, myalgia, arthralgia, nausea/vomiting, lymphadenopathy, asthenia, fever, or headache in the first few days following injection<br>Arm movement limitation<br>A vaccine-induced positive HIV antibody test result
Less common (1% to 10%)	Severe injection site pain or tenderness<br>Chills, flu-like syndrome, diarrhea, rash, or dizziness in the first few days following injection<br>Vasovagal reaction/lightheadedness/dizziness related to the injection procedure<br>Transient changes in clinical laboratory values<br>Injection site hematoma, bruising/ecchymosis, other transient lesions, or bleeding related to the injection procedure
Uncommon (< 1%) or rare (< 0.1%)	Severe localized injection site reaction, such as > 10 cm diameter erythema or induration, sterile abscess or secondary bacterial infection<br>Allergic reaction, including rash, urticaria, angioedema, eyelid swelling, bronchospasm, or anaphylaxis<br>Injection site pruritus, warmth or other non-specific injection site reaction<br>Generalized pruritus<br>Oral paresthesia<br>Syncope, insomnia<br>Abdominal pain, anorexia, gastritis, dysgeusia<br>Skin disorder, acne<br>Muscle damage at the injection site
Theoretical risks	Autoimmune disease<br>Effects on a participant's response to an approved HIV vaccine administered in the future<br>Effects on susceptibility to HIV, if the participant is exposed to HIV<br>Effects on the course of HIV infection/disease, if the participant is infected with HIV<br>Effects on the fetus and on pregnancy

5 Objectives and endpoints

5.1 Primary objectives and endpoints

Primary objective 1:

To evaluate the vaccine efficacy (VE) of ALVAC-HIV (vCP2438) + Bivalent Subtype C gp120/MF59 for the prevention of HIV infection in HIV-seronegative South African adults over 24 months from enrollment

Per the 23 January 2020 DSMB finding that monitoring boundaries for non-efficacy have been met, Primary objective 1 has been superseded by Primary objective 3 below.

Primary endpoint 1:

HIV-1 infection diagnosed after enrollment (concurrent with first vaccination) through 24 months after enrollment

The main vaccine efficacy endpoint is diagnosis of HIV-1 infection during the follow-up of the trial. The occurrence of HIV-1 infection will be detected through HIV-1 tests administered at specified timepoints. Participants found to be HIV-1–infected will have additional testing to confirm the diagnosis of HIV-1 infection (Section 10.3). The vaccine-induced immune responses may lead to reactive HIV-1 tests and difficulty in interpretation. Therefore, the study will utilize blinded Endpoint Adjudicator(s) to review all data that support diagnoses of HIV-1 infection.

Primary objective 2:

To evaluate the safety and tolerability of ALVAC-HIV (vCP2438) + Bivalent Subtype C gp120/MF59 in adults in South Africa

Primary endpoints 2:

Number and frequency of local and systemic reactogenicity signs and symptoms (pain, tenderness, maximum severity of pain and/or tenderness, erythema, induration, fever, malaise/fatigue, myalgia, headache, nausea, vomiting, chills, arthralgia) occurring within 3 days after each vaccine/placebo dose

AEs by body system, Medical Dictionary for Regulatory Activities (MedDRA) Preferred Term, severity, and assessed relationship to study products within 30 days after each vaccine/placebo dose

SAEs, AEs of special interest (AESIs), and new chronic medical conditions (defined as a new onset or exacerbation of medical condition requiring 2 or more

visits to a medical provider during a period of at least 30 days) occurring at any time throughout the study

AEs leading to early participant withdrawal or early discontinuation of study product(s) administration throughout the study

Primary objective 3:

To evaluate the effect of ALVAC-HIV (vCP2438) +Bivalent Subtype C gp120/MF59 vaccination on HIV acquisition in HIV-seronegative African adults

Primary endpoint 3:

HIV infections diagnosed following enrollment and throughout all participant follow-up

5.2 Secondary objectives and endpoints

Secondary objective 1:

To evaluate durability of vaccine efficacy from enrollment through 36 months if Stage 2 occurs

Secondary endpoint 1:

HIV-1 infection diagnosed after enrollment through 36 months post enrollment

Secondary objective 2:

To evaluate vaccine efficacy from Month 6.5 (Week 26) through 24 months post enrollment

Secondary endpoint 2:

HIV-1 infection diagnosed after Month 6.5 through 24 months post enrollment

Secondary objective 3:

To evaluate the immunogenicity of the vaccine regimen

Secondary endpoint 3:

Immune responses at Month 6.5 (week 26 visit) from assays based on the HVTN Laboratory Center assay portfolio (available at http://www.hvtn.org/en/science.html/) such as vaccine-specific binding antibodies and T cell responses.

Secondary objective 4:

To evaluate immunogenicity and immune response biomarkers among vaccine recipients at Month 6.5 (week 26 visit) and Month 12.5 (week 54 visit) as correlates of risk of subsequent HIV acquisition between Month 6.5 and Month 24 and between Month 12.5 and Month 24.

Secondary endpoint 4:

Immune responses from assays based on the HVTN Laboratory Center assay portfolio (available at http://www.hvtn.org/en/science.html/) and/or more assays down-selected from a larger pool of pilot studies, in HIV-1–infected vaccine cases and HIV-1–uninfected vaccine controls

Secondary objective 5:

To evaluate VE by various demographic characteristics

Secondary endpoint 5:

HIV-1 infection diagnosed after enrollment through 24 months, and through 36 months if Stage 2 occurs, by demographic characteristics

Secondary objective 6:

If significant positive evidence of vaccine efficacy from enrollment through 24 months is seen, to assess if and how vaccine efficacy depends on genotypic characteristics of HIV such as signature mutations

Secondary endpoint 6:

HIV-1 infection diagnosed after enrollment through Month 24 and genotypic characteristics of viral sequences from HIV-1–infected participants at HIV-1 diagnosis, such as signature site mutations

Secondary objective 7:

To evaluate and compare genomic sequences of viral isolates from HIV-1–infected vaccine and placebo recipients, and use sieve analysis methods to assess whether there is evidence of vaccine-induced immune pressure on the viral sequences

Secondary endpoint 7:

Viral sequences from HIV-1–infected participants at HIV-1 diagnosis

5.3 Exploratory objectives

Exploratory objective 1:

To evaluate vaccine effects ("vaccine activity") on virologic and immunologic outcomes (eg, HIV-1 viral load (VL) and postdiagnosis CD4+ T-cell count) among HIV-1–infected participants for 6 months post diagnosis, irrespective of ARV use

Exploratory objective 2:

To further evaluate the immunogenicity of the vaccine regimen, additional immunogenicity assays may be performed, and assays may be performed on samples from other timepoints, based on the HVTN Laboratory Center assay portfolio.

Exploratory objective 3:

To evaluate the role of host genetic factors in the immune response to the vaccine regimen and in vaccine effects on study endpoints

Exploratory objective 4:

To understand changes in risk behavior and the potential for risk compensation for all study participants

Exploratory objective 5:

To assess use of biomedical interventions and biological and behavioral factors in the study cohort and how they affect HIV acquisition rates

Exploratory objective 6:

To assess whether the diversity of gut microbiome correlates with vaccine responses and/or HIV infection risk using optionally provided stool specimens.

Exploratory objective 7:

To perform comparative analyses of correlates of risk identified in HVTN 702 and those identified in other HIV vaccine efficacy studies

Exploratory objective 8:

To conduct analyses related to furthering the understanding of HIV, immunology, vaccines, and clinical trial conduct

6 Statistical considerations

6.1 Definitions of study cohorts for analysis

We define 6 study cohorts that are analyzed for addressing various study objectives. This terminology is used throughout the protocol and statistical analysis plan (SAP).

1. Safety Cohort: Randomized participants who receive at least one study injection of vaccine or placebo
2. Modified Intent-to-Treat (MITT) Cohort: Participants in the Safety Cohort who are HIV-1 negative on the date of first injection (Day 0)
3. Week 26 At-Risk Cohort: Participants in the MITT Cohort who have an HIV-1 negative test result at or after the 2 weeks post fourth immunization visit (Week 26 visit)
4. Per-Protocol (PP) Cohort: Participants in the Week 26 At-Risk Cohort who receive all planned immunizations at the first four immunization visits within specified visit windows
5. Immunogenicity Cohort (IC): Participants in the Week 26 At-Risk Cohort who are selected for measurement of immune response endpoints at the primary immunogenicity timepoint (Week 26)
6. Full Immunization Cohort (FIC): Participants in the MITT Cohort who have an HIV-1 negative test result at the 2 weeks after the fifth immunization visit and who receive all planned immunizations within specified visit windows

The MITT Cohort and the Safety Cohort are very similar but not identical to a full Intention-to-Treat Cohort (ie, all randomized participants); the Safety Cohort differs by excluding randomized volunteers who do not enroll; and the MITT Cohort is the subset of the Safety Cohort that also excludes randomized participants discovered later to be HIV-positive by Day 0. Because of blinding and the brief length of time between randomization and enrollment—typically no more than 4 working days—we expect almost all randomized volunteers to be in the Safety Cohort. Given that eligibility for the study requires recent evidence of being HIV-1 uninfected (within 30 days prior to enrollment), we expect almost all enrolled participants to also be in the MITT Cohort.

The primary analyses of safety objectives will be based on the Safety Cohort, whereas the primary analyses of the vaccine efficacy objectives, and the secondary analyses of viral sequence data, will be based on the MITT Cohort. Secondary analyses of the vaccine efficacy objectives will be based on the Week 26 At-Risk Cohort and the Per-Protocol Cohort, in addition to the MITT cohort. Secondary analyses of vaccine immunogenicity and of immune correlates of risk

and protection will be based on participants in both the Immunogenicity and Week 26 At-Risk Cohorts, with additional supportive secondary analyses conducted based on the intersections of the Immunogenicity and, respectively, Per-Protocol or FIC Cohorts. Immunogenicity and immune correlates assessments focus on the Week 26 At-Risk Cohort so as to ensure that immune responses at the primary immunogenicity timepoint (Week 26 visit) are measured in participants who are HIV-1 uninfected at the time of sampling.

The safety analysis will be done according to the treatment received ("as treated"), where participants with AEs or SAEs will be counted as "vaccinated" if they received at least one injection. The efficacy and immunogenicity analysis will be done according to the treatment randomly assigned ("as randomized"), except for analyses of the per-protocol and FIC cohorts which will be as-treated.

In addition, 7 cohorts of participants who are diagnosed with HIV-1 infection during the trial are analyzed for addressing various study objectives. Terminology for these cohorts is defined in Table 6-1, which will be used throughout the protocol and SAP.

Table 6-1 Cohorts of HIV-1–infected study participants

Cohort Name	Definition of Cohort
MITT Infected by 24 Months Cohort	Participants in the MITT Cohort who are diagnosed with HIV-1 infection during the follow-up period after enrollment through the Month 24 visit
MITT Infected by 36 Months Cohort	Participants in the MITT Cohort who are diagnosed with HIV-1 infection during the follow-up period after enrollment through the Month 36 visit
Week 26 At-Risk Infected by 24 Months Cohort	Participants in the MITT Cohort who are diagnosed with HIV-1 infection on or after Week 26 through the Month 24 visit
Week 26 At-Risk Infected by 36 Months Cohort	Participants in the MITT Cohort who are diagnosed with HIV-1 infection on or after Week 26 through the Month 36 visit
Per-protocol Infected by 24 Months Cohort	Participants in the PP Cohort who are also in the Week 26 At-Risk Infected by 24 Months Cohort
Per-protocol Infected by 36 Months Cohort	Participants in the PP Cohort who are also in the Week 26 At-Risk Infected by 36 Months Cohort

6.2 Objectives

The primary, secondary, and exploratory objectives are defined in Section 5.

6.3 Study cohorts utilized for evaluations of study endpoints

Analyses of the HIV-1 infection endpoint and of vaccine activity endpoints will be based on the full MITT Cohort (Section 6.1). Secondary analyses of the vaccine efficacy objectives will be based on the MITT cohort, the Week 26 At-Risk Cohorts, and the Per-Protocol Cohorts.

6.3.1 Primary and secondary endpoints

Primary and secondary endpoints are described in Sections 5.1 and 5.2.

6.4 Accrual and sample size

6.4.1 Sample size calculation for primary endpoint 1 (vaccine efficacy for HIV-1 acquisition)

Recruitment will target a total of 5,400 HIV-uninfected adult participants. Participants will be enrolled over 20 months and randomized with equal probability to the placebo or vaccine regimen with n = 2700 in each group. Participants will receive immunizations at Months 0, 1, 3, 6, 12, and 18 and be followed for HIV infection for a maximum of 18 additional months to the final study visit at Month 36. HIV diagnostic tests will be administered at Month 0 and every 3 months thereafter.

For the comparisons of HIV infection rate between the vaccine and placebo groups in Stage 1, we assume a 4% annual HIV incidence in the placebo group, a 20-month enrollment period with a uniform enrollment rate that is halved in the first 3 months, halved VE in the first 6 months, and a 5% annual dropout incidence. Under these assumptions and the sequential monitoring as described in Section 6.5, n = 2700 per group is selected to ensure approximately 90% power to detect vaccine efficacy from enrollment through 24 months [VE(0-24)] of at least 50% using a 1-sided alpha = 0.025-level log-rank test (versus the null hypothesis of H0: VE(0-24) ≤ 25%). Table 6-2 provides estimates of power to detect varying levels of VE(0-24), under these same assumptions.

Table 6-2 Estimated power to detect different levels of VE(0-24) based on a sample size of n = 2700/group.

True Average VE(0-24)	Power to reject null: VE(0-24) ≤ 25%
30%	7
40%	45
50%	90
60%	100
70%	100
80%	100

To assess the impact of potential emerging prevention modalities on the study power, reports the estimated power to detect VE(0-24) of 50% (versus the null hypothesis of H0: VE(0-24) ≤ 25%) for annual HIV incidence rates in the placebo group ranging from 2.0% to 4.5%, holding the other assumptions and sequential monitoring the same as above. The range of annual HIV incidence rates might reflect various levels of PrEP use. For example, assuming a placebo-group annual incidence of 4% without PrEP, a placebo-group annual incidence of 3% could

arise from one extreme where 25% of the person-years of follow-up in the trial are under PrEP use and PrEP has 100% efficacy, the other extreme where 100% of the person-years of follow-up are under PrEP use and PrEP has 25% efficacy, or something in between such as 31% of the person-years of follow-up are under PrEP use and PrEP has 80% efficacy. Table 6-3 indicates that the study has power above 80% to detect VE(0-24) of 50% or higher (against the null of VE(0-24) ≤ 25%) for incidence as low as 3% annually.

Table 6-3 Estimated power to detect different levels of VE (0-24) based on a sample size of n = 2700/group, for various levels of annual HIV incidence rates in the placebo group. Simulation-based power calculations assume a 20-month enrollment period with a uniform enrollment rate that is halved in the first 3 months, halved VE in the first 6 months, and a 5% annual dropout incidence.

Incidence Rate	Power to reject null: VE(0-24) ≤ 25% when true average VE(0-24) = 50%
2.0%	65%
2.5%	75%
3.0%	81%
3.5%	86%
4.0%	91%
4.5%	93%

To enhance the likelihood that the background incidence assumption is valid and thereby to preserve study power to assess vaccine efficacy, while still maintaining representation of both sexes, no more than 35% and no fewer than 30% males (ie, persons assigned male sex at birth) will be enrolled.

6.4.2 Sample size calculations for primary endpoint 2 (safety for local and systemic reactions)

N = 5400 participants in total provides ample power for assessing safety of the vaccine regimen. If no SAE that is considered related to the study vaccine is observed in the vaccine group (N = 2700), the upper limit of the 95% CI for the true incidence will be 0.14%. Hence, observing no SAE among the vaccinated participants in the vaccine group would provide us with 95% confidence that the true incidence is no more than 0.14%. In addition, as shown in Figure 6-1, sufficient power can be achieved to detect relatively small event rate differences between the vaccine and placebo groups using a 2-sided alpha = 0.05-level Fisher's exact test. For example, if the true safety event rate in the placebo group is 2% (4%), there is 90% power to detect an event rate of 4% (6%) or higher in the vaccine group.

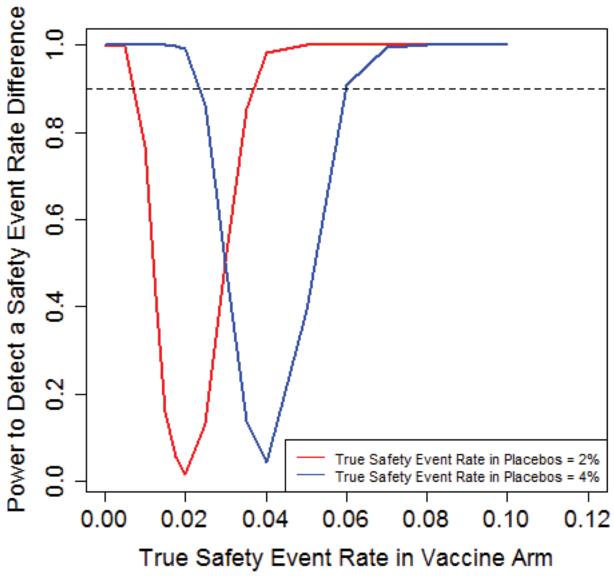

Figure 6-1 Power to detect safety event rate differences between the vaccine (N = 2700) and placebo (N = 2700) arms for assumed true event rates of 2% (red) and 4% (blue) in the placebo arm

6.5 Monitoring of trial

The last DSMB meeting occurred on 23 January 2020 and the interim monitoring is now complete. The following describes the original monitoring plan for the trial.

HVTN 702 will be formally monitored for four types of events that may lead to a modification or termination of the trial. The first three components of the plan monitor vaccine efficacy for potential-harm, non-efficacy/efficacy futility, and high-efficacy (Table 6-4). The potential harm monitoring uses exact binomial tests to compare the rates of vaccine and placebo infections. The non-efficacy and high-efficacy monitoring are based on VE measured by the ratio of cumulative incidences (vaccine vs placebo), with point estimates calculated by the transformation of the Nelson-Aalen estimator and Wald-based confidence intervals.

This section provides an overview of the principles underlying the monitoring approach. The details of the approach and its operating characteristics will be described in the SAP. Of note, all 4 types of monitoring apply to pooled data across both sexes. Sex-specific infection numbers, incidence estimates, and VE estimates will be provided to the DSMB, but no formal sex-specific monitoring guideline will be used due to the smaller number of infections, and hence lower precision of estimation, within each sex.

Table 6-4 Sequential monitoring of vaccine efficacy. CI = confidence interval, LL = lower limit, UL= upper limit. VE(0-24) is vaccine efficacy from enrollment through Month 24; VE(6.5-36) is vaccine efficacy from Month 6.5 (Week 26) through Month 24; VE(0-36) is vaccine efficacy from enrollment through Month 36.

Monitoring Type	Hypotheses	Statistical Method	Timing of Interim Analyses
**Potential Harm**	H0: VE(0-24) ≥ 0%<br>vs.<br>H1: VE(0-24) < 0%	Exact binomial test<br>5% overall Type I error rate	After every infection starting at the 12th until the first non-efficacy analysis
**Non-Efficacy**	H0: VE(0-24) ≥ 40%<br>vs.<br>H1: VE(0-24) < 40%<br>and<br>H0: VE(6.5-24) ≥ 40%<br>vs.<br>H1: VE(6.5-24) < 40%	For both VE(0-24) and VE(6.5-24), LLs of 95% CIs < 0% and ULs of 95% CIs < 40%..	Starting at 59 infections and occurring every 50 infections thereafter, until the end of Stage 1 (up to 7 analyses)
**High Efficacy**	H0: VE(0-36) ≤ 70%<br>vs.<br>H1: VE(0-36) > 70%	95% CI for VE(0-36) lies above 70%	Synchronized with non-efficacy monitoring in Stage 1 contingent on at least 100 participants having reached the Month 36 visit to evaluate VE(0-36), and one analysis halfway through Stage 2

6.5.1 Monitoring for harm

The unblinded statisticians (Section 6.5.8) will "continuously monitor" the trial (ie, examine the data after each confirmed MITT infection endpoint) for early evidence of a potential elevated rate of HIV-1 infection in the vaccine group compared to the placebo group [ie, VE(0-24) < 0%]. The potential harm monitoring follows a classic sequential monitoring framework and utilizes a (nearly) constant boundary on the p-value scale that is determined via controlling the overall type I error rate across tests at 0.05. Such analyses start at the 12th infection (pooled over the vaccine regimen and placebo) and will occur subsequently at each additional infection until the first interim analysis for non-efficacy, after which the non-efficacy analyses serve the function of stopping for potential harm. If the prespecified stopping boundary is reached, then the unblinded statisticians will immediately inform the DSMB. In addition, the DSMB chair will be updated on the accruing unblinded HIV-1 infection data after each confirmed MITT infection. This monitoring guideline is chosen to allow stopping for prudence as early as possible, maximizing participant safety.

Note that the potential-harm monitoring is not intended to reliably establish harm [ie, VE(0-24) < 0%], as a vaccine regimen could meet the boundary and the reported 95% confidence interval for VE(0-24) would include 0%. Rather, the objective is to apply extra caution and prudence for a prevention trial that enrolls healthy volunteers.

6.5.2 Monitoring for non-efficacy/efficacy futility

The DSMB will monitor the vaccine for non-efficacy/efficacy futility, defined as evidence that it is highly unlikely that the vaccine has a beneficial effect on acquisition of VE(0-24) of 40% or more. Such analyses will start at 59 infections, which is chosen as the first infection total when a 95% confidence interval about

an estimated VE(0-24) = 0% (based on a Cox proportional hazards model) would lie below 40%. Analyses will take place in increments of 50 infections until the end of Stage 1.The criterion for non-efficacy is that, for both VE(0-24) and VE(6.5-24), the lower limit of the 95% confidence interval lies below 0% and the upper limit lies below 40%. By checking both VE(0-24) and VE(6.5-24) confidence intervals, the monitoring plan is designed to protect against stopping prematurely based on ramping vaccine efficacy over the intercurrent period of 0-6 months. For each parameter, VE is estimated 2 ways. First, defining VE as 1 minus the ratio of cumulative HIV incidence estimates, vaccine vs. placebo, VE is estimated by the transformation of the Nelson-Aalen estimator for the cumulative hazard function. Second, VE measured by 1 minus the hazard ratio (HR) for HIV infection is estimated using Cox proportional hazards regression. Confidence intervals for both VE estimates must satisfy the non-efficacy criterion for both VE(0-24) and VE(6.5-24). The rationale for using both cumulative-incidence-based and proportional-hazards-based VE estimates is that: 1) the former better protects against concluding non-efficacy due to ramping vaccine efficacy but the latter is more stable at early interim analyses; and 2) proportional-hazards-based VE estimates are used to determine when to start the interim monitoring and are universally used in vaccine efficacy studies. When there are sufficient numbers of infections in each sex at birth to ensure stable confidence interval estimates, sex-stratified VE estimates based on both approaches will be provided as described in Section 6.9.5.1.

6.5.3 Monitoring for high efficacy

Monitoring of high efficacy allows early detection of a highly protective vaccine if there is evidence that vaccine efficacy from enrollment through 36 months [VE(0-36)] is above 70%. Analyses will be harmonized with those for non-efficacy monitoring, with the exception that the high efficacy analyses will only start once at least 100 participants have reached the terminal Month 36 visit. This condition ensures that sufficient follow-up has accumulated to estimate VE(0-36). If Stage 2 occurs, there will be one final high efficacy interim analysis that occurs 6 months into Stage 2. The criterion for high efficacy is that the 95% confidence interval for VE(0-36) lies above 70%. Here again, VE is estimated using a ratio of cumulative incidences and using Cox proportional hazards regression, and the high efficacy criterion must be satisfied by both estimators. Note that, whereas the monitoring for potential-harm and non-efficacy restricts to infections diagnosed between Months 0 and 24, the monitoring for high-efficacy counts all infections during Months 0-36, given that early-stopping for high-efficacy would only be warranted under some evidence for durability of vaccine efficacy.

6.5.4 Definition of the end of Stage 1 and criterion for continuing to Stage 2

Whereas the timing of interim analyses is event-driven, the total number of Stage 1 infections is not fixed. The end of Stage 1 is when the last enrolled participant reaches the Month 24 visit. Ensuring all participants are followed to Month 24 maximizes power and precision for assessing VE(0-24).

Early stopping of the vaccine regimen will be recommended if one of the potential harm or non-efficacy boundaries is met at a prespecified analysis time, in which case a decision will be made regarding whether to follow participants to Month 24 (Stage 2 will not occur). If the high-efficacy boundary is met at a planned interim analysis, the vaccine regimen will be declared highly efficacious and placebo recipients may be offered the vaccine even while follow-up continues through Stage 1 (or Stage 2, if applicable). If none of the stopping boundaries are reached by the end of Stage 1, vaccine efficacy from enrollment through Month 24 [VE(0-24)] is evaluated at the end of Stage 1. VE is defined as 1-cumulative incidence ratio (vaccine vs. placebo) and is estimated by the transformation of the Nelson-Aalen estimator for the cumulative hazard function. If the lower bound of the 95% confidence interval for VE(0-24) is > 0% at the end of Stage 1 (equivalently, the 1-sided p-value for testing H0: VE(0-24) ≤ 0% is above 0.025), trial participants will continue blinded follow-up through Stage 2. On the other hand, if at the Stage 1 efficacy analysis the lower bound of the 95% confidence interval for VE(0-24) ≤ 0%, Stage 2 will not occur even if none of the stopping boundaries were reached by the end of Stage 1. This corresponds to continuing to Stage 2 if, at the end of Stage 1, the estimated VE(0-24) is approximately 19-21% or greater; the range of VE values is due to uncertainty in the total number of Stage 1 infections.

6.5.5 Monitoring for operational futility

The DSMB monitors the trial for operational futility, defined as overly slow progress toward full enrollment or sufficient numbers of MITT infections to support the primary analysis, and by other measures of underperformance. Approximately semi-annual DSMB meetings will be held for monitoring operational futility during the closed session, synchronized with interim analyses of vaccine efficacy. At the time of each semi-annual DSMB meeting, the HVTN 702 Oversight Group (OG) will receive the components of the operational futility analysis that are based on blinded data (pooled across treatment arms), as described below.

The first component of the operational futility monitoring plan includes projections of the time until enrollment completes. Under design assumptions, enrollment is expected to take 20 months. The projection of this time, including an assessment of its probability distribution, will be presented to the DSMB, and compared with the pre-trial assumptions.

The second component of the operational futility monitoring plan includes projections of the probability distribution of the treatment-arm-pooled infection total, and the arm-specific infection totals, over 0-24 months. These distributions will be calculated under 3 scenarios: 1) using the observed treatment-arm-pooled infection rate to-date and assuming that VE(0-24) = 25% [the null hypothesis]; 2) using the observed treatment-arm-pooled infection rate to-date and assuming VE(0-24) = 50% [the alternative hypothesis]; and 3) using the observed by-arm infection rates observed to date. Methods for performing these calculations will be specified in the SAP.

A report based on blinded data will be shared with the study OG. It will only contain projections of the treatment-arm-pooled infection total (not the arm-specific totals), and only projections under scenarios 1 and 2, since scenario 3 contains information about the observed VE to date.

The probability distribution of the Stage 1 (0-24 month) treatment-arm-pooled infection total, under design assumptions on enrollment, dropout, and infections, is shown in Table 6-5.

Table 6-5 Distribution of Stage 1 infections (pooled over treatment arms) ignoring interim monitoring

True VE	Percentiles of the distribution of the number of Stage 1 infections														
(0-24)	2.5%	5%	10%	20%	25%	30%	40%	50%	60%	70%	75%	80%	90%	95%	97.5%
**0%**	357	362	369	377	380	383	388	393	397	403	405	409	417	424	431
**50%**	265	271	277	284	286	289	293	297	301	306	308	311	319	325	330

The estimated reverse cumulative distribution function (rcdf) of the pooled number of HIV infection endpoints will be shown, for each of the 3 scenarios above, for a range of endpoint counts on the x-axis. The rcdf plot will include an additional axis on the top, showing the approximate power under the proportional hazards assumption of a one-sided test with significance level 0.025 to reject H_0: VE(0-24) $\leq$ 25% assuming VE(0-24) = 50% throughout the trial, for the range of endpoint counts on the bottom x-axis. While the rcdf of the pooled number of endpoints conditions on the observed data to date, the power approximation follows an analytical formula (calculated by solving equation (1) in (114)) and disregards observed data to date to maintain trial integrity. Rcdfs will also be used to summarize the distribution of the number of HIV infection endpoints by arm.

In addition, information on use of antiretroviral drugs that could affect accrual of endpoint infections will be provided to the DSMB as available. If the HIV incidence estimates are below projected despite good retention and adherence, the DSMB may elect to engage with the protocol team and OG as to what corrective actions can be undertaken, including expanding enrollment, and using specific demographic or behavioral risk factors to enroll higher risk participants or those coming from high epidemic communities.

If the DSMB does recommend termination and the trial is stopped, then the final analysis will be performed and the results made public.

If at any time operational futility guidelines are met and yet it appears that value exists in continuing the trial, the statisticians will provide the DSMB with additional information, as appropriate, for use in their consideration of whether to recommend trial termination.

6.5.6 Operational analysis near the end of enrollment to decide whether to consider expanding enrollment

As mentioned above, the HVTN 702 OGC will receive the results of the components of the operational futility analysis that are based on blinded data. The committee will also be provided additional information at the time of a special DSMB meeting scheduled to take place approximately 3 months before the completion of enrollment—based on ongoing projections of time until full enrollment by study statisticians. For this meeting, study statisticians will prepare a report that includes the calculations typically in an operational futility analysis, along with additional guidance as to whether the HIV incidence – pooled over treatment arms—is “too low” or “acceptable” to sufficiently power the study. (The details of this guidance will be described in the study SAP.) The report will be shared both with the DSMB and with the study OG. The OG will then have the opportunity to decide whether, if incidence is lower than anticipated but not so low that operational futility is declared, to expand enrollment beyond the originally planned 5400, in order to maintain the ability of the study to meet the study’s primary objectives. The meeting will be timed before but near to the end of enrollment, to allow a potential decision to expand enrollment to occur before the enrollment apparatus is scaled down, while also allowing the decision to be made on the maximal amount of primary endpoint infections. The OG may also be presented information on the degrees of enrollment expansion that would be required in order to achieve treatment arm-pooled infection totals corresponding to various levels of power to reject the primary null hypothesis if VE(0-24) = 50%, approximated under the proportional hazards assumption.

6.5.7 Performance standards for quality of trial conduct

The protocol team and study investigators will have performance standards regarding the quality of trial conduct in addition to the study event rate. Some of these standards will relate to achievement of targeted levels of: (i) participant enrollment into the trial; (ii) adherence to study interventions; and (iii) retention of participants. In addition to using the above guidelines for operational futility, the DSMB and the HVTN 702 leadership will monitor whether the trial is achieving at least minimally acceptable levels regarding key performance standards.

6.5.8 Roles of study statisticians

HVTN SDMC statisticians will be “blinded” or “unblinded”. During protocol development and after primary follow-up is completed, there will be no distinction between the roles; both types of statisticians will be responsible for designing and analyzing the study. During the primary follow-up period, however, only the unblinded statisticians will see interim data broken down by treatment arm. Their role will be to conduct the interim monitoring and to produce and present reports on accruing data to the study DSMB. During the primary follow-up period, blinded statisticians will see only the interim data pooled across treatment arms. This way, blinded statisticians can assist protocol leadership in

making decisions about modifications to the protocol without being influenced by interim efficacy results.

6.5.9 Power for secondary VE analysis

Secondary Objective 2 evaluates vaccine efficacy against infections diagnosed between the Month 6.5 and Month 24 visits in the per-protocol cohort. Unconditional power to address this objective is summarized in Table 6-6. For example, there is 77% power to detect VE(6.5-24) ≥ 50% (versus the null of H0: VE(6.5-24) ≤ 25%) assuming a 10% probability that a participant misses a vaccination and is therefore not included in the per-protocol cohort.

Table 6-6 Power to detect different levels of VE(6.5-24) based on a sample size of n = 2700 per group.

True Average VE(6.5-24)	Power to reject null: VE(6.5-24) ≤ 25%
30%	6%
40%	31%
50%	75%
60%	97%
70%	100%
80%	100%

Under Secondary Objective 5, vaccine efficacy will be assessed by various demographic characteristics, including gender. Given that the trial will enroll more women than men, and that HIV incidence is expected to be lower in men than in women, calculations were performed to assess the precision with which vaccine efficacy can be assessed among men. Table 6-7 describes the precision with which VE(0-24) can be estimated among men, assuming VE(0-24) = 50% and under different assumed HIV incidence rates among men. Importantly, these confidence intervals rule out VE(0-24) ≤ 0%, as long as HIV incidence among men is 2.0% or more annually and 30% men are enrolled, and as long as HIV incidence among men is 1.5% or more annually, if 35% men are enrolled.

Table 6-7 Expected confidence interval around VE(0-24) = 50% among men, assuming 30% or 35% men, under different assumed HIV incidence rates among men, for a trial with n = 2700 per group

Fraction of Men in study (%)	Assumed Infection Rate for Men (% per year)	Expected Number of Infections (Men)	95% CI for VE (men)
30	1.0	22	(-0.213, 0.794)
30	1.5	34	(-0.02 , 0.755)
30	2.0	45	(0.071, 0.731)
35	1.0	26	(-0.13 , 0.779)
35	1.5	39	(0.027, 0.743)
35	2.0	53	(0.115, 0.718)

6.5.10 Trial duration

In addition to enrollment rates, the duration of the trial will depend on the duration of the vaccine regimen's evaluation and hence the probabilities of reaching each possible trial outcome (potential harm, non-efficacy, positive efficacy but not high efficacy, and high efficacy).

Figure 6-2 shows the distribution of the duration of the vaccine regimen's evaluation over both Stages 1 and 2. The figure shows that, if VE(0-24) = 50% with VE halved in the first 6 months, there is probability of nearly 1 (or 100% chance) that the evaluation of the vaccine regimen will carry all the way through Stage 2 (56 months), which includes 20 months of enrollment plus 36 months of follow-up for all enrolled participants. In addition the median trial duration is 56 months. If VE(0-24) = 0%, then there is a 100% chance that the vaccine regimen's evaluation will be completed in Stage 1, with a median trial duration of 24 months.

The duration of the trial is now determined by when the last enrolled participant reaches the end of follow-up (12 months after the last received vaccination), which is expected to be a maximum of 44 months from study opening.

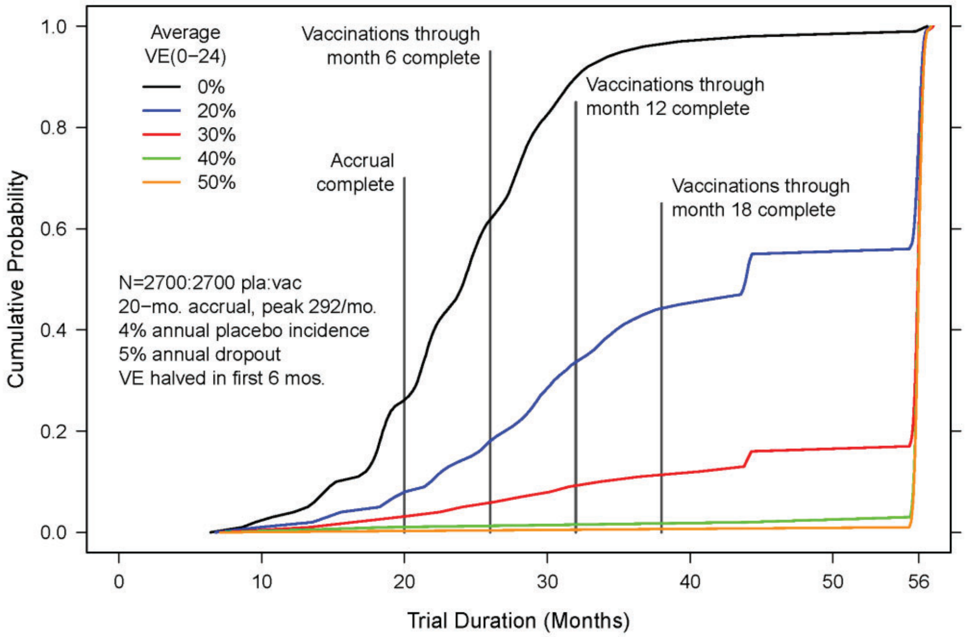

Figure 6-2 Total trial duration for the evaluation of the vaccine regimen over Stages 1 and 2

6.6 Assessment of PrEP use

The use of oral FTC/TDF as PrEP (either off-study or provided in the study) may impact study outcomes (eg, by lowering HIV incidence with a loss of study power). Dried blood spot samples will be collected and stored for assessment of

quantitative concentrations of intracellular TFV (see Section 11.7), using a calendar based selection of visits that will ensure representative sampling of participants across the entire visit schedule at all sites throughout the study. A detailed testing plan, specified in the study monitoring plan, has been developed to prospectively monitor FTC/TDF use during the trial to inform the DSMB, and assess the potential impact of FTC/TDF use on endpoint accrual during the study. Prevalence of PrEP will be reported both as the estimated proportion of participant visits with highly adherent PrEP use (above the concentration of TFV designated as consistent with daily dosing) and the proportion of participant visits with any detectable PrEP use (concentration of TFV above the lower limit of detection for the dried blood spot assay). Additionally, we will use knowledge of pharmacokinetic quantitative levels to estimate the proportion of study person years protected by highly adherent use of FTC/TDF. PrEP use measures will be reported by arm to the DSMB in the closed session; in addition both the OG and the protocol team leadership will see pooled estimates of FTC/TDF use.

6.7 Randomization of treatment assignments

The randomization sequence will be obtained by computer-generated random numbers and provided to each HVTN CRS through the SDMC's Web-based randomization system. The randomization will be stratified by sex at birth and site and done in such a way as to ensure balance across arms within levels jointly determined by these factors. At each institution, the pharmacist with primary responsibility for dispensing study products is charged with maintaining security of the treatment assignments. Participants and site staff will be blinded as to the treatment group assignment throughout both Stage 1 and Stage 2, if it occurs.

6.8 Blinding

Participants, site staff (except for site pharmacists), and the OG will be blinded as to participant treatment group assignments (eg, vaccine or control) throughout both Stage 1 and Stage 2, if it occurs. Study product assignments are accessible to those HVTN CRS pharmacists, DAIDS protocol pharmacists and contract monitors, and SDMC staff who are required to know this information in order to ensure proper trial conduct. Any discussion of study product assignment between pharmacy staff and any other HVTN CRS staff is prohibited. The DSMB members also are unblinded to treatment assignment in order to conduct review of trial safety and efficacy.

When a participant leaves the trial prior to study completion, the participant will be told he or she must wait until all participants have completed follow-up to learn his or her treatment assignment.

Emergency unblinding decisions will be made by the site investigator. If time permits, the HVTN 702 PSRT should be consulted before emergency unblinding occurs.

6.9 Statistical analysis

This section describes the final study analysis, unblinded as to treatment group assignment. With the exceptions noted for safety analyses, all data from enrolled participants will be analyzed according to the initial randomization assignment regardless of how many vaccinations they received. The analysis is a modified intent-to-treat analysis in that individuals who are randomized but not enrolled do not contribute data and hence are excluded. Because of blinding and the brief length of time between randomization and enrollment—typically no more than 4 working days—very few such individuals are expected.

Analyses for primary endpoints will be performed using SAS and R. All other descriptive and inferential statistical analyses will be performed using SAS, StatXact, or R statistical software.

No formal multiple comparison adjustments will be employed for multiple safety endpoints, multiple efficacy endpoints, multiple immunogenicity endpoints, or secondary endpoints. However, multiplicity adjustments will be made for certain immunogenicity assays, as discussed below, when the assay endpoint is viewed as a collection of hypotheses (eg, testing multiple peptide pools to determine a positive response).

6.9.1 Analysis variables and cohorts for analysis

The analysis variables consist of baseline participant characteristics, safety, efficacy, activity, immunogenicity, and for primary- and secondary-objective analyses. Analyses of safety are done in the Safety Cohort, analyses of efficacy against HIV-1 infection are done in the MITT Cohort, analyses of vaccine activity against postinfection endpoints are done in the Infected MITT Cohort, immunogenicity analyses are done in the Week 26 At-Risk Cohort, analyses are done in the MITT Cohort, and HIV genetics analyses are done in the Infected MITT Cohort. The primary efficacy analysis for VE(0-24) will be done in the MITT Cohort. Other estimates of efficacy will be supportive.

6.9.2 Baseline comparability

Treatment arms will be compared for baseline participant characteristics using descriptive statistics.

6.9.3 Safety/tolerability analysis

Since enrollment is concurrent with receiving the first injection, all participants will have received at least 1 vaccination and therefore will provide some safety data. During the course of the trial, unblinded analyses of safety data will be prepared approximately every 6 months for review by the DSMB. Ad hoc safety reports may also be prepared for DSMB review at the request of the HVTN 702 PSRT. The HVTN leadership must approve any other requests for unblinded safety data prior to the end of the scheduled follow-up visits.

6.9.3.1 Reactogenicity

The number and percentage of participants experiencing each type of reactogenicity sign or symptom will be tabulated by severity and treatment group and the percentages displayed graphically by arm. For a given sign or symptom, each participant's reactogenicity will be counted once under the maximum severity for all injection visits. In addition, to the individual types of events, the maximum severity of local pain or tenderness, induration or erythema, and of systemic symptoms will be calculated. Kruskal-Wallis tests will be used to test for differences in severity between arms.

6.9.3.2 AEs and SAEs

AEs will be summarized using MedDRA System Organ Class and preferred terms. Tables will show by treatment group the number and percentage of participants experiencing an AE within a System Organ Class or within preferred term category by severity and by relationship to study product. For the calculations in these tables, a participant with multiple AEs within a category will be counted once under the maximum severity and by causal relationship to study product. Formal statistical testing comparing arms is not planned for all AEs since interpretation of differences must rely heavily upon clinical judgment. Fisher's exact tests will be used to compare rates of AEs deemed related to study products between vaccine and placebo. In addition, several aggregate safety endpoints will be compared across vaccine and placebo arms, as detailed in the study SAP. The false-discovery rate procedure of Mehrotra and Heyse (115) will be applied to compare grouped systemic AEs divided by body systems between treatment arms. Parallel analyses will include all AEs and AEs leading to participant withdrawal or early discontinuation of study product(s).

A listing of SAEs reported to the DAIDS Regulatory Support Center (RSC) Safety Office will provide details of the events including severity, relationship to study product, time between onset and last vaccination, and number of vaccinations received. A separate listing will do the same for AESIs, AEs leading to early participant withdrawal or early discontinuation of study product(s), and AEs of new chronic medical conditions, AESIs for this protocol include but are not limited to potential immune-mediated disorders; a sample list of AESIs is provided in Appendix J.

6.9.3.3 Reasons for vaccination discontinuation and early study termination

The number and percentage of participants who discontinue vaccination and who terminate the study early will be tabulated by reason and treatment arm.

6.9.4 Immunogenicity analysis

Data from quantitative assays will be summarized for the treatment group using crude and net percentages with positive responses, and crude geometric means, for each timepoint for which an assessment is performed. Data from qualitative

(ie, positive or negative) assays will be summarized by tabulating the frequency of positive responses for each assay by group at each timepoint that an assessment is performed.

6.9.5 Vaccine efficacy and activity analyses

6.9.5.1 Primary vaccine efficacy analysis: HIV-1 acquisition

We define the date of diagnosis of HIV-1 infection to be the draw date of the first sample that leads to a positive result by the diagnostic algorithm described in Section 10.3. To evaluate the primary vaccine efficacy endpoint (HIV-1 acquisition), the incidence of acquired HIV-1 infections ("events") in the vaccine group will be compared to the incidence in the placebo group within the first 24 months since first vaccination. The vaccine effect will be assessed using a ratio of cumulative incidences of HIV infection over the first 24 months (vaccine vs. placebo), estimated using the transformed Nelson-Aalen cumulative hazard function estimator, and tested using a Wald test. Cox proportional hazards regression will also be used for estimating VE(0-24), measured by 1 minus the HR (vaccine vs. placebo) and for testing whether the VE(0-24) differs from 25%. Both VE estimators will allow for different HIV incidence by gender. The Cox regression analysis will do this by stratifying on sex at birth, allowing for a different baseline risk of HIV-infection by sex. The cumulative incidence estimation method will estimate VE separately by sex and then combine the sex-specific BE estimates into 1 estimate using the stratified Aalen-Johansen estimator with a single failure type (116). The influence curve-based variance estimator will be used (117).

In addition, to assess potential time-effects of vaccine efficacy, the Kaplan-Meier method will be used to plot the estimated cumulative incidence rates over time for the vaccine and placebo groups. This method will be used to estimate cumulative vaccine efficacy over time, defined as [(1 - ratio (vaccine/placebo) of cumulative incidence by time t) × 100%], with the method of Parzen, Wei, and Ying (118) applied to estimate pointwise and simultaneous 95% CIs.

For evaluating the vaccine effect on HIV-1 acquisition using pre- and post-unblinding data, analyses will adjust for potential confounding. The cumulative-incidence-based VE method will do this by stratification on potential confounders as described above for adjusting for gender. The Cox regression analysis will adjust for potential confounders as covariates. The analyses will include all baseline variables predictive of HIV infection pooled over the vaccine and placebo arms and will include the baseline risk score. If there is evidence of differences in risk behavior over time between treatment arms, a time-dependent version of the behavioral risk score will be included in the Cox model. Depending on data completeness and covariate patterns, the Cox model may also include time-dependent indicators of STIs (gonorrhea, chlamydia, syphilis, and HSV if available). Goodness-of-fit tests will be performed [including the Grambsch and Therneau (119) test based on Schoenfeld residuals] to assess the proportional hazards assumption of the Cox model.

Additive differences in cumulative infection probabilities for the vaccine versus placebo group will also be estimated, and plots used to show estimated additive differences and associated 95% confidence intervals over time The collaborative targeted maximum likelihood estimation method (120) may be also used for inference, which in addition to allowing confounding adjustment can correct for potential bias due to covariate-dependent censoring.

6.9.5.2 Secondary vaccine efficacy analyses: HIV-1 acquisition

Vaccine efficacy absent PrEP

The primary analysis will be repeated, where only MITT infections in participants who were not using prophylactic ARVs at the time of HIV-1 diagnosis or first evidence of infection will be included in the analysis. Plasma drug levels will be used to determine eligibility for this analysis. A participant is eligible if the plasma drug levels are undetectable at the diagnosis visit and at the visit with earliest evidence of HIV-1 infection (if different from the diagnosis visit). Since the ARVs are only detectable in plasma through roughly 14 days (121) and some participants may have become infected before the 14-day window, with this approach we are not assured that all those invluded in the analysis were not using prophylactic ARVs at the time of infection. Therefore, an additional analysis will address this issue by also excluding participants from the HIV-1 acquisition analysis if they self-reported drug use in the last 30 days at either the diagnosis visit or the last visit prior to diagnosis. This additional analysis will evaluate uninfected participants without accounting for data on their plasma drug levels.

Per-protocol vaccine efficacy

The primary analysis will be repeated in the Per-Protocol Cohort, with the time origin the date of the Week 26 immunization visit; participants becoming HIV infected or dropping out before Week 26 will be excluded from the analysis. In addition, the causal inference method of Gilbert, Shepherd, and Hudgens (122) will be used to assess vaccine efficacy in the subgroup who would be per-protocol under either treatment assignment.

Durability of vaccine efficacy

Vaccine efficacy over all available follow-up times will be assessed using the method of Parzen, Wei, and Ying (118) described above, as well as with nonparametric HR estimation (123) and with Cox proportional hazards modeling with time-dependent covariates. If the analysis suggests time-invariant vaccine efficacy, then the Cox model and the method of Lu and Tsiatis (124) will be used to estimate proportional-hazards VE and to test for VE different from zero accounting for all available follow-up time. The SAP will provide details of these analyses.

Vaccine efficacy by sex at birth

Generalized Wald tests for interaction will be used to test for evidence of differential VE by sex, using cumulative-incidence-based VE estimates. In the context of Cox proportional hazards models, Wald tests for interaction will be used to test for evidence of differential vaccine efficacy by sex at birth.

Vaccine efficacy in a population with equal gender distribution

Due to the small number of infections in men at birth, this analysis will not be performed.

The primary analysis of vaccine efficacy will evaluate efficacy in a population with a gender distribution equal to that in the 702 trial. As a secondary analysis, vaccine efficacy in a population with equal gender distribution will be estimated. The cumulative-incidence-based VE estimator will be used; VE will be estimated separately by sex and then combined using equal weights into one vaccine efficacy estimate.

Vaccine efficacy, taking into account number of founding viruses

A secondary analysis will assess the vaccine effect on HIV-1 acquisition over 0-24 months using the method of Follmann and Huang (125) that incorporates information on the number of HIV-1 founder viruses in HIV-1–infected participants. By incorporating this additional information, the method increases efficiency if the vaccine reduces the number of founders.

6.9.5.3 Secondary vaccine efficacy analysis: HIV-1 acquisition over 36 months

If the vaccine regimen is not stopped at an interim analysis and meets the criteria to proceed to Stage 2, then it will be evaluated for 36 months of follow-up for all participants. Vaccine efficacy as a continuous function of time since entry [VE(t)] will be estimated using a variety of methods including the Cox model with flexible parametric regression coefficients and nonparametric smoothing (123). In addition, the causal inference method of Shepherd et al. (122, 126), as described in the commentary by Hughes and Dai (in (127)), may be used to assess the causal vaccine efficacy over various time-periods in the subgroup that would be uninfected under either treatment assignment up to that time-point.

6.9.5.4 Secondary vaccine efficacy and activity analyses of viral sequences

Vaccine efficacy and genotypic characteristics

A variety of methods including cause-specific Cox model and case-only method (128) may be used to assess genotypic characteristics of HIV as potential effect modifiers of VE(0-24).

Sieve analysis

Acquisition sieve analysis methods including genome scanning tests described in Gilbert et al. (129) and their extensions will be used to evaluate the relationship between VE(0-24), and the genotypic differences between the incoming exposing/founder sequences and the HIV insert sequence(s) represented in the vaccine construct.

6.9.5.5 Secondary vaccine activity analyses of pre-HAART viral load and CD4+ cell counts

Due to the limited pre-HAART viral load and CD4+ cell count data collected, these analyses will primarily be descriptive.

Early pre-HAART VL endpoint

The vaccine effect on the mean early pre-HAART VL endpoint will be assessed, in terms of point and 95% confidence interval estimates of the mean difference (vaccine minus placebo) and a 2-sided p-value for whether the mean difference equals zero. The robust likelihood-based method of Little and An [128] will be used, which provides unbiased inferences if the missing endpoint data due to highly active antiretroviral therapy (HAART) initiation or dropout are missing at random (MAR), and is selected to minimize potential bias in the analysis that could occur due to missing VL endpoint data. The MAR assumption can be stated as follows: Whether an infected participant is missing an early VL endpoint depends only on his or her observed data. The MAR assumption is expected to hold, at least for most infected participants. The reasons for this will be described in the SAP, as will be reasons why Little and An's method (130) is expected to work well. If an analysis suggests a vaccine effect, then sensitivity analysis methods (such as doubly robust methods (131)) will be applied to evaluate the robustness of the vaccine effect, as elaborated in the SAP. The Little and An method may be substituted for a complete-case analysis that compares mean VL/CD4+ endpoints using t-statistics, if the rate of missing endpoint data is low (eg, less than 10% of participants with at least one missing endpoint value, for longitudinal endpoints, or less than 10% of participants missing the endpoint of interest, for univariate endpoints). The Little and An method is also designed to maximize efficiency by leveraging information in auxiliary covariates that predict missing values of pre-HAART VL.

Preseroconversion VL endpoint

The Little and An method will be used for assessing the vaccine effect on the preseroconversion VL endpoint. As will be detailed in the SAP, similar to the analysis of pre-HAART VL endpoint, sensitivity analysis and complete-case analysis may be performed when appropriate.

Longitudinal pre-HAART VL and CD4+ T-cell count endpoints

Early VL and pre-HAART VL and CD4+ T-cell trajectories will be assessed. The method of Little and An will be applied to assess the vaccine effect on the pre-

HAART VL or the pre-HAART CD4+ cell count at early fixed visit timepoints. The SAP will detail additional longitudinal data methods that may be applied to the longitudinal VL and CD4+ T-cell count data.

7 Selection and withdrawal of participants

Participants will be healthy, HIV-uninfected (seronegative) adults who comprehend the purpose of the study and have provided written informed consent. Volunteers will be recruited and screened; those determined to be eligible, based on the inclusion and exclusion criteria, will be enrolled in the study. Final eligibility determination will depend on information available at the time of enrollment, including results of screening laboratory tests, medical history, physical examinations, and answers to self-administered and/or interview questions.

Investigators should always use good clinical judgment in considering a volunteer's overall fitness for trial participation. Some volunteers may not be appropriate for enrollment even if they meet all inclusion/exclusion criteria. Medical, psychiatric, occupational, or other conditions may make evaluation of safety and/or immunogenicity difficult, and some volunteers may be poor candidates for retention.

Determination of eligibility, taking into account all inclusion and exclusion criteria, must be made within 56 days prior to enrollment unless otherwise noted in Sections 7.1 and 7.2.

Consult the HVTN 702 PSRT for questions related to selection and withdrawal of participants that are not specifically addressed below.

7.1 Inclusion criteria

General and Demographic Criteria

1. **Age** of 18 to 35 years

2. **Sexually active**, defined as having had sexual intercourse at least twice in the past 30 days prior to screening, and is considered by the site staff to be at risk for HIV infection.

3. **Access to a participating HVTN CRS** and willingness to be followed for the planned duration of the study

4. Ability and willingness to provide **informed consent**

5. **Assessment of understanding**: volunteer demonstrates understanding of this study prior to first vaccination with verbal demonstration of understanding of all questions.

6. **Agrees not to enroll in another study** of an investigational research agent until the participant is unblinded or their study participation ends, whichever occurs last

7. **Good general health** as shown by medical history, physical exam, and screening laboratory tests

HIV-Related Criteria:

8. Willingness to receive **HIV test results**

9. **Willingness to discuss HIV infection risks** and willing to receive HIV risk reduction counseling

Laboratory Inclusion Values

Chemistry

10. **Alanine aminotransferase (ALT)** < 2.5 times the institutional upper limit of normal

Virology

11. **Negative HIV-1 and -2 blood test within 30 days prior to enrollment**: Sites may use locally available assays that have been approved by HVTN Laboratory Operations.

Reproductive Status

12. **Volunteers who were born female**: negative serum or urine beta human chorionic gonadotropin (β-HCG) pregnancy test performed prior to vaccination on the day of initial vaccination. Persons who are NOT of reproductive potential due to having undergone total hysterectomy or bilateral oophorectomy (verified by medical records), are not required to undergo pregnancy testing.

13. **Reproductive status**: A volunteer who was born female must:

    - Agree to consistently use effective contraception (Appendix B and Appendix C) for sexual activity that could lead to pregnancy from at least 21 days prior to enrollment through 3 months after the last vaccination. Effective contraception is defined as using 1 of the following methods.
        - Intrauterine device (IUD), or
        - Hormonal contraception, or
        - Any other contraceptive method approved by the HVTN 702 PSRT;
    - Or not be of reproductive potential, such as having been diagnosed with premature menopause (with no menses for 1 year) or having undergone hysterectomy, bilateral oophorectomy, or tubal ligation.

14. **Volunteers who were born female must also agree not to seek pregnancy through alternative methods**, such as artificial insemination or *in vitro* fertilization until 3 months after the last vaccination

7.2 Exclusion criteria

General

1. **Blood products** received within 90 days before first vaccination

2. **Investigational research agents** received within 30 days before first vaccination

3. **Intent to participate in another study** of an investigational research agent or any other study that requires non-HVTN HIV antibody testing during the planned duration of the HVTN 702 study

4. **Pregnant or breastfeeding**

Vaccines and other Injections

5. **HIV vaccine(s)** received in a prior HIV vaccine trial. For volunteers who have received control/placebo in an HIV vaccine trial, the HVTN 702 PSRT will determine eligibility on a case-by-case basis.

6. **Non-HIV experimental vaccine(s) received within the last 5 yea**rs in a prior vaccine trial. Exceptions may be made for vaccines that have subsequently undergone licensure. For volunteers who have received control/placebo in an experimental vaccine trial, the HVTN 702 PSRT will determine eligibility on a case-by-case basis. For volunteers who have received an experimental vaccine(s) greater than 5 years ago, eligibility for enrollment will be determined by the HVTN 702 PSRT on a case-by-case basis.

7. **Live attenuated vaccines** other than influenza vaccine received within 30 days before first vaccination or scheduled within 14 days after injection (eg, measles, mumps, and rubella [MMR]; oral polio vaccine [OPV]; varicella; yellow fever)

8. **Influenza vaccine or any vaccines that are not live attenuated vaccines** and were received within 14 days prior to first vaccination (eg, tetanus, pneumococcal, Hepatitis A or B)

Immune System

9. **Immunosuppressive medications** received within 168 days before first vaccination. (Not exclusionary: [1] corticosteroid nasal spray; [2] inhaled corticosteroids; [3] topical corticosteroids for mild, uncomplicated dermatitis; or [4] a single course of oral/parenteral corticosteroids at doses < 2 mg/kg/day and length of therapy < 11 days with completion at least 30 days prior to enrollment.)

10. **Serious adverse reactions to vaccines or to vaccine components such as eggs, egg products, or neomycin** including history of anaphylaxis and related symptoms such as hives, respiratory difficulty, angioedema, and/or abdominal pain. (Not excluded from participation: a volunteer who had a non-anaphylactic adverse reaction to pertussis vaccine as a child.)

11. **Immunoglobulin** received within 60 days before first vaccination

12. **Immunodeficiency**

Clinically significant medical conditions

13. **Clinically significant medical condition**, physical examination findings, clinically significant abnormal laboratory results, or past medical history with clinically significant implications for current health. A clinically significant condition or process includes but is not limited to:

    - A process that would affect the immune response,
    - A process that would require medication that affects the immune response,
    - Any contraindication to repeated injections or blood draws,
    - A condition that requires active medical intervention or monitoring to avert grave danger to the volunteer's health or well-being during the study period,
    - A condition or process for which signs or symptoms could be confused with reactions to vaccine, or
    - Any condition specifically listed among the exclusion criteria below.

14. **Any medical, psychiatric, occupational, or other condition** that, in the judgment of the investigator, would interfere with, or serve as a contraindication to, protocol adherence, assessment of safety or reactogenicity, or a volunteer's ability to give informed consent

15. **Psychiatric condition that precludes compliance with the protocol**. Specifically excluded are persons with psychoses within the past 3 years, ongoing risk for suicide, or history of suicide attempt or gesture within the past 3 years.

16. **Active tuberculosis (TB) disease**

17. **Uncontrolled hypertension**: systolic blood pressure (SBP) $\geq$ 160 mm Hg or diastolic blood pressure (DBP) $\geq$ 100 mm Hg

18. **Bleeding disorder** (diagnosed by a doctor) contraindicating IM injection and/or blood draws, based on investigator's judgment

19. **Malignancy** (Not excluded from participation: Volunteer who has had malignancy excised surgically and who, in the investigator's estimation, has a reasonable assurance of sustained cure or who is unlikely to experience recurrence of malignancy during the period of the study)

20. History of hereditary **angioedema**, acquired angioedema, or idiopathic angioedema

7.3 Participant departure from vaccination schedule or withdrawal

This section concerns an individual participant's departure from the vaccination schedule. Pause rules for the trial as a whole are described in Section 12.3.

7.3.1 Delaying vaccinations for a participant

Under certain circumstances, a participant's scheduled vaccination will be delayed. The factors to be considered in such a decision include but are not limited to the following:

- Within 45 days prior to any study injection
  - Receipt of blood products or immunoglobulin
- Within 30 days prior to any study injection
  - Receipt of live attenuated vaccines other than influenza vaccine
  - Receipt of allergy treatment with antigen injections
- Within 14 days prior to any study injection
  - Receipt of influenza vaccine or any vaccines that are not live attenuated vaccines (eg, pneumococcal)
- Pre-vaccination abnormal vital signs or clinical symptoms that may mask assessment of vaccine reaction.
- Pregnancy: For participants, who become pregnant, no study vaccinations will be given; except for participants who may have been pregnant during the study but are no longer pregnant as shown by two negative urine pregnancy tests taken from two different urine samples; in this circumstance, the HVTN 702 PSRT should be consulted to determine if the participant may resume vaccinations.

Vaccinations should not be administered outside the visit window period specified in the HVTN 702 Study Specific Procedures (SSP).

In order to avoid vaccination delays and missed vaccinations, participants who plan to receive licensed vaccines should be counseled to schedule receipt of these

substances, when possible, outside the intervals indicated above. The effects of these substances on safety and immunogenicity assessments and their interactions with study vaccines are unknown. Therefore, if circumstances allow, these substances should also be avoided in the 2-week interval between a study vaccination and the next visit.

7.3.2 Participant departure from vaccination schedule

Every effort should be made to follow the vaccination schedule per the protocol. If a participant misses a vaccination and the visit window period for the vaccination has passed, that vaccination cannot be given. The participant should be asked to continue study visits. The participant should resume the vaccination schedule with the next vaccination unless there are circumstances that require further delay or permanent discontinuation of vaccination (Sections 7.3.1 and 7.3.3).

7.3.3 Discontinuing vaccination for a participant

Under certain circumstances, an individual participant's vaccinations will be permanently discontinued. Specific events that will result in stopping a participant's vaccination schedule include:

- Co-enrollment in a study with an investigational research agent (rare exceptions allowing for the continuation of vaccinations may be granted with the unanimous consent of the HVTN 702 PSRT).

- Clinically significant condition (ie, a condition that affects the immune system or for which continued vaccinations and/or blood draws may pose additional risk), including but not limited to the following:
  - Pregnancy: Vaccinations will be stopped while a participant is pregnant. If the participant is no longer pregnant and can be vaccinated within an appropriate visit window, vaccinations may resume (Section 7.3.1);
  - Any grade 4 local or systemic reactogenicity symptom, lab abnormality, or AE that is subsequently considered to be related to vaccination;
  - Any grade 3 lab abnormality or other clinical AE (exception: fever or vomiting and subjective local and systemic symptoms) that is subsequently considered to be related to vaccination; upon review, the PSRT may allow continuation of vaccination if the participant has grade 3 erythema and/or induration; or
  - Clinically significant type 1 hypersensitivity reaction associated with study vaccination. Consultation with the HVTN 702 PSRT is required prior to subsequent vaccinations following any type 1 hypersensitivity reaction associated with study vaccination; or

- Investigator determination in consultation with Protocol Team leadership (eg, for repeated nonadherence to study staff instructions).

Such participants should be counseled on the importance of continuing with the study and strongly encouraged to participate in follow-up visits and protocol-related procedures per the protocol for the remainder of the trial, unless medically contraindicated.

In addition, vaccinations will be stopped for participants diagnosed with HIV infection.

7.3.4 Participant termination from the study

Under certain circumstances, an individual participant may be terminated from participation in this study. Specific events that will result in early termination include:

- Participant refuses further participation,
- Participant relocates and remote follow-up or transfer to another HVTN CRS is not possible,
- HVTN CRS determines that the participant is lost to follow-up,
- Investigator decides, in consultation with Protocol Team leadership, to terminate participation (eg, if participant exhibits inappropriate behavior toward clinic staff).
- Any condition where termination from the study is required by applicable regulations.

8 Study product preparation and administration

CRS pharmacists should consult the Pharmacy Guidelines and Instructions for DAIDS Clinical Trials Networks for standard pharmacy operations. The protocol schema is shown in Table 3-1. See the IBs for further information about study products.

8.1 Vaccine regimen

The schedule of vaccination is shown in Section 3 and additional information is given below.

Group 1

Treatment 1 (T1): ALVAC-HIV (vCP2438) to be administered as 1 mL IM in LEFT deltoid (unless medically contraindicated) at months 0, 1, 3, 6, 12, and 18

AND

Bivalent Subtype C gp120/MF59 (an admixture of 100 mcg of TV1.C gp120, 100 mcg of 1086.C gp120, and MF59C.1) to be administered as 0.5 mL IM in RIGHT deltoid (unless medically contraindicated) at Months 3, 6, 12, and 18

Group 2

Placebo 2 (P2): Placebo for ALVAC-HIV (Sodium Chloride for Injection, 0.9%) to be administered as 1 mL IM in LEFT deltoid (unless medically contraindicated) at months 0, 1, 3, 6, 12, and 18

AND

Placebo for Bivalent Subtype C gp120/MF59 (Sodium Chloride for Injection, 0.9%) administered as 0.5 mL IM in RIGHT deltoid (unless medically contraindicated) at Months 3, 6, 12, and 18

8.2 Study product formulation

ALVAC-HIV (vCP2438) [Labeled as ALVAC-HIV (vCP2438)]

ALVAC-HIV (vCP2438) is provided as a lyophilized, white to beige product. It must be stored refrigerated (2-8°C). Once reconstituted with 1 mL of Diluent 0.4% NaCl, it appears as a clear to slightly opalescent solution, colorless with possible presence of particles or filaments.

The study product is described in further detail in the IB.

Diluent for ALVAC-HIV (vCP2438)

The diluent is provided in a vial filled with a volume to deliver 0.5 mL of sterile sodium chloride solution (NaCl 0.4%). Two vials of diluent are required to prepare one dose. It must be stored refrigerated (2-8°C). DO NOT FREEZE.

Placebo for ALVAC-HIV (Sodium Chloride for Injection, 0.9%)

Sodium Chloride for Injection, 0.9% will be used as the placebo for ALVAC-HIV. Product must be stored as directed by the manufacturer.

Bivalent gp120 composed of two different proteins:

TV1.C gp120 protein [labeled as TV1.C gp120]: The TV1.C gp120 protein will be provided in a glass vial containing approximately 0.58 mL (462 mcg) of protein in buffer. The protein is a clear colorless to slightly yellow liquid when thawed. The product must be stored frozen at -61°C or colder.

The study product is described in further detail in the IB.

1086.C gp120 protein [labeled as 1086.C gp120]: The 1086.C gp120 protein will be provided in a glass vial containing approximately 0.58 mL (462 mcg) of protein in buffer. The protein is a clear colorless to slightly yellow liquid when thawed. The product must be stored frozen at -61°C or colder.

The study product is described in further detail in the IB.

MF59 [labeled as MF59C.1] is supplied as an oil-in-water emulsion. The MF59 adjuvant has a milky white opaque appearance and is provided in a glass vial containing a total volume of 0.7 mL. The product must be stored refrigerated at 2-8°C. Do not freeze.

The study product is described in further detail in the IB.

Placebo for Bivalent Subtype C gp120/MF59 (Sodium Chloride for Injection, 0.9%)

Sodium Chloride for Injection, 0.9% will be used as the placebo for the Bivalent Subtype C gp120/MF59. Product must be stored as directed by the manufacturer.

8.3 Preparation of study products

Pharmacists should refer to USP 38 General Chapter Physical Tests / <797> Pharmaceutical Compounding - Sterile, and should follow the requirements of their country, their institution, and their pharmacy regulatory authority regarding these procedures. At a minimum, study products must be prepared in a biological

safety cabinet/isolator by appropriately trained/qualified pharmacy personnel using aseptic technique.

8.3.1 ALVAC-HIV (vCP2438)

One vial of ALVAC-HIV (vCP2438) and 2 vials of diluent (NaCl 0.4%) are needed to prepare this dose.

Before reconstitution, the pharmacist will allow the vials to equilibrate to room temperature. The pharmacist, using aseptic technique, will withdraw a total of 1 mL from the 2 vials containing diluent (NaCl 0.4%) and slowly inject the 1 mL of diluent into the vial containing the lyophilized ALVAC-HIV. The pharmacist will then set the vial aside and allow the vial to sit for up to 3 minutes to allow for dissolution of the vaccine. The pharmacist will gently swirl the vial to assure the contents are well dissolved. DO NOT SHAKE. (Note: Presence of particles or filaments in the dissolved solution is possible). The study product is stable in the vial for 6 hours after reconstitution. Using aseptic technique, the pharmacist will then withdraw the total contents of the ALVAC-HIV vial into a 2 to 5 mL syringe. The pharmacist will apply an overlay to the syringe.

The syringe should be labeled as "ALVAC-HIV or placebo 1 mL" as well as "Administer in Left Deltoid". Once the dose is drawn up in a syringe, the study product should be administered as soon as possible within 30 minutes (per Immunization Action Coalition [IAC] and US Centers for Disease Control and Prevention [CDC] recommendations).

Any unused portion of reconstituted vials or expired prefilled syringes is disposed of in accordance with institutional or pharmacy policy for a biological safety level 1 product.

8.3.2 Placebo for ALVAC-HIV

Using aseptic technique, the pharmacist will withdraw 1 mL of Sodium Chloride for Injection, 0.9% into a 2 to 5 mL syringe. The pharmacist will apply an overlay to the syringe.

The syringe should be labeled as "ALVAC-HIV or placebo 1 mL" as well as "Administer in Left Deltoid". Once the dose is drawn up in a syringe, the study product should be administered as soon as possible within 30 minutes (per IAC and US CDC recommendations)

Any unused portion of entered vials or expired prefilled syringes should be disposed of in accordance with institutional or pharmacy policy.

8.3.3 Bivalent Subtype C gp120/MF59 vaccine

One vial of TV1.C gp120 protein, one vial of 1086.C gp120 protein, and one vial of MF59C.1 will be needed to prepare the dose. Prior to dispensing, the

pharmacist will remove the TV1.C gp120 and 1086.C gp120 vials from the freezer and allow to thaw at room temperature. The pharmacist will also remove the MF59C.1 vial from the refrigerator and mix by repeated gentle swirling and inversion (do not shake vigorously). (Note: Once thawed the 1086.C gp120 and/or TV1.C gp120 vials should be used immediately for preparation or stored in a refrigerator at 2-8°C for no longer than 24 hours. Unused 1086.C gp120 and/or TV1.C gp120 protein vials should be quarantined for destruction after this time.)

Using aseptic technique, the pharmacist will gently swirl the contents of the vial containing TV1.C gp120 and then withdraw 0.35 mL of TV1.C gp120 from the correct vial and inject it into the vial containing MF59C.1. The pharmacist will then gently swirl the vial containing 1086.C gp120 after which, using aseptic technique, the pharmacist will withdraw 0.35 mL of 1086.C gp120 from the correct vial and inject it into the MF59C.1 vial (which contains TV1.C gp120 and MF59C.1). After gentle swirling and inversion (do not shake vigorously) the pharmacist, using aseptic technique, will withdraw 0.5 mL of the mixed preparation for dosing into a 1 to 2 mL syringe. The pharmacist will apply an overlay to the syringe.

The syringe should be labeled as “Bivalent Subtype C gp120/MF59 or Placebo 0.5 mL”, as well as “Administer in RIGHT deltoid”. The syringe containing study product should be bagged for transport to the clinic where it will be administered. This study product should be administered immediately, defined as within 30 minutes as per IAC recommendations. If this is not possible, the vaccine should be stored at 2°C-8°C until administration and if not used within 2 hours, it should be discarded.”

Any unused portion of entered vials or expired prefilled syringes should be disposed of in accordance with institutional or pharmacy policy.

8.3.4 Placebo for Bivalent Subtype C gp120/MF59 vaccine

Using aseptic technique, the pharmacist will withdraw 0.5 mL of Sodium Chloride for Injection, 0.9% into a 1 to 2 mL syringe. The pharmacist will apply an overlay to the syringe.

The syringe should be labeled as “Bivalent Subtype C gp120/MF59 or Placebo 0.5 mL”, as well as “Administer in RIGHT deltoid”. The syringe containing study product should be bagged for transport to the clinic where it will be administered. This study product should be administered immediately, defined as within 30 minutes as per IAC recommendations. If this is not possible, the vaccine should be stored at 2°C-8°C until administration and if not used within 2 hours, it should be discarded.”

Any unused portion of entered vials or expired prefilled syringes should be disposed of in accordance with institutional or pharmacy policy.

8.4 Administration

All injections are to be given IM in the deltoid indicated. Any administrator of study product must be blinded to the individual participant's treatment assignment.

When preparing a dose in a syringe and administering the dose, consideration should be given to the volume of solution in the needle before and after the dose is administered. Particularly, if the needle used to withdraw the product is replaced prior to vaccine administration, consideration should be given to conserving the full dose of product. The pharmacy and clinic staff members are encouraged to work together to administer the dose specified in the protocol.

All injections are to be given using standard IM injection technique.

For all syringes containing Bivalent gp120/MF59 or placebo, the person administering the injection should gently roll the syringe prior to administration of the study product.

If an injection is administered in the contralateral deltoid due to a medical contraindication, the appropriate study staff should document this clearly. Under this circumstance, this is NOT a protocol violation. Two injections administered into the same deltoid should be at least 2.4 cm apart and should be documented in the participant's study record.

8.5 Acquisition of study products

The ALVAC-HIV (vCP2438) will be supplied (along with the Diluent) by Sanofi Pasteur and will be available through the NIAID Clinical Research Products Management Center (CRPMC).

Bivalent Subtype C gp120 and MF59 will be supplied by GlaxoSmithKline Biologicals, S.A. and will be available through the CRPMC.

Placebo for ALVAC-HIV and Placebo for Bivalent Subtype C gp120/MF59 (Sodium Chloride for Injection, 0.9%) will not be provided through the protocol and must be obtained by the site.

Once an HVTN CRS is protocol registered, the pharmacist can obtain study products from the CRPMC by following the ordering procedures outlined in the Pharmacy Guidelines and Instructions for DAIDS Clinical Trials Networks.

8.6 Pharmacy records

The HVTN CRS pharmacist is required to maintain complete records of all study products. The pharmacist of record is responsible for maintaining randomization

codes and randomization confirmation notices for each participant in a secure manner.

8.7 Final disposition of study products

All unused study products must be returned to the CRPMC after the study is completed or terminated unless otherwise instructed by the CRPMC. The procedures and relevant form are included in the Pharmacy Guidelines and Instructions for DAIDS Clinical Trials Networks.

9 Clinical procedures

The schedules of clinical procedures are shown in Appendix H, Appendix I, and Appendix N.

All study data will be recorded on paper source documents prior to being entered into the study database (see the HVTN 702 SSP).

9.1 Informed consent

Informed consent is the process of working with participants so that they fully understand what will and may happen to them while participating in a research study. The HVTN informed consent form documents that a participant (1) has been informed about the potential risks, benefits, and alternatives to participation, and (2) is willing to participate in an HVTN study. Informed consent encompasses all written or verbal study information HVTN CRS staff provide to the participant, before and during the trial. HVTN CRS staff will obtain informed consent of participants according to HVTN policies and procedures.

The informed consent process continues throughout the study. Key study concepts should be reviewed periodically with the participant and the review should be documented. At each study visit, HVTN CRS staff should consider reviewing the procedures and requirements for that visit and for the remaining visits. Additionally, if any new information is learned that might affect the participants' decisions to stay in the trial, this information will be shared with trial participants. If necessary, participants will be asked to sign revised informed consent forms.

An HVTN CRS may employ recruitment efforts prior to the participant consenting. For example, some HVTN CRSs use a telephone script to prescreen people before they come to the clinic for a full screening visit. Participants must sign a screening or protocol-specific consent before any procedures are performed to determine eligibility. HVTN CRSs must submit recruitment and prescreening materials to their IRB/EC and any applicable Regulatory Entity (RE) for human subjects protection review and approval.

Note: As defined in the DAIDS Protocol Registration Manual, an RE is "Any group other than the local IRB/EC responsible for reviewing and/or approving a clinical research protocol and site-specific ICFs [informed consent forms] prior to implementation at a site." CRSs are responsible for knowing the requirements of their applicable REs.

9.1.1 Screening consent form

Without a general screening consent, screening for a specific study cannot take place until the site receives protocol registration from the DAIDS RSC Protocol Registration Office.

Some HVTN CRSs have approval from their IRB/EC and any applicable RE to use a general screening consent form that allows screening for an unspecified HIV vaccine trial. In this way, HVTN CRS staff can continually screen potential participants and, when needed, proceed quickly to obtain protocol-specific enrollment consent. Sites conducting general screening or prescreening approved by their IRB/EC and any applicable RE may use the results from this screening to determine eligibility for this protocol, provided the tests are conducted within the time periods specified in the eligibility criteria.

9.1.2 Protocol-specific consent forms

The protocol-specific consent forms describe the study products to be used and all aspects of protocol participation, including screening and enrollment procedures. A sample protocol-specific consent form for the main study is located in Appendix A. A separate sample consent form for other uses of specimens is located in Appendix D. A post-unblinding addendum to the sample informed consent form is located in Appendix K.

Each HVTN CRS is responsible for developing a protocol-specific consent form(s) for local use, based on the sample protocol-specific consent forms in Appendix A, Appendix D, and Appendix K. The consent form(s) must be developed in accordance with requirements of the following:

- CRS's IRB/EC, and any applicable REs;
- CRS's institution; and
- Elements of informed consent as described in Title 45, CFR Part 46 and Title 21 CFR, Part 50, and in the International Conference on Harmonisation (ICH) E6, Good Clinical Practice: Consolidated Guidance 4.8.

Study sites are strongly encouraged to have their local CABs review their sites-specific consent forms. This review should include, but should not be limited to, issues of cultural competence, local language considerations, and the level of understandability.

The sample informed consent form includes instructions throughout the document for developing specific content.

Sites should follow the instructions in the Protocol-specific Official Memo distributed along with this protocol regarding when they may begin using their site-specific protocol consent forms.

Regarding protocol registration, sites should follow procedures outlined in the current version of the DAIDS Protocol Registration Manual.

9.1.3 Assessment of understanding

Study staff are responsible for ensuring that participants fully understand the study before enrolling them. This process involves reviewing the informed consent form with the participant, allowing time for the participant to reflect on the procedures and issues presented, and answering all questions completely.

An assessment of understanding is used to document the participant's understanding of key concepts in this HIV vaccine trial. This process and the participant's understanding of the key concepts should be recorded in source documentation at the site.

IRB/EC and any applicable RE may require that a participant has signed either a screening or protocol-specific consent document prior to administering the Assessment of Understanding. The consent process (including the use of the Assessment of Understanding) should be explained thoroughly to the IRB/EC and any applicable RE, whose recommendations should be followed.

9.2 Pre-enrollment procedures

Screening may occur over the course of several contacts/visits, up to and including before vaccination on Day 0. All inclusion and exclusion criteria must be assessed within 56 days before enrollment with the exception of HIV testing, which must be performed within 30 days before enrollment.

After the appropriate informed consent has been obtained and before enrollment, the following procedures are performed:

- Medical history, documented in the case history record;
- Complete physical examination, including height, weight, vital signs, and male circumcision status; clinical assessments of head, ears, eyes, nose, and throat; neck; lymph nodes; heart; chest; abdomen; extremities; neurological function; and skin. Note: A man who wants to enroll in the study, but who is not circumcised and wishes to be, will be referred to a practitioner for circumcision, but the study sponsors will not pay for the procedure.
- Assessment of concomitant medications and contraceptives the volunteer is taking, including prescription drugs (eg, ARVs) and nonprescription drugs, vitamins, topical products, alternative/complementary medicines (eg, herbal and health food supplements), recreational drugs, vaccinations, and allergy shots;
- Laboratory tests as defined in the inclusion and exclusion criteria, including:
    - Screening HIV testing at local lab;
    - ALT

    - Urine or serum pregnancy test (volunteers who were born female),

- Administration of behavioral risk assessment questionnaire;

- Obtaining of volunteer demographics in compliance with the NIH Policy on Reporting Race and Ethnicity Data: Subjects in Clinical Research, Aug. 8, 2001 (available at http://grants.nih.gov/grants/guide/notice-files/NOT-OD-01-053.html);

- Counseling on HIV testing and risk reduction, as described in Section 9.7; and

- Discussion of pregnancy prevention. A pregnant or breastfeeding person may not be enrolled in this trial. Specific criteria and assessment of contraception and pregnancy status are described in study inclusion criteria.

9.2.1 Use of screening results from another HVTN study

If a participant screens for an HVTN study at the same HVTN CRS but then does not join that study, screening results from that effort may be applied to the screening for this protocol, as long as the screening was done under participant consent, the participant has signed a consent form to begin screening for this study, and the tests were conducted within the time periods specified in the eligibility criteria (Sections 7.1 and 7.2).

9.3 Enrollment and vaccination visits (pre-unblinding)

Enrollment is simultaneous with first vaccination. The time interval between randomization and enrollment should not exceed 4 working days. The HVTN CRS registers the participant by scheduling the day 0 visit (enrollment) via the Web-based randomization system, and requests the randomization assignment. Circumstances may require a participant's enrollment visit to be changed. This may exceed the 4-day randomization time limit.

At all vaccination visits, the following procedures are performed before vaccination:

Abbreviated physical examination, including weight, vital signs, and a symptom-directed evaluation by history and/or appropriate physical exam based on participant self-reported symptoms or complaints; Assessment of baseline reactogenicity parameters;

- Assessment of concomitant medications (as described in Section 9.2);

- Assessment of any new or unresolved AEs/intercurrent illnesses; and

- Urine or serum pregnancy test (for participants who were born female). Persons who are NOT of reproductive potential due to having undergone total

hysterectomy or bilateral oophorectomy (verified by medical records), are not required to undergo pregnancy testing.

Following completion of all procedures in the preceding list, and if results indicate that vaccination may proceed, vaccination is prepared and administered (Sections 8.3 and 8.4).

Administration of all injections during a vaccination visit must be accomplished within 1 calendar day.

Immediately following vaccination, the participant remains in the clinic for observation. An initial reactogenicity assessment is made at a target of 30 minutes after injection, with an acceptable range of 25-60 minutes. Before leaving the clinic, the participant will be given the postvaccination memory tool and instructed on how to use it. The site will make arrangements to obtain a report of reactogenicity events from the participant after the 3-day reactogenicity period (as described in Section 9.9).

The following procedures will be performed at all vaccination visits. These procedures may be performed prior to or following vaccination:

- Risk reduction counseling (as described in Section 9.7);
- Pregnancy prevention assessment (as described in Section 9.2 and 9.8); and
- Assessment of new or unresolved social impacts (CRS staff will ask participant about the status of any unresolved social impacts and if s/he has experienced any new social impacts as a result of the trial participation).

Additional procedures will be performed at scheduled visits as specified in Appendix H:

- HIV infection assessment including pre-test counseling. A subsequent follow-up contact is conducted to provide post-test counseling and to report results to the participant;
- Behavioral risk assessment questionnaire;
- Outside testing and belief questionnaire;
- CBC/differential;
- Hgb;
- Confirm that participants received HIV test results from previous visit. If not, provide test results and post-test counseling as appropriate; and
- Syphilis serology testing,

- Gonorrhea (GC)/chlamydia (CT) testing by urine or cervical/vaginal swab for persons born female; by urine for persons born male; and additionally by rectal swab for transgender persons (TG) and MSM,
- Trichomonas by cervical/vaginal swab, and
- With the exception of samples collected for STI testing and for ARV detection by dried blood spots (see Appendix H, footnote "b"), specimen collection (blood and/or stool) should be completed prior to vaccination.

9.4 Follow-up visits (pre-unblinding)

The following procedures are performed at all scheduled follow-up visits:

- Risk reduction counseling (as described in Section 9.7);
- Pregnancy prevention assessment (as described in Section 9.2 and 9.8);
- Assessment of new or unresolved social impacts (site staff will ask participant about the status of any unresolved social impacts and if s/he has experienced any new social impacts as a result of the trial participation);
- Assessment of new or continuing concomitant medications (as described in Section 9.2); and
- Assessment of new or unresolved AEs/intercurrent illnesses.

Additional procedures will be performed at scheduled follow-up visits as specified in Appendix H:

- Abbreviated physical examination including weight, vital signs, and a symptom-directed evaluation by history and/or appropriate physical exam based on participant self-reported symptoms or complaints;
- Complete physical examination, including weight, vital signs, and clinical assessments of head, ears, eyes, nose, and throat; neck; lymph nodes; heart; chest; abdomen; extremities; neurological function; skin; and, if applicable, male circumcision status
- Outside testing and belief questionnaire
- Administration of behavioral risk assessment questionnaire;
- HIV infection assessment including pre-test counseling. A subsequent follow-up contact is conducted to provide post-test counseling and to report results to participant;

- Confirm that participants received HIV test results from previous visit. If not, provide test results and post-test counseling as appropriate;

- Specimen collection (blood and/or stool);

- Syphilis serology testing,

- Gonorrhea (GC)/chlamydia (CT) testing by urine or cervical/vaginal swab for persons born female; by urine for persons born male; and additionally by rectal swab for TG and MSM,

- Trichomonas by cervical/vaginal swab, and

- Urine or serum pregnancy test (for participants who were born female). Persons who are NOT of reproductive potential due to having undergone total hysterectomy or bilateral oophorectomy (verified by medical records), are not required to undergo pregnancy testing.

9.4.1 Follow-up visits (post-unblinding)

Following participant unblinding, the following procedures are performed at all scheduled follow-up visits to 12 months following the participant's last vaccination:

- Risk reduction counseling (as described in Section 9.7);

- Assessment of new or unresolved social impacts (site staff will ask participant about the status of any unresolved social impacts and if s/he has experienced any new social impacts as a result of the trial participation);

- Assessment of new or continuing concomitant medications (as described in Section 9.2);

- Assessment of new or unresolved AEs/intercurrent illnesses.

Additional procedures will be performed at scheduled follow-up visits as specified in Appendix M and Appendix N:

- Abbreviated physical examination including weight, vital signs, and a symptom-directed evaluation by history and/or appropriate physical exam based on participant self-reported symptoms or complaints;

- Complete physical examination, including weight, vital signs, and clinical assessments of head, ears, eyes, nose, and throat; neck; lymph nodes; heart; chest; abdomen; extremities; neurological function; skin; and, if applicable, male circumcision status

- Administration of behavioral risk assessment questionnaire;

- HIV infection assessment including pre-test counseling. A subsequent follow-up contact is conducted to provide post-test counseling and to report results to participant;

- Confirm that participants received HIV test results from previous visit. If not, provide test results and post-test counseling as appropriate;

- Specimen collection (blood);

- Syphilis serology testing,

- Gonorrhea (GC)/chlamydia (CT) testing by urine or cervical/vaginal swab for persons born female; by urine for persons born male; and additionally, by rectal swab for TG and MSM, and

- Trichomonas by cervical/vaginal swab.

At a participant's final scheduled clinic visit (ie, termination visit), the laboratory and CRS procedures specified for Visit 16 in Appendix M and Appendix N should be performed.

In order to ensure participant and staff safety during the COVID-19 pandemic, "clinic visits" for purposes of data collection and procedures may be conducted by phone, text message, email, or other electronic means. An in-person clinic visit is required only for physical exam, point-of-care testing, and collecting biological samples, where these can be done safely.

9.5 Stool sampling

Two stool samples will be collected from the study participants who agree to this procedure: 1 prior to enrollment (before the injection of the vaccine) and 1 at the Month 6.5 timepoint. These samples will be collected using swabs, either via rectal swabs or by taking swabs from stool.

9.6 Procedures for HIV-infected participants

The following procedures are performed at follow-up visits for HIV-infected participants, as specified in Appendix I:

- Counseling on HIV-1 testing/diagnosis;

- Abbreviated physical examination including weight, vital signs, and a symptom-directed evaluation by history and/or appropriate physical exam based on participant self-reported symptoms or complaints;

- Complete physical examination, including clinical assessments of head, ears, eyes, nose, and throat; neck; lymph nodes; heart; chest; abdomen; extremities; neurological function; and skin;
- Assessment of HIV/AIDS-related conditions and non-HIV disease progression-related events;
- Assessment of new or continuing concomitant medications (as described in Section 9.2);
- Assessment of new or continuing antiretroviral therapies and reason for initiation;
- Assessment of new or unresolved AEs/intercurrent illnesses;
- Counseling to reduce the risk of HIV transmission;
- Administration of behavioral risk assessment questionnaire;
- Assessment of new or unresolved social impacts (site staff will ask participant about the status of any unresolved social impacts and about any new social impacts experienced as a result of the trial participation);
- Specimen collection (Appendix G).

9.7 HIV counseling and testing

HIV counseling will be performed in compliance with the US Centers for Disease Control and Prevention (CDC) guidelines or other local guidelines for HIV counseling and referral. HIV testing will be performed in accordance with the current HVTN HIV testing algorithm following enrollment.

Participants will be counseled routinely during the trial on the avoidance of HIV infection and on the potential negative social impacts of testing antibody positive due to the vaccine. They will also be counseled on the risks of HIV antibody testing outside of the HVTN CRSs and will be discouraged from doing so during study participation and/or during any period of vaccine-induced positive serology.

Following study unblinding, participants who did not receive HIV vaccine study product or who have been determined to not have vaccine-induced positive serology (VISP) may have HIV testing performed within the community.

Study staff will take particular care to inform study participants of the likelihood of routine HIV testing being offered or performed outside the study CRS at emergency rooms, clinics, and medical offices. Such testing has become more likely due to the CDC's revised guidelines for HIV counseling and testing, as well as policy changes in many countries to make HIV testing more frequent and

routine. CRS staff should inform participants of their right to opt out of HIV testing outside the study site. CRS staff should inform study participants if local and/or state policies and regulations permit medical providers to perform HIV testing without first informing patients. If this is the case, then CRS staff should advise study participants that they may decline testing preemptively. CRS staff should also inform participants if positive results must be reported to local public health authorities. CRS staff should also inform participants of the need to maintain study blinding by getting HIV testing only at the study CRS. CRS staff should provide participants with CRS contact information and should encourage participants to ask medical providers to contact the CRS. The CRS can verify that the participant is in an HIV vaccine clinical trial and should only be tested at the study CRS.

9.7.1 Distinguishing intercurrent HIV infection from vaccine-induced positive serology

The study product may elicit an antibody response to HIV proteins. Therefore, vaccine-induced positive serology may occur in this study. Several precautionary measures will be taken to distinguish intercurrent HIV infection from vaccine-induced positive serology. These precautionary measures include:

- Participants will have physical examinations at visits specified in Appendix H. Signs or symptoms of an acute HIV infection syndrome, an intercurrent illness consistent with HIV-1 infection, or probable HIV exposure would prompt a diagnostic workup per the HVTN algorithm for Recent Exposure/Acute Infection Testing to determine HIV infection.

- HIV testing will be performed at multiple timepoints throughout the study (Appendix F). The Laboratory Program (or approved diagnostic laboratory) will follow the HVTN HIV testing algorithm (see *Study Specific Procedures* [SSP]), which is able to distinguish vaccine-induced antibody responses from actual HIV infections.

- All participants can receive HIV-1 diagnostic testing from the site until they are told that they did not receive an HIV vaccine or that they do not have vaccine-induced seropositivity (VISP).

- All participants who received vaccine product and who have vaccine-induced positive or indeterminate HIV-1 serology (as measured by the standard anti-HIV antibody screening tests) at or after the study is unblinded will be offered poststudy HIV-1 diagnostic testing (per the HVTN poststudy HIV-1 testing algorithm) periodically and free of charge as medically/socially indicated (approximately every 6 months) unless or until HIV Ab testing is no longer the standard test in clinical settings.

9.7.2 VISP registry

Experimental HIV vaccines may induce antibody production to HIV antigens, producing reactive results on commercially available HIV test kits. In order to provide poststudy HIV testing to distinguish between VISP and HIV infection, and to mitigate potential social harms resulting from VISP in HIV vaccine recipients who are not infected with HIV, the HVTN has created a VISP registry. Following study unblinding, the registry will allow trained staff to verify that an individual has received an HIV vaccine, and therefore has the potential for VISP. Information in the VISP registry will not be used for research. Rather, the registry exists to support provision of poststudy testing and counseling services to HIV vaccine recipients. The registry contains the names of all study participants, unless they request that their names be removed.

9.8 Pregnancy prevention assessment

Per Section 7.1, use of effective contraception is required from 21 days prior to enrollment (ie, first vaccination) until 3 months after the last vaccination for participants who were born female and who are of reproductive potential (capable of becoming pregnant). Prior to enrollment and throughout the period for which contraceptive use is required, staff will ask these participants to verbally confirm their use of adequate contraceptive methods. During this period, participants for whom contraceptive use is required should be reminded at all scheduled clinic visits of the importance of using contraception and should be referred to specific counseling, information, and advice as needed. This reminder should be documented in the participant’s study record.

Self-reported infertility—including having been diagnosed with premature menopause (with no menses for 1 year) or having undergone hysterectomy, bilateral oophorectomy, or tubal ligation—must be documented in the participant’s study record.

9.9 Assessments of reactogenicity

For all participants, baseline assessments are performed before and reactogenicity assessments are performed after each vaccination. All reactogenicity symptoms are followed until resolution and graded according to the *Division of AIDS Table for Grading the Severity of Adult and Pediatric Adverse Events*, Corrected Version 2.1, dated July 2017, except as noted in Section 12.2.2.

The reactogenicity assessment period is 3 full days following each vaccination. The maximum severity reached for each symptom during the assessment period is reported on CRFs. Participants will be given a postvaccination memory tool to assist with recall of symptoms. The site staff and the participant will be in contact after the 3-day reactogenicity period, or sooner if indicated. At that time, site staff will discuss reactogenicity events with the participant and will record the relevant

information on either the appropriate CRF(s) or participant chart note. Clinic staff will follow new or unresolved reactogenicity symptoms present at day 3 to resolution. Participants are instructed to contact the clinic for events that arise during the period between vaccination and the next scheduled visit. In general, a participant who self-reports any postvaccination reaction greater than mild is seen by a clinician within 48 hours after onset, unless the reaction is improving and/or has resolved completely.

Reactogenicity assessments include assessments of systemic and local symptoms and vaccine-related lesions. Events not listed on a CRF, or with an onset after the reactogenicity assessment period (day of vaccination and 3 full days after), or those meeting SAE/AEs requiring expedited reporting criteria, are recorded on an AE log form.

9.9.1 Assessment of systemic and local symptoms

Systemic symptoms include increased body temperature, malaise and/or fatigue, myalgia, headache, chills, arthralgia, nausea, and vomiting. Local symptoms include pain and/or tenderness proximal to the injection site. Body temperature is measured by oral or infrared thermometry and reported in degrees Celsius. If temperature is measured in Fahrenheit, the conversion to Celsius should be documented in the participant's chart note. A measurement is taken once daily during the assessment period and should be repeated if participant is feeling feverish. Body temperature measurement is not required during the in-clinic reactogenicity assessment immediately following vaccination.

9.9.2 Assessment of injection site

Typical injection site reactions are erythema/redness and induration/swelling. The maximum horizontal and maximum vertical measurements for all injection site reactions are recorded.

All injection site reactions are monitored until resolution. Areas greater than 25 cm^2 are followed daily; otherwise, the frequency of follow-up is based on clinician judgment.

9.10 Visit windows and missed visits

Visit windows are defined in HVTN 702 Study Specific Procedures. The procedures for documenting missed visits and out-of-window visits are described in HVTN 702 Study Specific Procedures. If the missed visit is one that required safety assessments, HVTN CRS staff should attempt to bring the participant in for an interim visit as soon as possible.

Procedures performed at an interim visit are usually toxicity/safety assessments (including local safety labs) and HIV testing. With the exception of HIV testing, these procedures are performed only if they were required at the missed visit or if

clinically indicated. HIV testing may be performed as deemed appropriate by the study staff. Blood samples for immunogenicity assays are not typically collected at interim visits.

If a missed visit required vaccination, please refer to Section 7.3.2 and Section 7.3.3 for resolution.

9.11 Early termination visit

In the event of early participant termination, site staff should consider whether, in addition to procedures performed at all scheduled clinic visits, the following assessments are appropriate: a final physical examination, behavioral risk assessment questionnaire, STI test, and HIV test.

9.12 Pregnancy

If a participant becomes pregnant during the course of the study, no more injections of study product will be given during the pregnancy, but remaining visits and study procedures should be completed unless medically contraindicated or applicable regulations require termination from the study. For participants who are no longer pregnant, see Section 7.3.1. If the participant terminates from the study prior to the pregnancy outcome, the site should make every effort to keep in touch with the participant in order to ascertain the pregnancy outcome. All pregnancies and pregnancy outcomes should be recorded and reported (see the HVTN 702 SSP).

10 HIV-1 infection assessment and clinical response

10.1 HIV-1 symptom assessment

At scheduled visits indicated in Appendix F and Appendix H and at unscheduled visits due to illness or suspected exposure, if necessary, information will be collected about any signs or symptoms suggestive of acute HIV-1 infection. Participants will be counseled about signs and symptoms of acute HIV infection and at visits following recent high-risk exposure, participants will be queried about any signs/symptoms suggestive of acute HIV-1 infection. Presence of signs/symptoms suggestive of acute HIV-1 infection, an intercurrent illness consistent with acute retroviral syndrome, or history of high-risk exposure would prompt a diagnostic work-up per the protocol-specific algorithm to determine HIV infection, except that in this instance the algorithm is modified to conduct serology and nucleic acid testing simultaneously.

10.2 HIV-1 screening test (prior to randomization)

Prior to randomization, participants will be screened for HIV-1/2 infection by blood tests approved by the US FDA or locally available assays that have been approved by HVTN Laboratory Operations. Potential participants identified as being HIV infected during screening will be referred for medical treatment and management of the HIV infection. These individuals may also be referred to appropriate ongoing clinical trials or observational studies.

10.3 HIV-1 *testing* postvaccination

Surveillance for HIV-1 takes place at vaccination visits and follow-up visits specified in Appendix F.

In-study HIV testing will be performed using the HVTN HIV diagnostic testing algorithm. Routinely, specimens are initially assayed with an HVTN Lab Program–approved fourth-generation HIV 1/2 enzyme immunoassay (EIA) or chemiluminescent microparticle immunoassay (CMIA). If the EIA/CMIA is reactive, nucleic acid polymerase chain reaction (PCR) test will be performed as indicated in the algorithm. The algorithm is repeated on a second specimen to confirm a diagnosis of HIV-1 infection. The second specimen for confirmatory testing may be collected at an interim visit (designated as visit #.X, where # is the visit at which the first reactive test was obtained and X designates the interim visit; specified in Appendix G and Appendix I). Samples to be stored for future immunogenicity or virology studies will also be collected at this time (Appendix G).

A 'case' will be defined as a participant with confirmed detectable HIV-1 nucleic acid PCR on 2 different specimen collection dates. The nucleic acid test will most

commonly be the HIV-1 RNA VL PCR test. Confirmation of HIV-1 infection will be determined through use of the HIV testing algorithm (available on the HVTN 702 protocol-specific website). Before issuing an HIV-1 infection report for a participant diagnosed with HIV-1 infection prior to study unblinding, all testing results will be reviewed by a blinded, independent Endpoint Adjudicator(s) and/or designee(s) (Section 10.4).

If a participant is confirmed to have become HIV-1 infected prior to study unblinding, plasma HIV-1 viral RNA will be measured on archived samples collected according to Appendix G. In addition to plasma HIV-1 viral RNA testing, participants will also have measurements of immunogenicity assessments and a clinical assessment performed at all of these visits.

The HVTN Laboratory Program is responsible for all in-study diagnostic HIV testing.

10.4 Endpoint adjudication

The diagnostic criteria for HIV-1 infection outside the setting of a vaccine trial are well accepted. However, definitive diagnosis of HIV-1 infection in the context of having received a vaccine that is even partially effective may be more difficult. Specifically, if the immune responses elicited by vaccination are capable of completely suppressing viral replication, or if vaccination alters the normal serological response upon exposure to HIV-1, standard diagnostic tests may be more difficult to assess. Therefore, the HVTN will have an endpoint adjudication process to assess all serological and virological testing, in a blinded manner, on each participant in the trial who, prior to study unblinding, tests positive per the HVTN 702 HIV-1 diagnostic testing algorithm. The assessment of the Endpoint Adjudicator(s) or designee(s) will be reported to the SDMC and to the HIV diagnostics laboratory.

The Endpoint Adjudicator(s) and/or designee(s) must notify the SDMC within 1 working day of any confirmed HIV-1 infection. The HIV diagnostics lab will inform the clinic of the outcome of the HIV testing algorithm (ie, HIV-infected, HIV-uninfected, or redraw required).

The Endpoint Adjudicator(s) and/or designee(s) will be an expert in the fields of infectious diseases or laboratory medicine independent of the clinical investigators participating in this trial. A separate Standard Operating Procedure will govern the activities of the Endpoint Adjudicator(s) and/or designee(s).

10.5 HIV-1 infection during the study

It is critical to the success of the study that HIV-1–infected participants be properly identified and all data postdiagnosis be carefully recorded. Information

obtained from these cases of HIV-1 infection will form the basis of the primary endpoint assessment.

Participants who develop HIV-1 infection following receipt of study product may remain in the study for follow-up but will receive no further injections. All participants who become HIV-1 infected following enrollment will be monitored as indicated in Appendix G and Appendix I Longer-term follow-up for these participants may be accomplished through enrollment in another protocol. Archived samples from earlier visits may also be tested to determine the earliest date of HIV-1 infection.

10.6 Medical care for participants who become HIV-1–infected

It is anticipated that some study participants, whether they are randomized to receive vaccine or placebo, will become HIV-1 infected during the course of the trial. It is critical that these HIV-1–infected participants receive appropriate medical care.

The investigators associated with this trial will refer participants who develop HIV infection while participating in this trial to medical professionals for care.

11 Laboratory

11.1 HVTN CRS laboratory procedures

The HVTN Site Lab Instructions and SSP provides further guidelines for operational issues concerning the clinical and processing laboratories. The manual includes guidelines for general specimen collection, special considerations for phlebotomy, specimen labeling, whole blood processing, HIV screening/diagnostic testing, and general screening and safety testing.

Tube types for blood collection are specified in Appendix F, Appendix G, and Appendix M. For tests performed locally, the local lab may assign appropriate tube types.

In specific situations, the blood collection tubes may be redirected to another laboratory or may require study-specific processing techniques. In these cases, laboratory special instructions will be posted on the protocol-specific section of the HVTN website.

11.2 Total blood volume

Required blood volumes per visit are shown in Appendix F, Appendix G, and Appendix M. Not shown is any additional blood volume that would be required if a safety lab needs to be repeated, or if a serum pregnancy test needs to be performed. The additional blood volume would likely be minimal. The total blood volume drawn for each participant will not exceed 500 mL in any 56-day (8-week) period.

11.3 Assay timepoints

The primary immunogenicity timepoint in this study is at visit 6 (day 182) (ie, 2 weeks after the fourth vaccination visit). Endpoint assays for humoral and cellular responses may be performed on participant samples at the primary immunogenicity timepoint and may be performed at baseline. Assays for humoral and cellular responses may be performed on participant samples at other timepoints; the schedules are shown in Appendix F, Appendix G, and Appendix M.

11.4 Endpoint assays: humoral

11.4.1 Binding antibody multiplex assay (BAMA)

HIV-1-specific total binding IgG antibodies may be assessed on serum samples from study participants taken at the primary immunogenicity timepoint and

baseline. In addition, HIV-1-specific total binding IgA antibodies and binding to IgG subclasses (IgG1, IgG2, IgG3, IgG4) may also be assessed. Specimens from other timepoints as well as other HIV antigens may also be assayed based on the results of the initial assay.

11.4.2 Neutralizing antibody assay

HIV-1–specific nAb assays may be performed on serum samples from study participants taken at the primary immunogenicity timepoint. Specimens from the baseline and other timepoints may also be analyzed at the discretion of the HVTN Laboratory Program, which may be contingent on the results of the primary immunogenicity timepoint. The TZM-bl assay may be used to test neutralization of the vaccine strains ZM96, 1086.C, and TV1.C and a single highly neutralization-sensitive Tier 1 virus as a positive control. The global panel and/or clade-specific panels may be used to assess Tier 2 neutralization (132, 133).

11.5 Endpoint assays: cellular

11.5.1 Flow cytometry

Flow cytometry may be used to examine vaccine-specific CD4+ and CD8+ T-cell responses following stimulation of PBMCs with synthetic HIV peptides that span the proteins encoded by the vaccine. ICS parameters for this protocol include cytokines such as IFN-γ, interleukin (IL)-2, and tumor necrosis factor (TNF)-α, and may include other cytokines (such as cytokines relevant to Th2 and Th17 responses) to identify T cells of specific functionality. Markers of cytotoxic potential (Granzyme B, perforin, and CD57) may also be included. Data will be reported as percentages of CD4+ or CD8+ T cells responding to a specific peptide pool. Additional cell surface markers, cytokines, or functional markers may also be analyzed.

11.6 Viral sequencing

Viral sequencing may be conducted on the earliest available plasma specimens with positive HIV-1 RNA PCR tests from study participants who are diagnosed with HIV-1 infection. Viral sequencing may be conducted on subsequently collected samples to assess viral evolution.

11.7 ARV detection

A direct, biomedical measure is used to assess ARV use in this study. This approach is used because self-report of ARV use for PrEP or PEP and other purposes has been shown to be unreliable in a variety of settings (134-136). For this study, ARV testing may be performed using a test that measures the amount of tenofovir-diphosphate (a metabolite of tenofovir) in red blood cells. This test

utilizes a dried blood spot as the sample source and analyzes for tenofovir diphosphate by liquid chromatography and tandem mass-spectroscopy, as previously described (137, 138). Because of a long half-life, high amounts of the metabolite in the dried blood spot correspond with consistent dosing of Truvada and low amounts correspond with inconsistent dosing (139). Samples are collected and stored on regularly scheduled calendar days throughout the study and testing will be performed periodically on batched samples. In addition, both serum and plasma samples are collected from all HIV-uninfected participants at multiple timepoints (see Appendix F). This will allow additional assessment of PrEP use by selected participants if warranted by the results of the dried blood spot assay. Individual test results will not be returned to study sites or study participants.

11.8 Genotyping

Molecular human leukocyte antigen (HLA) typing may be performed on enrolled participants using cryopreserved PBMC collected at baseline, initially on specimens from participants who demonstrate vaccine-induced T-cell responses at postvaccination timepoints. Other participants (including control recipients) may be HLA-typed to support future studies of immunological interest at the discretion of the HVTN Laboratory Program. Other markers, such as genes associated with immune responses or HIV-1 disease progression may also be assessed.

11.9 Laboratory Center assay portfolio

Additional assays may be performed per the HVTN Laboratory Center assay portfolio, which includes immune assessments such as those for cellular, humoral, and innate immune responses, and host genetics. The assay portfolio will be updated periodically to include new assays and adjust qualification levels of existing assays.

11.10 Exploratory studies

Samples may be used for other testing and research related to furthering the understanding of HIV immunology or vaccines. In addition, cryopreserved samples may be used to perform additional assays to support standardization and validation of existing or newly developed methods.

11.10.1 Microbiome analysis

Stool swab specimens are processed to enable nucleic acid sequencing. 16s rRNA sequences may then be determined using pyro-sequencing approaches or other methods.

11.11 Other use of stored specimens

The HVTN stores specimens from all study participants indefinitely, unless a participant requests that specimens be destroyed or if required by IRB/EC, or RE.

Other use of specimens is defined as studies not described in the protocol.

This research may relate to HIV, vaccines, the immune system, and other diseases. This could include limited genetic testing and, potentially, genome-wide studies. This research is done only to the extent authorized in each study site's informed consent form, or as otherwise authorized under applicable law. Other testing on specimens will occur only after review and approval by the HVTN, the IRB/EC of the researcher requesting the specimens, and the CRS's IRBs/ECs if required.

The protocol sample informed consent form is written so that the participant either explicitly allows or does not allow their samples to be used in other research when they sign the form. Participants who initially agree to other use of their samples may rescind their approval once they enter the study; such participants will remain in this study and their samples will only be used for the studies described in this protocol. If a participant decides against allowing other research using his or her samples, or at any time rescinds prior approval for such other use, the study site investigator or designee must notify HVTN Regulatory Affairs in writing. In either case, HVTN Regulatory Affairs directs the HVTN Lab Program not to use samples from these participants for such other uses.

CRSs must notify HVTN Regulatory Affairs if institutional or local governmental requirements pose a conflict with or impose restrictions on other use of specimens.

11.12 Biohazard containment

As the transmission of HIV and other blood-borne pathogens can occur through contact with contaminated needles, blood, and blood products, appropriate precautions will be employed by all personnel in the drawing of blood and shipping and handling of all specimens for this study, as currently recommended by the CDC and the NIH or other applicable agencies.

All dangerous goods materials, including Biological Substances, Category A or Category B, must be transported according to instructions detailed in the International Air Transport Association Dangerous Goods Regulations.

12 Safety monitoring and safety review

12.1 Safety monitoring and oversight

12.1.1 HVTN 702 PSRT

The HVTN 702 PSRT is composed of the following members:

- DAIDS medical officer representative,
- Protocol chair and cochairs,
- Protocol Team leader,
- Core medical monitor,
- Clinical safety specialist, and
- Regional medical liaison (RML).

The clinician members of HVTN 702 PSRT are responsible for decisions related to participant safety.

A medical officer from an in-country organization designated by the study sponsor will also participate in the PSRT.

The Protocol Team clinic coordinator, project manager, vaccine developer representative, clinical trial manager, and others may also be included in HVTN 702 PSRT meetings.

12.1.2 NIAID DSMB

The NIAID DSMB assesses the effects of the study vaccine during the trial, provides other monitoring as described in Sections 6.5, 6.9.3, and 13.1.4, and may give advice to the HVTN 702 OG.

As of the 23 January 2020 DSMB meeting, the DSMB oversight of the study is complete.

12.1.3 SDMC roles and responsibilities in safety monitoring

The roles and responsibilities of the SDMC in relation to safety monitoring include:

- Maintaining a central database management system for HVTN clinical data;

- Providing reports of clinical data to appropriate groups such as the HVTN 702 PSRT and NIAID DSMB;

12.1.4 HVTN Core roles and responsibilities in safety monitoring

- Daily monitoring of clinical data for events that meet immediate HVTN 702 PSRT notification criteria (Section 12.3);
- Notifying HVTN CRSs and other groups when safety pauses are instituted and lifted (Section 12.3);
- Querying HVTN CRSs for additional information regarding reported clinical data; and
- Providing support to the HVTN 702 PSRT.

12.2 Safety reporting

12.2.1 Submission of safety forms to SDMC

Sites must submit all safety forms (eg, reactogenicity, AE, concomitant medications) before the end of the next business day after receiving the information. The forms should not be held in anticipation of additional information at a later date. If additional information is received at a later date, the forms should be updated and resubmitted before the end of the next business day after receiving the new information.

12.2.2 AE reporting

An AE is any untoward medical occurrence in a clinical investigation participant administered a study product/procedure(s) and which does not necessarily have a causal relationship with this treatment. An AE can therefore be any unfavorable and unintended sign (including an abnormal laboratory finding), symptom, or disease temporally associated with the use of an investigational study product/procedure(s), whether or not related to the investigational study product/procedure(s). All AEs are graded according to the *Division of AIDS Table for Grading the Severity of Adult and Pediatric Adverse Events*, Corrected Version 2.1 dated July 2017, available at https://rsc.niaid.nih.gov/clinical-research-sites/daids-adverse-event-grading-tables, except that:

- Unintentional weight loss is required to be reported as an AE only if it is considered to be potentially deleterious to the participant's health (HVTN 702 SSP);
- Injection Site Erythema or Redness and Injection Site Induration or Swelling will not consider interference with usual social and functional activities such that:

- Grade 1 is: 2.5 to < 5 cm in diameter OR 6.25 to < 25 cm^2 surface area;
- Grade 2 is: ≥ 5 to < 10 cm in diameter OR ≥ 25 to < 100 cm^2 surface area;
- Grade 3 is: ≥ 10 cm in diameter OR ≥ 100 cm2 surface area OR Ulceration OR Secondary infection OR Phlebitis OR Sterile abscess OR Drainage;
- Grade 4 is: Potentially life-threatening consequences (eg, abscess, exfoliative dermatitis, necrosis involving dermis or deeper tissue);

In addition, clarifying footnote 16 (page 21) in Version 2.1 of the AE Grading Table, for transgender participants ≥ 13 years of age who have been on feminizing hormone therapy for more than 6 consecutive months, grade hemoglobin based on the female sex at birth hemoglobin lab values. For transgender participants ≥13 years of age who have been on masculinizing hormone therapy for more than 6 consecutive months, grade hemoglobin based on the male sex at birth hemoglobin lab values.

Unsolicited AEs will be collected over 30 days post each vaccination visit. All collected AEs are reported to the SDMC on the appropriate CRF. Clinic staff should evaluate every AE to determine if (1) the AE meets the requirements for expedited reporting to the study sponsor (Section 12.2.3), (2) if the AE meets the criteria for immediate notification to HVTN Core (Section 12.3), and (3) if the AE is a potential immune-mediated disease that may be listed as an AESI. A sample list of AESI is provided in Appendix J. Attribution with regard to relationship to study product will be reported for all AEs.

Certain AEs will be collected and reported throughout the entire study:

- SAEs/AEs requiring expedited reporting to DAIDS (EAEs),
- AESIs
- AEs of STIs
- New chronic medical conditions (defined as a new onset or exacerbation of medical condition requiring 2 or more visits to a medical provider during a period of at least 30 days). and
- AEs leading to early participant withdrawal or early discontinuation of study product(s) administration.

In addition, site investigators are required to submit AE information in accordance with IRB/EC and any applicable RE requirements.

12.2.3 Expedited reporting of adverse events

Requirements, definitions and methods for expedited reporting of AEs to the study sponsor are outlined in Version 2.0 (January 2010) of the *Manual for Expedited Reporting of Adverse Events to DAIDS* (DAIDS EAE Manual), which is available on the RSC website at https://rsc.niaid.nih.gov/clinical-research-sites/manual-expedited-reporting-adverse-events-daids. The SAE Reporting Category will be used for this study.

The DAIDS Adverse Event Reporting System (DAERS) must be used for expedited AE reporting to DAIDS. In the event of system outages or technical difficulties, expedited AE reports may be submitted via the DAIDS EAE Form. This form is available at https://rsc.niaid.nih.gov/clinical-research-sites/paper-eae-reporting.

For questions about DAERS, please contact NIAID CRMS Support at CRMSsupport@niaid.nih.gov. Please note that site queries may also be sent from within the DAERS application itself.

For questions about expedited AE reporting, please contact the RSC (DAIDSRSCSafetyOffice@tech-res.com).

An AE is considered to be an SAE according to MCC (now SAHPRA) guidelines (*Safety Reporting During Clinical Trials in South Africa*, https://www.sahpra.org.za/wp-content/uploads/2020/02/2_Safety-Reporting-during-Clinical-Trial_Nov19_v3-1.pdf), if it:

- results in death,
- is life-threatening,
- requires patient hospitalisation or prolongation of existing hospitalisation,
- results in persistent or significant disability/incapacity,
- is a congenital anomaly/birth defect, or
- is a medically important event or reaction.

The term “life-threatening” in the definition of “serious” refers to an event in which the patient was at risk of death at the time of the reaction/event. It does not refer to an event, which, hypothetically, might have caused death, if it were more severe.

Medical and scientific judgment should be exercised when deciding whether other situations are serious or not. Such instances could include medical events that may not be immediately life-threatening or result in death or hospitalization, but which may jeopardize the patient or may require intervention to prevent one of

the other outcomes listed in the definition above. Examples include blood dyscrasias or convulsions not resulting in hospitalization, or development of drug dependency or drug abuse.

The sponsor or designee(s) prepares and files expedited reports to appropriate regulatory authorities and ECs within the timelines required by the South African MCC (now SAHPRA) guidelines, which are detailed in *Safety Reporting During Clinical Trials in South Africa*, Version 3, November 2019.

Any SAE that is considered unexpected and for which the contribution of the study products cannot be ruled out will qualify for expedited reporting to the SAHPRA. The expedited reporting period for this study comprises the entire study period for each individual participant (from study enrolment until study completion or discontinuation from the study).

The study products for which expedited reporting are required are:

- ALVAC-HIV (vCP2438)
- Placebo for ALVAC-HIV (vCP2438)
- Bivalent Subtype C gp120/MF59
- Placebo for Bivalent Subtype C gp120/MF59

After a participant's final scheduled study visit, only SUSARs as defined in Version 2.0 of the DAIDS EAE Manual must be reported to DAIDS, if the study staff become aware of the events. All AESIs will be considered "unexpected", and, if deemed related to the study products, will be reported as SUSARs, if applicable.

12.3 AEs requiring immediate PSRT notification and prompt PSRT AE review

Prior to study participants being unblinded to their treatment assignments, certain events require immediate telephone notification by the site to the HVTN Core safety staff. Telephone numbers and email addresses for contacting HVTN Core safety staff are found on the Protocol home page on the HVTN Members' site (https://members.hvtn.org/protocols/hvtn702/SitePages/Home.aspx). For the events described in this section, the site will also email and fax forms immediately.

The events which require immediate telephone notification by the site to the HVTN Core 24 hour safety phone, include:

- Any SAE described by the study site as related to the study vaccination

HVTN Core then immediately notifies the PSRT via email or phone. The Protocol Chair or Co-chair then decides how to review the event (via email or conference call). The PSRT will convene within 24 hours and decide whether the event necessitates a pause in further injections. If the team cannot convene to review the event within 24 hours, the medical officer or protocol chair will make the final decision.

The events which require immediate telephone notification by the site to the RML or CSS include:

- any Grade 4 objective local or systemic reactogenicity signs/symptom, or
- Grade 4 AE related to study product, or
- any Grade 5 event

The RML or CSS notifies the HVTN 702 PSRT as soon as possible during working hours (South Africa Standard time or US Pacific Time, respectively)—or, if the information was received during off hours, by the morning of the next work day. If a prompt HVTN 702 PSRT AE review cannot be completed within 72 hours of notification (excluding weekends and US federal holidays), the medical officer or protocol chair will make the final determination as to whether the event necessitates a pause in further injections.

Subjective reactogenicity symptoms (injection site pain, tenderness, fatigue/malaise, myalgia, arthralgia, chills, headache, and nausea) do not require immediate telephone notification by the site to HVTN Core. Objective reactogenicity signs include injection site erythema/redness, induration/swelling, vomiting, and fever.

If the trial is placed on safety pause, all enrollment and vaccination with the product related to the event that triggered the pause will be held until further notice.

If the study is stopped early and CRSs are unblinded, immediate telephone notification is not required and the PSRT will routinely review AE reports.

For all safety pauses, HVTN Core notifies the HVTN 702 PSRT, HVTN Regulatory Affairs, DAIDS Pharmaceutical Affairs Branch (PAB), DAIDS Regulatory Affairs Branch (RAB), DAIDS Safety and Pharmacovigilance Team (SPT), and participating HVTN CRSs.

Once a trial is paused, the HVTN 702 PSRT reviews safety data and decides whether the pause can be lifted or permanent discontinuation of vaccination is appropriate, consulting the DSMB if necessary. HVTN Core notifies the participating HVTN CRSs, HVTN Regulatory Affairs, DAIDS PAB, DAIDS RAB, and DAIDS SPT of the decision regarding resumption or discontinuation of

study vaccinations. Based on the HVTN 702 PSRT assessment, the trial sponsor or designee(s) notifies the SAHPRA as needed.

The HVTN requires that each CRS submit to its IRB/EC protocol-related safety information (such as IND safety reports, notification of unplanned safety pauses). CRSs must also follow all applicable RE reporting requirements.

In addition, all other AEs are reviewed routinely by the HVTN 702 PSRT (Section 12.4.2).

12.4 Review of cumulative safety data

Routine safety review occurs at the start of enrollment and then throughout the study.

Reviews proceed from a standardized set of protocol-specific safety data reports. These reports are produced by the SDMC and include queries to the HVTN CRSs. Events are tracked by internal reports until resolution.

12.4.1 Daily review

Blinded daily safety reviews are routinely conducted by HVTN Core for events that meet immediate HVTN 702 PSRT notification criteria, as described in Section 12.3.

12.4.2 Weekly review

During the injection phase of the trial, the HVTN 702 PSRT reviews clinical safety reports on a weekly basis and conducts calls to review the data as appropriate. After the 2-week-post-final-vaccination visits are completed, less frequent reporting and safety reviews may be conducted at the discretion of the HVTN 702 PSRT. HVTN Core reviews reports of all reported AEs. Events identified during the review that are considered questionable, inconsistent, or unexplained are referred to the HVTN CRS clinic coordinator for verification.

12.4.3 DSMB review of cumulative safety data

The DSMB will periodically review accumulating unblinded safety data by group. Prior to each meeting, the SDMC will provide the DSMB with data as described in Section 6.5. Reports will be cumulative, generated from an up-to-date data file.

Based upon the reports, the DSMB will determine whether to recommend that the study should be continued, modified, or stopped for safety reasons.

As of the 23 January 2020 DSMB meeting, DSMB oversight of the study is complete.

12.5 Study termination

This study may be terminated early by NIAID upon recommendation by the DSMB, the SAHPRA, NIH, Office for Human Research Protections (OHRP), or vaccine developer(s). In addition, the conduct of this study at an individual HVTN CRS may be terminated by the determination of the IRB/EC and any applicable RE.

13 Protocol conduct

This protocol and all actions and activities connected with it will be conducted in compliance with the principles of GCP (ICHe6), and according to DAIDS and HVTN policies and procedures as specified in the *HVTN Manual of Operations* and DAIDS Clinical Research Policies and Standard Procedures Documents, including procedures for the following:

- Protocol registration, activation, and implementation;
- Informed consent, screening, and enrollment;
- Study participant reimbursement;
- Clinical and safety assessments;
- Safety monitoring and reporting;
- Data collection, documentation, transfer, and storage;
- Participant confidentiality;
- Study follow-up and close-out;
- Unblinding of staff and participants;
- Quality control;
- Protocol monitoring and compliance;
- Advocacy and assistance to participants regarding negative social impacts associated with the vaccine trial;
- Risk reduction counseling;
- Specimen collection, processing, and analysis;
- Ancillary studies, and
- Destruction of specimens.

Any policies or procedures that vary from DAIDS and HVTN standards or require additional instructions (eg, instructions for randomization specific to this study) will be described in the HVTN 702 SSP.

13.1 Protocol governance

13.1.1 Protocol Team

The Protocol Team will be responsible for administrative oversight of the study, provides the overall operational direction for the trial, and is responsible for the conduct of the trial according to the highest scientific and ethical standards, as well as approving revisions and amendments to the protocol. The Protocol Team will remain blinded to the treatment group assignment of individual participants during the course of the study.

13.1.2 PSRT

The PSRT will review all clinical and laboratory safety data during the course of the study as described in Section 12.4.

13.1.3 Oversight Group (OG)

The OG provides the overall scientific direction for the trial, and will receive and decide on any recommendations made by the DSMB, including stopping the study. The OG must approve all scientific reports concerning the main findings of the trial.

13.1.4 NIAID DSMB

The NIAID DSMB assesses the effects of the study vaccine during the trial and may give advice to the OG. The DSMB membership for this trial will include representatives from South Africa and is the same DSMB constituted for safety oversight for HVTN 100. With the exception of an unblinded statistician, the members of the committee are independent of the sponsor DAIDS, the HVTN and clinical investigators participating in this trial, will not have any other involvement in the study, and will not have any relation to study participants. The DSMB will monitor the trial for evidence of beneficial or adverse effects of the study vaccine using the guidelines proposed by the protocol. The DSMB may recommend any steps to ensure the safety of study participants and the integrity of the trial. Furthermore, it may recommend that the trial be terminated or that specific groups be withdrawn from the study, if any subgroup manifests serious or widespread side effects. To guarantee the unrestricted performance of its task, the DSMB may receive the individual study morbidity and mortality data from the unblinded statistician.

The DSMB will be informed immediately by an independent statistician if the pre-specified stopping boundary is met, indicating that the vaccine causes harm by increasing the rate of HIV acquisition (Section 6.5.1). In addition, the DSMB will monitor the study for futility to detect vaccine activity (Section 6.5.2), for high vaccine efficacy (Section 6.5.3), and for operational futility, defined as an unacceptably low rate of HIV-1 infections and by other measures of under-

performance (Section 6.5.5). Semiannual DSMB meetings will be held for monitoring operational futility.

Should ongoing clinical trials of HIV prevention modalities produce results having implications for recruitment or endpoint enrollment in HVTN 702, those results will be reported to the DSMB, whose recommendations will inform consultations with applicable experts and stakeholders regarding possible changes in trial design and conduct.

As of the 23 January 2020 DSMB meeting, DSMB oversight of the study is complete.

13.2 Social impacts

Participants in this study risk experiencing discrimination or other personal problems, resulting from the study participation itself or from the development of VISP. The HVTN CRS is obliged to provide advocacy for and assistance to participants regarding these negative social impacts associated with the vaccine trial. If HVTN CRS staff have questions regarding ways to assist a participant dealing with a social impact, a designated NIAID or HVTN Core representative can be contacted.

Social harms are tabulated by the SDMC and are subjected to descriptive analysis. The goal is to reduce their incidence and enhance the ability of study staff to mitigate them when possible.

Summary tables of social impact events will be generated weekly, and made available for review by the protocol chairs, protocol team leader, and the designated NIAID representative.

13.3 Compliance with NIH guidelines for research involving products containing recombinant DNA

Because this study is evaluating products containing recombinant DNA, it must comply with regulations set forth in the NIH's *Guidelines for Research Involving Recombinant or Synthetic Nucleic Acid Molecules.* Information about the study must be submitted to site Institutional Biosafety Committees (IBC) and must be approved before participants are enrolled at the site. Investigators at each site are responsible for obtaining IBC approval per NIH guideline *section IV-B07-a-(1).* IBC review and approval must be documented by the investigator and submitted as part of initial protocol registration for this trial. If this protocol is amended, investigators should follow the requirements of their IBC.

13.4 Specific regulatory considerations for Republic of South Africa

The Republic of South Africa has laws regarding the use, manufacture, importation, and experimentation of products which are genetically modified. These are contained in the Genetically Modified Organism (GMO) Act 15 of 1997, administered by the South African National Department of Agriculture, Pretoria. The Registrar of GMO shall be consulted on all formal developments relating to this protocol and clinical trial, and as required, a formal application will be made to the Registrar of GMO to review the HVTN 702 clinical trial, to obtain approval for the proposed clinical trial and for the importation of the study products.

13.5 Emergency communication with study participants

As in all clinical research, this study may generate a need to reach participants quickly to avoid imminent harm, or to report study findings that may otherwise concern their health or welfare.

When such communication is needed, the CRS will request that its IRB/EC and any applicable RE expedite review of the message. If this review cannot be completed in a timeframe consistent with the urgency of the required communication, the site should contact the participant first, and then notify the IRB/EC and any applicable RE of the matter as soon as possible.

14 Version history

The Protocol Team may modify the original version of the protocol. Modifications are made to HVTN protocols via clarification memos, letters of amendment, or full protocol amendments.

The version history of, and modifications to, Protocol HVTN 702 are described below.

Protocol history and modifications

Date: May 20, 2020

Protocol version: Version 4.0
Protocol modification: Full Protocol Amendment 3

Item 1	Boxed text added following title page: Vaccinations stopped, visit schedule and study procedures revised, consent addendum added
Item 2	Updated in Section 3, *Overview*: Participants, duration per participant, and estimated total study duration
Item 3	Updated in Section 3.1, *Protocol team*: Membership
Item 4	Revised in Section 5.1: *Primary objectives and endpoints*
Item 5	Revised in Section 5.2: Secondary objective 4
Item 6	Updated in Sections 5.2, 5.3, 11.9, and 15: Lab Center assay portfolio
Item 7	Clarified in Section 6.5, 12, and 13: End of DSMB trial oversight
Item 8	Updated in Section 6.5.10: Trial duration
Item 9	Added in Section 6.9.5.1: Analysis strategy for primary objective 3
Item 10	Added in Sections 6.9.5.2 and 6.9.5.5: Qualifications to planned analyses
Item 11	Added in Section 9.1.2, *Protocol-specific consent forms*: Post-unblinding addendum to sample informed consent form
Item 12	Added to titles of Section 9.3 and 9.4: "pre-unblinding"
Item 13	Added: Section 9.4.1, *Follow-up visits (post-unblinding)*
Item 14	Added in Section 9.7, *HIV counseling and testing*: Instructions for post-unblinding HIV testing
Item 15	Revised in Section 9.8, *Pregnancy prevention assessment*: Time period for documentation of participant contraceptive use
Item 16	Updated in Section 9.11, *Early termination visit*: Procedures
Item 17	Updated in Section 11, *Laboratory*: Assays and sample shipping
Item 18	Updated in Sections 12 and 15: Safety reporting guidance URLs

Item 19 Added: Appendix K, *Sample addendum to informed consent form (post-unblinding)*

Item 20 Added: Appendix L, *Tables of procedures (for addendum to sample informed consent form)*

Item 21 Added: Appendix M, *Schedule 3: Follow-up laboratory procedures for HIV-uninfected participants (post-unblinding)*

Item 22 Added: Appendix N, *Schedule 3: Follow-up procedures at HVTN CRS for HIV-uninfected participants (post-unblinding)*

Item 23 Revised per Clarification Memo 1 to Version 3.0: Procedure timing and regulator access to participant records

Item 24 Revised per Clarification Memo 2 to Version 3.0: DBS timing, Visit 12 timepoint, and SAHPRA update

Item 25 Clarified per Clarification Memo 3 to Version 3.0: Questionnaires at early termination visit

Item 26 Clarified per Clarification Memo 4 to Version 3.0: Missed visit documentation

Item 27 Clarified per Clarification Memo 5 to Version 3.0: Clinic visits

Item 28 Version history updated in Section 14

Item 29 Cross-references and literature citations updated and minor errors corrected

Date: April 6, 2020

Protocol version: Version 3.0
Protocol modification: Clarification Memo 5

Item 1 Clarified throughout protocol: “Clinic visits”

Date: March 19, 2019

Protocol version: Version 3.0
Protocol modification: Clarification Memo 4

Item 1 Clarified in Section 9.10, *Visit windows and missed visits*: Procedures for documenting out-of-window and missed visits

Date: July 23, 2018

Protocol version: Version 3.0
Protocol modification: Clarification Memo 3

Item 1 Clarified in Section 9.11: Questionnaires recommended at early termination visit

Date: March 21, 2018

Protocol version: Version 3.0
Protocol modification: Clarification Memo 2

Item 1 Clarified in Section 9.3 and Appendix H: DBS blood drawing timing

Item 2 Corrected in Appendix H: Visit 12 timepoint

Item 3 Updated in Sections 12 and 16, Appendices A and D: MCC now SAHPRA

Date: January 19, 2018

Protocol version: Version 3.0
Protocol modification: Clarification Memo 1

Item 1 Corrected: "Transmission risk reduction counseling" at Visit 31 in Appendix E and Appendix I

Item 2 Added in Appendices A and D: Broad regulatory agency access to participant study records

Date: December 7, 2017

Protocol version: Version 2.0
Protocol modification: Clarification Memo 3

Item 1 Corrected: Appendix F, *Schedule 1: Laboratory procedures for HIV-uninfected participants*

Date: October 5, 2017

Protocol version: Version 3.0
Protocol modification: Full Protocol Amendment 2

Item 1 Added: Month 18 booster vaccination

Item 2 Revised in Sections 3, 6.4.1, 6.9.5.2, and Appendix A: Study population sex-at-birth balance

Item 3 Updated in Section 3.1, *Protocol team*: Team membership, roles, and institutional affiliations

Item 4 Updated and corrected in Section 4.1, *Burden of HIV and rationale for HIV vaccine development* and Section 17, *Literature cited*: Literature citations

Item 5 Clarified in Section 4.4.2, *Manufacturing and formulation*: Protein storage temperature and formulation characteristics

Item 6 Updated in Section 4.9.6, *HVTN 100*: Amendment description

Item 7 Revised in Sections 6.5.5 and 6.5.6: Statistical analysis for operational feasibility assessment

Item 8 Revised in Section 6.5.9, *Power for secondary VE analysis*: Study power under different HIV incidence and gender balance assumptions

Item 9 Added in Section 6.9.5.2. *Secondary vaccine efficacy analyses: HIV-1 acquisition*: Vaccine efficacy in a population with equal gender distribution

Item 10 Clarified in Section 7, *Selection and withdrawal of participants*: Eligibility determination

Item 11 Clarified in Section 8, *Study product preparation and administration*: Regimen, syringe labeling, and blinding

Item 12 Added in Section 9.3 and Appendices F and H: Hemoglobin testing

Item 13 Clarified in Section 9.8 and Appendix H: *Pregnancy prevention assessment*

Item 14 Updated and corrected in Sections 9.7.1, 11.1, and 15: Laboratory reference documents

Item 15 Clarified in Section 9.8 and footnote to Appendix H: Duration of contraception use requirement

Item 16 Updated in Sections 9.9, 12.2.2, and 15: DAIDS AE grading table version

Item 17 Clarified in Section 9.11 and Appendix H: Procedures at early termination visits

Item 18 Updated in Section 11.4: Humoral endpoint assays

Item 19 Updated in Sections 9.9, 12.2.2, and 15: DAIDS AE grading table and exceptions

Item 20 Updated in Section 12, Appendix A, and Section 15: Document references and URLs

Item 21 Updated in Section 16: Acronyms and abbreviations

Item 22 Updated in Appendix A, *Sample informed consent form*:

Item 23 Updated in Appendix A, *Sample informed consent form*: Sample testing, and stool sampling

Item 24 Updated in Appendix A and Appendix D: Use of extra samples, contact information, permissions

Item 25 Corrected in Appendix E, *Tables of procedures (for sample informed consent form)*:

Item 26 Updated in footnote to Appendix F: HVTN laboratories

Item 27 Revised in footnotes to Appendices G and I: Subsequent HIV-testing redraw visits

Item 28 Corrected in Appendix H: Consent procedure

Item 29 Restored in Appendix H: Behavioral risk assessment questionnaire timepoints

Item 30 Added in Appendices H and I: Footnote clarifying stool sampling data collection

Item 31 Clarified in Appendix H footnotes: Blood draw windows at vaccination visits, HIV test results provision, and STI testing timepoints

Item 32 Clarified in Appendix I, *Schedule 2: Procedures at HVTN CRS for HIV-infected participants*: Procedures at visit #.X

Item 33 Added as Appendix K: Protocol signature page

Item 34 Corrected: Oversight Group (OG) terminology

Item 35 Corrected, Updated, and Clarified in Clarification Memo 1 to protocol Version 2.0: Appendix G procedures table, assay location and lab listings, and HIV-testing redraw visit blood draws

Item 36 Added via Clarification Memo 2 to protocol Version 2.0: Backup HIV diagnostic testing laboratory

Item 37 Corrected: Typographical errors and stylistic inconsistencies

Date: August 16, 2017

Protocol version: Version 2.0
Protocol modification: Clarification Memo 2

Item 1 Added in Section 3, *Overview*, and Appendices F and G: Backup laboratory for HIV diagnostics

Date: February 27, 2017

Protocol version: Version 2.0
Protocol modification: Clarification Memo 1

Item 1 Corrected: Appendix G, *Schedule 2: Laboratory procedures for HIV-infected participants*

Item 2 Updated in Appendices F and G: Assay locations and HVTN laboratory listings

Item 3 Clarified in footnotes to Appendices G and I: Blood draws required at extra HIV-testing redraw visits

Date: December 8, 2016

Protocol version: Version 2.0
Protocol modification: Full Protocol Amendment 1

Item 1 Clarified in Section 3, *Overview*: Duration per participant

Item 2 Updated in Section 3.1: Protocol Team

Item 3 Corrected in Section 4.4.4, *Bivalent Subtype C gp120/MF59 for injection*: Buffer quantities

Item 4 Corrected in Section 4.5.2: *Assumptions regarding HIV-1 incidence*

Item 5 Added: Section 4.6, *Combination prevention of HIV acquisition*

Item 6 Updated in Sections 4.9 and 4.10: HVTN 100 clinical experience

Item 7 Added: Adverse events of special interest (AESIs)

Item 8 Added: Optional stool sampling and gut microbiome analysis

Item 9 Added: PrEP use monitoring using dried blood spots

Item 10 Added: STI testing

Item 11 Removed from clinical and laboratory procedures: Urine testing

Item 12 Clarified in Section 5, O*bjectives and endpoints*: Primary endpoint adjudication and effect modifications

Item 13 Added in Section 5.3, *Exploratory objectives*: Comparative correlates of risk analysis

Item 14 Updated and clarified in Section 6.5: Trial monitoring

Item 15 Clarified in Section 6.9: Statistical analyses

Item 16 Clarified in Section 6.7, *Randomization of treatment assignments*:

Item 17 Removed in Section 7.1, *Inclusion criteria* and Appendices B and C: Barrier contraception requirement and partner vasectomy as contraception

Item 18 Clarified in Section 7.3.3, *Discontinuing vaccination for a participant*: Exception for specified injection-site reactions with PSRT approval

Item 19 Clarified in Section 9, *Clinical procedures*: Source documents

Item 20 Clarified in Section 9, *Clinical procedures*: Reactogenicity assessments

Item 21 Removed in Section 9, *Clinical procedures* and Appendix H, *Schedule 1: Procedures at HVTN CRS for HIV-uninfected participants*: Contraceptive assessment

Item 22 Removed in Section 9.2, *Pre-enrollment procedures*: Required recording of generic names for concomitant medications

Item 23 Clarified in Section 12.2, *Safety reporting*: AE/SAE and SUSAR reporting

Item 24 Updated in Section 12.3: HVTN 702 protocol home page URL

Item 25 Updated: Section 14, *Version history*

Item 26 Updated in Section 15, *Document references (other than literature citations)*: Document URLs

Item 27 Updated in Appendix A, *Sample informed consent form*: Risks of study vaccines and description of sample testing

Item 28 Added to Appendix A, *Sample informed consent form*: Information on oral PrEP

Item 29 Updated in Appendix A, *Sample informed consent form*: Sample testing language

Item 30 Clarified in Appendix A, *Sample informed consent form*: Funder access to study information

Item 31 Added to Appendix A, *Sample informed consent form*: MCC contact information

Item 32 Clarified in Appendix E, *Tables of procedures (for sample informed consent form)*: Questions/questionnaire

Item 33 Revised in Appendix F, *Schedule 1: Laboratory procedures for HIV-uninfected participants*: Blood draw volumes

Item 34 Revised in Appendices G and I: *Laboratory and CRS procedures for HIV-infected participants*

Item 35 Clarified in Appendix H, *Schedule 1: Procedures at HVTN CRS for HIV-uninfected participants*: Timing of final visit procedures

Item 36 Revised in Appendix H, *Schedule 1: Procedures at HVTN CRS for HIV-uninfected participants*: Frequency of behavioral risk assessment questionnaire

Item 37 Revised in Appendix H, *Schedule 1: Procedures at HVTN CRS for HIV-uninfected participants*: Window for blood draws prior to vaccination

Item 38 Revised in Appendix F and G: Laboratory designations

Item 39 Clarified in footnotes to Appendices F and H: Pregnancy testing

Item 40 Updated via Clarification Memo 1: Study product information and reactogenicity symptoms

Item 41 Minor typographical, grammatical, stylistic, and formatting errors have been corrected and cross-references have been updated throughout the protocol document

Date: September 16, 2016

Protocol version: Version 1.0
Protocol modification: Clarification Memo 1

Item 1 Clarified in Section 4.3.3, *Manufacturing*, Section 8.2, *Study product formulation*, and Section 8.5, *Acquisition of study products*: ALVAC-HIV (vCP2438) manufacturers, labeling, and study product acquisition

Item 2 Clarified in Section 8.3, *Preparation of study products*: Diluent vials, study product stability, and preparation

Item 3 Clarified in Section 12.3, *AEs requiring immediate PSRT notification and prompt PSRT AE review*: Subjective and objective reactogenicity symptoms

Date: December 23, 2015

Protocol version: 1.0
Protocol modification: Original protocol

15 Document references (other than literature citations)

Other documents referred to in this protocol, and containing information relevant to the conduct of this study, include:

- Assessment of Understanding. Accessible through the HVTN protocol-specific website.
- ABPI. *Guidelines for Phase 1 Clinical Trials 2012 Edition* Available at http://www.abpi.org.uk/our-work/library/guidelines/Pages/phase-1-trials-2012.aspx
- Current CDC Guidelines. *Revised Recommendations for HIV Testing of Adults, Adolescents, and Pregnant Women in Health-Care Settings*. Available at http://www.cdc.gov/mmwr/PDF/rr/rr5514.pdf
- Division of AIDS (DAIDS) Clinical Research Policies and Standard Procedures Documents. Available at https://www.niaid.nih.gov/research/daids-clinical-research-policies-standard-procedures
- *Division of AIDS Protocol Registration Manual*. Available at https://www.niaid.nih.gov/sites/default/files/prmanual.pdf
- Division of AIDS *Table for Grading the Severity of Adult and Pediatric Adverse Events*. Corrected Version 2.1 dated July 2017. Available at https://rsc.niaid.nih.gov/clinical-research-sites/daids-adverse-event-grading-tables
- *The Manual for Expedited Reporting of Adverse Events to DAIDS*. Version 2.0, January 2010. Available at http://rsc.tech-res.com/clinical-research-sites/safety-reporting/manual
- *Safety Reporting During Clinical Trials in South Africa.* Available at https://www.sahpra.org.za/wp-content/uploads/2020/02/1_SA-GCP-Guidelines_2nd-edition_2006-1.pdf
- *Guidelines for Good Practice in the Conduct of Clinical Trials with Human Participants in South Africa*. Available at http://www.mccza.com/documents/7c21ba77SA_GCP_guidelines_second_edition_2006.pdf
- Republic of South Africa: *National Contraception Clinical Guidelines*. December 2012. Available at http://www.mm3admin.co.za/documents/docmanager/3c53e82b-24f2-49e1-b997-5a35803be10a/00037761.pdf

- *Ethics in Health Research: Principles, Processes and Structures (Second edition, 2015)*, Department of Health, Republic of South Africa. Available at http://www.nhrec.org.za/docs/Documents/EthicsHealthResearchFinalAused.pdf

- *Declaration of Helsinki (last updated October 2013)*, World Medical Association. Available at http://www.wma.net/en/30publications/10policies/b3/

- Republic of South Africa, Medical Research Council: *Guidelines on Ethics for Medical Research: HIV Preventive Vaccine Research*. Available at http://www.mrc.ac.za//ethics/ethicsbook5.pdf

- South Africa Genetically Modified Organism (GMO) Act 15 of 1997 Available at http://www.saflii.org/za/legis/num_act/gmoa1997286/

- HVTN 702 Special Instructions. Accessible through the HVTN protocol-specific website.

- HVTN 702 Study Specific Procedures. Accessible through the HVTN protocol-specific website.

- HVTN 702 Site Lab Instructions. Accessible through the HVTN protocol-specific website.

- HVTN *Manual of Operations*. Accessible through the HVTN website.

- Dangerous Goods Regulations (updated annually), International Air Transport Association. Available for purchase at http://www.iata.org/publications/dgr/Pages/index.aspx

- Laboratory Center assay portfolio Available at http://www.hvtn.org/en/science.html

- International Conference on Harmonisation (ICH) E6 (R1), *Guideline for Good Clinical Practice*: Section 4.8, *Informed consent of trial subjects*. Available at http://www.ich.org/products/guidelines/efficacy/article/efficacy-guidelines.html

- *Participants' Bill of Rights and Responsibilities*. Accessible through the HVTN website.

- NIH *Guidelines for Research Involving Recombinant or Synthetic Nucleic Acid Molecules,* April 2016. Available at http://osp.od.nih.gov/sites/default/files/resources/NIH_Guidelines.pdf

- *NIH Policy on Reporting Race and Ethnicity Data: Subjects in Clinical Research*. Available at http://grants1.nih.gov/grants/guide/notice-files/NOT-OD-01-053.html

- Pharmacy Guidelines and Instructions for DAIDS Clinical Trials Networks, July 2008.

- *Requirements for Source Documentation in DAIDS Funded and/or Sponsored Clinical Trials*. Available at https://www.niaid.nih.gov/sites/default/files/documents/daids-sourcedocpolicy.pdf

- Title 21, Code of Federal Regulations, Part 50. Available at http://www.accessdata.fda.gov/scripts/cdrh/cfdocs/cfcfr/CFRSearch.cfm?CFRPart=50

- Title 45, Code of Federal Regulations, Part 46. Available at http://www.hhs.gov/ohrp/humansubjects/guidance/45cfr46.html

- Immunization Action Coalition, *Vaccines with Diluents: How to Use Them*. Available at http://www.immunize.org/catg.d/p3040.pdf

- US Centers for Disease Control and Prevention. Vaccine Storage & Handling Toolkit, June 2016. Available at http://www.cdc.gov/vaccines/hcp/admin/storage/toolkit/storage-handling-toolkit.pdf

See Section 17 for literature cited in the background and statistics sections of this protocol.

16 Acronyms and abbreviations

Ab	antibody
ADCC	antibody dependent cellular cytotoxicity
ADCVI	antibody dependent cellular viral inhibition
AE	adverse event
AESI	adverse event of special interest
AIDS	acquired immune deficiency syndrome
ALT	alanine aminotransferase
ART	antiretroviral therapy
ARV	antiretroviral
AVEG	AIDS Vaccine Evaluation Group
β-HCG	beta human chorionic gonadotropin
BAMA	binding antibody multiplex assay
BMI	body mass index
CAB	Community Advisory Board
$CCID_{50}$	cell culture infectious dose (50%)
CDC	US Centers for Disease Control and Prevention
CEB	chick embryo fibroblasts
CFR	Code of Federal Regulations
CHIL	Cape Town HVTN Immunology Laboratory
CHO	Chinese hamster ovary
CI	confidence intervals
CMIA	chemiluminescent microparticle immunoassay
CoR	correlate of risk
CPK	creatine phosphokinase
CRF	case report form
CRPMC	NIAID Clinical Research Products Management Center
CRS*	clinical research site
CTL	cytotoxic T lymphocyte
DAERS	DAIDS Adverse Event Reporting System
DAIDS	Division of AIDS (US NIH)
DBP	diastolic blood pressure
DHHS	US Department of Health and Human Services
DNA	deoxyribonucleic acid
DSMB	NIAID Data and Safety Monitoring Board
EAE	adverse events requiring expedited reporting to DAIDS
EC	Ethics Committee
EIA	enzyme immunoassay
ELISA	enzyme-linked immunosorbent assay

ELISpot	enzyme-linked immunospot
EU	European Union
FDA	US Food and Drug Administration
FHCRC	Fred Hutchinson Cancer Research Center
FIC	full immunization cohort
GCP	Good Clinical Practice
GLP	Good Laboratory Practice
GM	geometric mean
GMO	genetically modified organism
GPP	Good Participatory Practice
GSK	GlaxoSmithKline
HAART	highly active antiretroviral therapy
HBV	hepatitis B virus
HCl	hydrogen chloride
HCRISA	HVTN Centre for Research Institute of South Africa
HIV	human immunodeficiency virus
HLA	human leukocyte antigen
HMSL-NICD	HIV Sero-Molecular Laboratory, National Institute for Communicable Diseases
HVTN	HIV Vaccine Trials Network
IAC	Immunization Action Coalition
IB	Investigator's Brochure
IBC	Institutional Biosafety Committee
IC	immunogenicity cohort
ICH	International Conference on Harmonisation
ICS	intracellular cytokine staining
IFN-γ	interferon gamma
IgA	immunoglobulin A
IgG	immunoglobulin G
IM	intramuscular
IN	intranasal
IRB	Institutional Review Board
ISS	Istituto Superiore di Sanità (Rome)
IUD	intrauterine device
LC	Laboratory Center
MAR	missing at random
MCC	Medicines Control Council
MedDRA	Medical Dictionary for Regulatory Activities
MFI	mean fluorescent intensity

MIGEE	multiple imputation generalized estimating equation
MITT	modified intent-to-treat
MMR	measles, mumps, and rubella
MSM	men who have sex with men
MUVAPRED	Mucosal Vaccines for Poverty Related Diseases
nAb	neutralizing antibody
NaCl	sodium chloride
NHP	nonhuman primate
NIAID	National Institute of Allergy and Infectious Diseases (US NIH)
NICD	National Institute for Communicable Diseases
NIH	US National Institutes of Health
OG	Oversight Group
OHRP	US Office for Human Research Protections
OPV	oral polio vaccine
P5	Pox Protein Public Private Partnership
PAB	DAIDS Pharmaceutical Affairs Branch
PBMC	peripheral blood mononuclear cell
PCR	polymerase chain reaction
PEP	postexposure prophylaxis
PHRU	Perinatal HIV Research Unit
PLG	polylactide-co-glycolide (microparticle)
PP	per-protocol [cohort]
PrEP	pre-exposure prophylaxis
PI	Principal Investigator
PSRT	Protocol Safety Review Team
RAB	DAIDS Regulatory Affairs Branch
rcdf	reverse cumulative distribution function
RE	regulatory entity
REML	restricted maximum likelihood
RML	Regional Medical Liaison
RSA	Republic of South Africa
RSC	DAIDS Regulatory Support Center
SAAVI	South African AIDS Vaccine Initiative
SAE	serious adverse event
SAHPRA	South African Health Products Regulatory Authority
SAIL-NICD	South African Immunology Laboratory
SAP	statistical analysis plan
SBP	systolic blood pressure
SCHARP	Statistical Center for HIV/AIDS Research and Prevention

SDMC	statistical and data management center
SOC	Systems Organ Class
SPF	specific pathogen free
SPT	DAIDS Safety and Pharmacovigilance Team
SSP	study specific procedures
STI	sexually transmitted infection
SUSAR	suspected, unexpected serious adverse reaction
TB	tuberculosis
TG	transgender persons
TM	transmembrane
TNF-α	tumor necrosis factor alpha
UL	upper limit
USMHRP	US Military HIV Research Program
VE	vaccine efficacy
VISP	Vaccine induced seropositivity
VL	viral load

* CRSs were formerly referred to as HIV Vaccine Trial Units (HVTUs). Conversion to use of the term CRS is in process, and some HVTN documents may still refer to HVTUs.

17 Literature cited

1. UNAIDS. Ethical considerations in HIV preventive vaccine research. 2002 2002.

2. The National Commission for the Protection of Human Subjects of Biomedical and Behavioral Research. The Belmont Report: Ethical Principles and Guidelines for the Protection of Human Subjects of Research. 1979 4/18/1979.

3. Council for International Organizations of Medical Sciences (CIOMS). International ethical guidelines for biomedical research involving human subjects. Bull Med Ethics. 2002(182):17-23.

4. World Health Organization. Use of antiretroviral drugs for treating pregnant women and preventing HIV infection in infants: Programmatic update. 2012 2012. Report No.: WHO/HIV/2012.6.

5. Abdool Karim SS. An AIDS-free generation? Science. 2012;337(6091):133.

6. Baeten JM, Donnell D, Ndase P, Mugo NR, Campbell JD, Wangisi J, et al. Antiretroviral prophylaxis for HIV prevention in heterosexual men and women. N Engl J Med. 2012;367(5):399-410.

7. Grant RM, Lama JR, Anderson PL, McMahan V, Liu AY, Vargas L, et al. Preexposure chemoprophylaxis for HIV prevention in men who have sex with men. N Engl J Med. 2010;363(27):2587-99.

8. Thigpen MC, Kebaabetswe PM, Paxton LA, Smith DK, Rose CE, Segolodi TM, et al. Antiretroviral preexposure prophylaxis for heterosexual HIV transmission in Botswana. N Engl J Med. 2012;367(5):423-34.

9. Choopanya K, Martin M, Suntharasamai P, Sangkum U, Mock PA, Leethochawalit M, et al. Antiretroviral prophylaxis for HIV infection in injecting drug users in Bangkok, Thailand (the Bangkok Tenofovir Study): a randomised, double-blind, placebo-controlled phase 3 trial. Lancet. 2013;381(9883):2083-90.

10. UNAIDS. The GAP Report. 2014.

11. UNAIDS. Global AIDS Update. 2016. http://www.unaids.org/sites/default/files/media_asset/global-AIDS-update-2016_en.pdf.

12. UNAIDS. Prevention Gap Report. 2016. http://www.unaids.org/sies/default/files/media_asset/2016-prevention-gap-report_en.pdf.

13. Ramjee G, Daniels B. Women and HIV in Sub-Saharan Africa. AIDS Res Ther. 2013;10(1):30.

14. Shisana O, Rehle T, Simbayi L, Zuma K, Jooste S, Zungu N, et al. South African National HIV Prevalence, Incidence and Behaviour Survey, 2012. 2014.

15. UNAIDS. South Africa, HIV and AIDS estimates (2014) 2015 [updated 2015. Available from: http://www.unaids.org/en/regionscountries/countries/southafrica/]

16. AVERT. Treatment as Prevention (TASP) for HIV. 2017 [Available from: https://www.avert.org/professionals/hiv-programming/prevention/treatment-as-prevention]

17. WHO/UNAIDS. Voluntary medical male circumcision for HIV prevention 2012 [Available from: http://www.who.int/hiv/topics/malecircumcision/fact_sheet/en/]

18. Fauci AS, Marston HD. Ending AIDS--is an HIV vaccine necessary? N Engl J Med. 2014;370(6):495-8.

19. Rerks-Ngarm S, Pitisuttithum P, Nitayaphan S, Kaewkungwal J, Chiu J, Paris R, et al. Vaccination with ALVAC and AIDSVAX to prevent HIV-1 infection in Thailand. N Engl J Med. 2009;361(23):2209-20.

20. Robb ML, Rerks-Ngarm S, Nitayaphan S, Pitisuttithum P, Kaewkungwal J, Kunasol P, et al. Risk behaviour and time as covariates for efficacy of the HIV vaccine regimen ALVAC-HIV (vCP1521) and AIDSVAX B/E: a post-hoc analysis of the Thai phase 3 efficacy trial RV 144. Lancet Infect Dis. 2012;12(7):531-7.

21. Haynes BF, Gilbert PB, McElrath MJ, Zolla-Pazner S, Tomaras GD, Alam SM, et al. Immune-correlates analysis of an HIV-1 vaccine efficacy trial. N Engl J Med. 2012;366(14):1275-86.

22. Bredell H, Williamson C, Sonnenberg P, Martin DJ, Morris L. Genetic characterization of HIV type 1 from migrant workers in three South African gold mines. AIDS Res Hum Retroviruses. 1998;14(8):677-84.

23. Van Harmelen JH, Van der RE, Loubser AS, York D, Madurai S, Lyons S, et al. A predominantly HIV type 1 subtype C-restricted epidemic in South African urban populations. AIDS Res Hum Retroviruses. 1999;15(4):395-8.

24. Novitsky V, Smith UR, Gilbert P, McLane MF, Chigwedere P, Williamson C, et al. Human immunodeficiency virus type 1 subtype C molecular phylogeny: consensus sequence for an AIDS vaccine design? J Virol. 2002;76(11):5435-51.

25. Guevara H, Johnston E, Zijenah L, Tobaiwa O, Mason P, Contag C, et al. Prenatal transmission of subtype C HIV-1 in Zimbabwe: HIV-1 RNA and DNA in maternal and cord blood. J Acquir Immune Defic Syndr. 2000;25(5):390-7.

26. Sher R. HIV infection in South Africa, 1982-1988—a review. S Afr Med J. 1989;76(7):314-8.

27. Van Harmelen JH, Wood R, Lambrick M, Rybicki EP, Williamson AL, Williamson C. An association between HIV-1 subtypes and mode of transmission in Cape Town, South Africa. AIDS. 1997;11(1):81-7.

28. Peeters M, Toure-Kane C, Nkengasong JN. Genetic diversity of HIV in Africa: impact on diagnosis, treatment, vaccine development and trials. AIDS. 2003;17(18):2547-60.

29. McCormack GP, Glynn JR, Crampin AC, Sibande F, Mulawa D, Bliss L, et al. Early evolution of the human immunodeficiency virus type 1 subtype C epidemic in rural Malawi. J Virol. 2002;76(24):12890-9.

30. Jacobs GB, Loxton AG, Laten A, Robson B, van Rensburg EJ, Engelbrecht S. Emergence and diversity of different HIV-1 subtypes in South Africa, 2000-2001. J Med Virol. 2009;81(11):1852-9.

31. Romani B, Glashoff R, Engelbrecht S. Molecular and phylogenetic analysis of HIV type 1 vpr sequences of South African strains. AIDS Res Hum Retroviruses. 2009;25(3):357-62.

32. Jacobs GB, Laten A, van Rensburg EJ, Bodem J, Weissbrich B, Rethwilm A, et al. Phylogenetic diversity and low level antiretroviral resistance mutations in HIV type 1 treatment-naive patients from Cape Town, South Africa. AIDS Res Hum Retroviruses. 2008;24(7):1009-12.

33. Hemelaar J, Gouws E, Ghys PD, Osmanov S. Global and regional distribution of HIV-1 genetic subtypes and recombinants in 2004. AIDS. 2006;20(16):W13-W23.

34. UNAIDS. Report on the Global AIDS Epidemic. 2012. UNAIDS / JC2417E.

35. UNAIDS. Report on the Global AIDS Epidemic. 2010 2010. UNAIDS/10.11E | JC1958E.

36. WHO. Country Health Profile of South Africa-2006. 2010.

37. UNAIDS WHO. Epidemiological Fact Sheet on HIV and AIDS: Core data on epidemiology and response: South Africa. 2008.

38. AVERT. Children orphaned by HIV and AIDS. 2012 [Available from: http://www.avert.org/aids-orphans.htm#contentTable1]

39. Zolla-Pazner S, DeCamp A, Gilbert PB, Williams C, Yates NL, Williams WT, et al. Vaccine-Induced IgG Antibodies to V1V2 Regions of Multiple HIV-1 Subtypes Correlate with Decreased Risk of HIV-1 Infection. PLoS ONE. 2014;9(2):e87572.

40. Rolland M, Edlefsen PT, Larsen BB, Tovanabutra S, Sanders-Buell E, Hertz T, et al. Increased HIV-1 vaccine efficacy against viruses with genetic signatures in Env V2. Nature. 2012;490(7420):417-20.

41. Yates NL, Liao HX, Fong Y, DeCamp A, Vandergrift NA, Williams WT, et al. Vaccine-induced Env V1-V2 IgG3 correlates with lower HIV-1 infection risk and declines soon after vaccination. Sci Transl Med. 2014;6(228):228ra39.
