# Supplementary material for: Sexually transmitted infections amongst men who have sex with men (MSM) in South Africa
Source: PLOS Glob Public Health. 2023 Apr 5;3(4):e0001782. doi: 10.1371/journal.pgph.0001782 (PMC10075439; doi:10.1371/journal.pgph.0001782)
Supplement: Attachment — Submitted filename: Response to Reviewers.pdf [file pgph.0001782.s002.pdf]

Manuscript: PGPH-D-22-00629

Sexually transmitted infections amongst men who have sex with men (MSM) in South Africa
PLOS Global Public Health

<u>Dear EDITOR:</u>

Thank you for reviewing this manuscript. Please see below responses to your comments/suggestions

***Please amend your detailed Financial Disclosure statement. This is published with the article. It must therefore be completed in full sentences and contain the exact wording you wish to be published*.**
State the initials, alongside each funding source, of each author to receive each grant.

The financial disclosure statement has been amended. We added these statements, The funders had no role in study design, data collection and analysis, decision to publish, or preparation of the manuscript. The authors received no funding for this work.

In the online submission form, you indicated that "Availability of data and materials: The data that support the findings of this study are available on request from the corresponding author, RM. Data is currently not available to the public due to publication restrictions". All PLOS journals now require all data underlying the findings described in their manuscript to be freely available to other researchers, either 1. In a public repository, 2. Within the manuscript itself, or 3. Uploaded as supplementary information.

Thank you for clarifying the PLOS journal requirements.

The following comment has been added to Availability of data and materials: All relevant data used in this manuscript **will be made** available in a public repository or included in the Supplementary section.

Please provide separate figure files in .tif or .eps format and removed from the manuscript file.

Figure 1. has been removed from the manuscript and added to supplementary information in .tif format.

Figure 1: Estimated STI prevalence and 95% CIs over time among MSM participants who had data available at enrolment into HVTN 702.

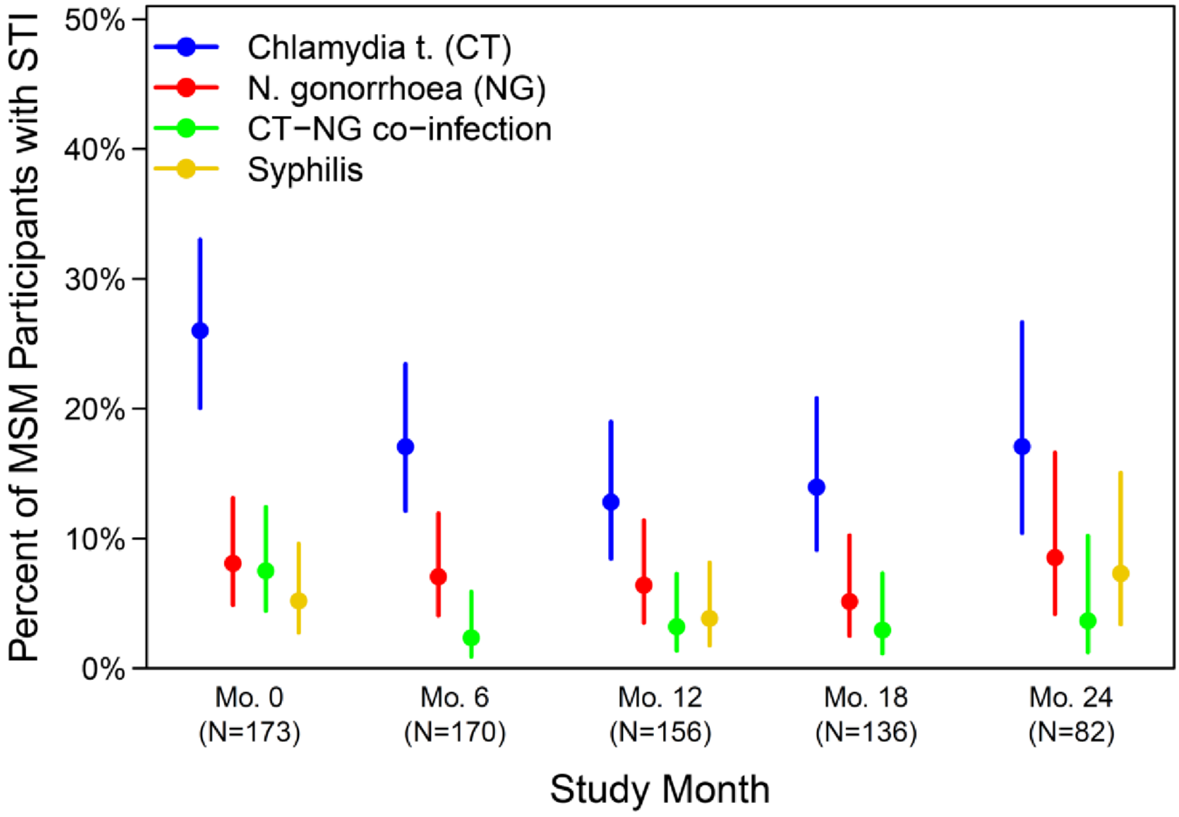

Dear Reviewer #1:

STI testing appears to have been done on rectal and urine samples (for men), but only one set of results is reported? Were these always concordant? This is listed as a limitation in the analysis, but surely it must be possible to compare in men which samples were positive? This must have been recorded?

Thank you for this question pertaining to the rectal/urine samples (for men).To clarify your statement rectal samples were collected in MSM, whilst urine samples were collected on both MSM and non-MSM. The rectal/urine tests in MSM were not always concordant.

We have added these statements to the discussion. For our analysis, CT and NG results among MSM were each considered positive if either the urine or rectal sample testing was positive. Including the specific anatomical site for the positive test was therefore not a requirement for this analysis. Urine testing for non-MSMs was regarded as sufficient for detection of STIs in this population.

***Very few details are given re: how the MSM were recruited. Did 702 use a recruiting procedure targeted at MSM, or did the overall strategy just happen to include some MSM? Obviously the MSM are much smaller, so the gender/sex comparisons, while certainly of interest, are limited in terms of MSM sample size and unknown generalizability*.**

In order to preserve study power to assess vaccine efficacy, while maintaining representation of both sexes, HVTN 702 was designed to enroll between 30-35% persons assigned male sex at birth. HVTN 702 did not have a specific allocation to enroll men by sexual orientation. The overall recruitment strategy to include persons at risk of HIV acquisition therefore happened to include some MSM.

Since a major part of the paper is comparing participants by gender & sex, it is an odd choice not to include the demographic, behavioral, and clinical data in Table 1. There is no way to know how these populations are comparable apart from sex & gender.

Thank you for this suggestion to include demographics for females and non-MSM males. We have added this information to Table 1 within the manuscript.

The hypothesis for why M0 and M6 would be different and the reason for a GEE between those 2 time points is unclear. Furthermore, why compare by sex & gender at M0 when STIs were presumably run on all 3 groups all throughout the trial? A GEE that compared the 3 groups at all of the time points would likely make more sense. This study cohort also provides an opportunity to measure incidence, but this does not appear to have been undertaken & it's unclear why not.

Formal statistical comparisons are made only for month 0 vs 6, where we expected to see a difference due to risk reduction counselling based on past HIV vaccine trial experience. GEE was used to compare these two time points, accounting for the correlation in the repeated measurements from the same participant. The main focus of the manuscript was to assess STI prevalence within MSMs over time and not to compare STIs between sex/gender groups. Incidence estimates are limited by the considerable missing data beyond month 6.

Why was a risk factor analysis not carried out for STI prevalence within MSM? Even an unadjusted table could help to profile who is at most risk within the MSM sub-group. The discussion contains a lot of mention of prior risk factor analyses, but it's unclear why these weren't repeated in this study?

Thank you for this comment. We have added Table 5 (see below) describing the baseline factors seen among MSMs with and without an STI at month 0.

Table 5: Risk factor analysis for MSM sub-population

	[1]MSM without STI (82)	[2]MSM with STI (91)	[3]p value
**Demographic characteristic**	N (%)	N (%)	
Age in years			0.010
≤ 24	48 (58.5)	70 (76.9)	
>24	34 (41.5)	21 (23.1)	
Race			0.049
**Black**	78 (95.1)	91 (100.0)	
**Coloured/Mixed**	4 (4.9)	0 (0.0)	
Gender Identity			1.00
**Male**	78 (95.1)	86 (94.5)	
**Transgender female**	4 (4.9)	5 (5.5)	
Sexual Orientation			0.63
**Homosexual**	53 (64.6)	63 (69.2)	
**Bisexual**	29 (35.4)	28 (30.8)	
Marital Status			0.58
**Married/Main partner**	63 (76.8)	65 (76.8)	
**No main partner**	18 (22.0)	24 (26.4)	
**Unknown**	1 (1.2)	2 (2.2)	
Lives with Spouse/Main			0.69
Partner	8 (9.8)	10 (11.0)	
**Yes**	55 (67.1)	55 (60.4)	
**No**	1 (1.2)	2 (2.2)	
**Unknown**	18 (22.0)	24 (26.4)	
**N/A – [No main partner]**			
Highest education			0.24
completed	12 (14.6)	22 (24.2)	
**Some high school**	49 (59.8)	45 (49.5)	

**Completed high school**	1 (1.2)	2 (2.2)	
**National certificate**	16 (19.5)	21 (23.1)	
**Some tertiary school**	4 (4.9)	1 (1.1)	
**Completed tertiary**			
Residence			0.07
**Urban/City/Town**	78 (95.1)	79 (86.8)	
**Rural/Countryside**	3 (3.7)	10 (11.0)	
**Unknown**	1 (1.2)	2 (2.2)	
Number of sexual			0.43
partners in last 30 days	37 (45.1)	35 (38.5)	
**≤2**	45 (54.9)	56 (61.5)	
**>2**			
Number of sexual acts in			0.57
last 30 days	38 (46.3)	37 (40.7)	
**≤7**	44 (53.7)	54 (59.3)	
**>7**	0 (0.0)	0 (0.0)	
**Unknown**			
Transactional sex in last			1.00
30 days	34 (41.5)	38 (41.8)	
**Yes**	48 (58.5)	52 (57.1)	
**No**	0 (0.0)	1 (1.1)	
**Unknown**			
Unprotected sex with alcohol use in last 30 days			1.00
**1-2**	26 (31.7)	24 (26.4)	
**3-5**	19 (23.2)	24 (26.4)	
**≥6**	9 (11.0)	13 (14.3)	
**Never**	28 (34.1)	30 (33.0)	
**Unknown**	0 (0.0)	0 (0.00	

[1]*MSM without STIs = MSMs with month 0 STI data, but no STIs in month 0 -24*
[2]*MSM with STIs = MSMs with month 0 STI data and STIs in month 0 – 24*
[3]*Barnard's Exact Test was performed on variables that had 2 categories, while Fisher's Exact Test was performed on variables with 3 or more categories.*

With respect to the STI rates not declining at later time points, could this be due to re-infection (assuming results are being transmitted to participants and treatment is occurring)?

Yes, re-infections were present in some participants. For example, among the 32 MSM with a positive test for chlamydia at multiple time points throughout the 24 months of follow-up, 14 of these were clear re-infections in that they occurred in men who had multiple positive chlamydia tests over time that were separated by at least one negative test.

Table 4: STI reinfections for MSM cohort.

STI	N	[1]Reinf	[2]Not-clear-reinf
CT	32	14	18
NG	9	6	3
CT-NG	3	3	0
Syphilis	6	0	6
	50	23	27

1. Reinf = Clear Reinfection. Multiple Positive results separated by one or more negative results
2. Not-clear-reinf = Not clear reinfection. Multiple positive results with no intervening negative result, so either consecutive positive results, or separated by missing or indeterminate results.
3. There is overlap in people between these categories. By definition, CT-NG co-infection requires both CT and NG infection, and hence CT-NG reinfection implies both CT and NG reinfections. There is also some overlap between CT and NG without coinfection as well as overlap between Syphilis and the other categories. Overall, there are 41 unique participants that account for the 50 participants/STI combinations that have multiple positives across the time-points Months 0, 6, 12, 18, 24

Dear Reviewer #2:

How the authors dealt with loss to follow-up (by Month 24, over 50% of their original sample did not have samples available).

Thank you for mentioning this important point. After non-efficacy was declared for HVTN 702, vaccinations were halted and shortly thereafter follow-up was changed to require one year from the last vaccination received. Fifty seven (57) MSM participants finished their 1-year post-last-vaccination follow-up prior to month 24.The single largest contributor to the missing STI data was the reduction in study follow-up, following the non-efficacy declaration, followed by HIV infection, study dropout, and missed-visits. A complete case analysis was conducted, focusing on the month 0 and 6 time points where missing data was minimal.

Describing the inclusion/exclusion criteria, and diagnostic protocols for women and non-MSM in their study. The authors invest substantial time in describing differences in prevalence between MSM, women and non-MSM men; however, very little attention is paid to describing the comparison group(s) – in particular, which women and non-MSM were swabbed for chlamydia, gonorrhea, and syphilis, and how this may have impacted findings.

The inclusion/exclusion criteria for the primary study has been included as supplementary information. The diagnostic protocols for women and non-MSM have been added to the discussion as follows: All participants born female were tested for gonorrhoea/chlamydia using a urine and vaginal swab. All non-MSM males had to provide only a urine sample. No rectal swabs were collected for non-MSM males. Testing for syphilis using a blood sample for RPR was the same across all study groups. Due to differences in the sample collections, only positive results are analyzed in this study irrespective of the site/location of infection.

Suggestions and Comments

Abstract: Results – says 183 participants who identified as male or transgender female & of homosexual or bisexual orientation. But in Background, the authors state that their goal was to compare rates to females and non-MSM males. Should clarify that they compared results of the 183 MSM to the rest of the participants in a larger trial.

The Background statement is been changed to: We describe bacterial STI prevalence amongst MSM enrolled in an HIV vaccine efficacy trial in South Africa, and compared these results to the STI prevalence of the rest of the participants in the larger trial.

Introduction

Lines 41-42: can the authors comment on the prevalence of STIs in non-MSM populations – this comparison can help bolster their argument regarding the excess burden of STIs among MSM.

The following has been added to Line 44 – 46: In comparison, there were 97,450 new STI diagnoses amongst non-MSM males. Chlamydia was more common amongst non-MSM males (n= 46,192; 47.4%) followed by NG (n= 15,253; 15.7%). There were 1,016 (1%) new syphilis infections.

Line 45: - would suggest quantifying what "majority" means.

This line now reads: The number of new gonorrhea cases diagnosed in 2019 (n= 70,936) was the largest annual number reported since records began in 1918 and 33 853 (47.7%) of those cases were reported in MSM [5].

Lines 47-48: While I don't disagree with this assessment, the authors should back this assertion with some references. Additionally, there has not been a complete lack of research in sub-Saharan Africa (SSA), so I'd suggest a more thorough literature review on presentation of STI rates in the SSA context. As well, this would be a good place to specify some of the reasons behind the lack of STI research in the SSA context. For example, the high costs of diagnostics, laboratory capacity, barriers to access and accessibility – including specific stigma against MSM in sub-Saharan countries.

Thank you for this suggestion. Literature review was done and 2 articles have been referenced.

There is poor health-seeking behavior amongst MSM within South Africa and other Sub-Saharan countries, as the fear of stigma and discrimination by healthcare workers poses as a barrier to access HIV/STI services [6, 7]. The criminalization of homosexuality in some African countries also poses as a barrier to seeking healthcare services [7]. Political, social and religious factors play an important role in

limiting research in this key population. Estimating the burden of STIs in MSM is therefore a huge challenge.

Methods
Line 58: Would suggest "The trial enrolled participants from October 2016…with the study protocol specifying…"

The line has been changed to: The trial enrolled from October 2016 to June 2019 with the study protocol specifying enrolment of 30-35% men.

The authors do not report any information regarding data collection and analysis among females and non-MSM males. In fact, from Line 78 onwards, a reader would be lead to believe that only data on MSM were analysed. This needs to be clarified, as a comparison to females and non-MSM participants from the larger trial comprises a critical component of the manuscript. Others have found higher prevalence of STI in rectal samples from MSM (Jones et al, 2020. JIAS Vol. 23, S6) in South Africa; thus without knowledge of how samples are obtained from women and (to a certain extent non-MSM), this may not be exactly an apples-to-apples comparison.

Thank you for raising this point. The primary focus of this manuscript is to describe the STI prevalence amongst MSM. However, a commentary is made on how these findings compared to other participants in the larger trial i.e. females and non-MSM males.

It looks like the authors ignored indeterminate results (Table 3) – this needs to be specified in the Methods section, especially given the lower absolute number of infections, and their large lost to follow-up.

Indeterminate STI results were considered as missing data. This has been added to the Methods section.

Lines 95-96: With respect to the generalized estimating equations model – was this a logistic regression? And what is the rationale for using GEE as opposed to a simpler method, such as McNemar's test especially if the authors are only comparing 2 points in time. Relatedly, did the authors consider using GEE across all points in time where data were available? If not, why not?

Yes, a generalized estimating equation model with logit link was used. The model held appeal for assessing differences across time however the rates of missing data at the later time points were problematic for the missing at random assumption of GEE. McNemar's test could be used but is expected to be less powerful.

As this was an analysis of data from an HIV vaccine trial, I assume that ethics and informed consent covered publication of results from secondary outcomes?

You are correct. All relevant data used for this analysis will be available as supplementary information.

Results
Lines 126-132: Again, a reminder to the authors that a clarification regarding comparisons to females and non-MSM males is strongly suggested. As well, what happens to differences upon adjustment by age, region, etc.?

As the main focus of the manuscript is on describing STIs among MSM throughout the trial, comparisons with females and non-MSMs are not included here.

Table 2: 95%CI would be useful here.

Thank you for this suggestion. 95%CI have been added to Table 2.

Additionally, by month 24 less than half of their original study (or only 82 of the original 173) were not available for analysis. What were the reasons for availability/unavailability? This becomes crucial when trying to interpret results, as prevalence is impacted by dropouts. More information is needed from authors on how they dealt with this issue.

Please see response to reviewer #1.

Discussion
Subanalyses: did the authors examine who cleared CT infections, and who did not? It is interesting that CT declined from Month 0 to Month 6, but GC did not. This might suggest some natural clearing of CT or perhaps false positives in Month 0.

Testing for clearing of CT infections was not done as the protocol did not require re-testing post treatment. Behavioral risk analysis was administered to participants with positive STI results. The drop in CT infections is likely due to participants incorporating safer sex practices. However, as the study progressed, these practices were later abandoned. There are no laboratory records of false positive STI results.

Lines 184-186: Why did the authors not examine whether these factors were associated with STI prevalence?

Thank you for this question. Please see response to reviewer 1.

Limitations – were syphilis tests able to discern acute infections from historical ones?

Yes. All positive RPR results were accompanied by RPR titres. The titres were used to determine the presence of active (require treatment) vs historic ones (no action).
