# Supplementary material for: Sexually transmitted infections amongst men who have sex with men (MSM) in South Africa
Source: PLOS Glob Public Health. 2023 Apr 5;3(4):e0001782. doi: 10.1371/journal.pgph.0001782 (PMC10075439; doi:10.1371/journal.pgph.0001782)
Supplement: Attachment — Submitted filename: Rebuttal letter.pdf [file pgph.0001782.s003.pdf]

Manuscript: PGPH-D-22-00629R2

Sexually transmitted infections amongst men who have sex with men (MSM) in South Africa
PLOS Global Public Health

Dear Reviewer #3

Thank you for taking time to review this manuscript. Please see below responses to your comments/suggestions.

This revised manuscript describes changes over time in STI prevalence during follow-up in a clinical trial aimed at evaluating efficacy of an HIV vaccine in South Africa. Data of this kind are not common in sub-Saharan Africa. The authors responded adequately to the reviewers' comments; however, there are some outstanding issues worth addressing.

The authors only provide a statistical comparison of STI prevalence based on months 0 and 6 (justified by the fact that very few data were missing at these time points). Yet, they report STI prevalence up until month 24 and infer on changes in STI prevalence during the 24-month period. There needs to be consistency here. Please report STI prevalence and test for changes in this prevalence for the same follow-up period. If the authors are so concerned with violating the MCAR assumption of the GEE model, why not use a different model that relaxes this assumption (e.g., mixed effect model with random intercept)? (As an aside, I do agree with the authors that modeling STI incidence would be somewhat difficult without a follow-up time prior to baseline.)

Response: Thank you for this comment. Our main interest is in assessing differences in STI prevalence at baseline (month 0) and at the first post-baseline time point. Any difference in STI prevalence is expected to be most pronounced between baseline and the first STI assessment due to behavioral inhibition following the risk reduction counselling received on study, as seen in other efficacy trials [11, 12, 13]. In addition, substantial missing STI data beyond the first two time points poses a challenge to the analysis. We have revised the text throughout the manuscript to only refer to changes at the first two time points.

The authors seem to have data on behaviors during follow-up (ln 123-129). If so, it is unclear why they did not also evaluate the changes in behavior over time to corroborate their findings of changes in STI prevalence. These need to be added to the analysis, if available.

Response: Risk behavior data was collected throughout the trial but not at the same time points as the STI data collection. For example, testing for Neisseria gonorrhoea (NG) and Chlamydia trachomatis was performed at month 6 but risk behavior was collected at month 6.5. As the manuscript focuses on the assessment of differences in STI prevalence at the first two time points, we evaluated whether demographic and month 0 risk behavior variables differed between MSMs with and without STIs at months 0 and 6. These results are reported in Table 4 and we have added a description of this analysis to the Statistical Analysis section.

The analysis on risk factors for STI is ambiguous (Table 5) and the footnotes do not adequately describe what was being modeled as an outcome. Any STI within 24

months? And risk factors evaluated at which time points (as the authors state "between months 0-24", ln 148)? If the covariates were time-updated, they could simply model the outcome STI at each time point along with corresponding covariates measured at the same time points. Techniques to account for repeated measured (i.e., GEE or mixed-effects) could be applied accordingly. Regardless, this analysis needs to be described in more detail in the statistical analysis section.

Response: Thank you for pointing out that this analysis could be better clarified. We have added a description to the Statistical Analysis section and revised Table 4 (formerly Table 5) to report and compare the rates of demographic and risk behaviour characteristics at baseline among MSMs with at least one STI at months 0 and/or 6 vs. those without an STI at months 0 and 6. Information about the MSM groups now appears in the column headers rather than in footnotes to the table and we restrict to STIs at the first two time points which are of greatest interest and contain the most complete data.

The reviewers asked for more clarity in the statistical analysis section and the authors did not accommodate these requests accordingly (even though their answerers were sufficient). Please state the endpoint being modelled. This is a logistic regression model with general estimating equations; please state as such (if this model was indeed entertained).

Response: Additional detail has been added to the Statistical Analysis section to clarify that STI prevalence was modelled as a binary endpoint using general estimating equations with a logit link and independent correlation structure.

One final general comment: please adhere to the STROBE reporting guidelines. There are several items that appear to be missing in this manuscript.

Response: Thank you for the suggestion to align to STROBE, which we have done as far as possible. We have improved clarity in the manuscript that we performed a retrospective analysis on clinical trial data, i.e. HVTN 702 was not an observational study. We changed the Abstract to read:

Our retrospective analysis used data from the HVTN 702 HIV vaccine efficacy clinical trial (October 2016 to July 2021).

Manuscript: PGPH-D-22-00629R2

Sexually transmitted infections amongst men who have sex with men (MSM) in South Africa
PLOS Global Public Health

Minor comments:

ln 14. Please provide totals for all groups along with their median (IQR) follow-up time. (Potential for differential LTFU should also be assessed in the limitations section.)

Response: The totals and follow-up times have been added to line 14. Abstract now reads:

The clinical trial also enrolled and performed month 0 STI testing on 3389 female participants, median age 23 (IQR 21-27) years, median 24.8 (IQR 18.8, 24.8) months FU and 1080 non-MSM males with a median age of 27 (IQR 24-31) years, median 24.8 (IQR 23, 24.8) months FU. The primary follow-up period was 24 months.

We have added comments about differential LTFU. Line 268 – 273 now reads:

Following non-efficacy declaration, there was a reduction in study follow up visits. This was the largest contributor to the reduction in number of participants and STI data over time. HIV infection, study dropouts and missed visits also contributed to the loss of study participants and data. There is potential for differential loss to follow up (LTFU) bias as participants who either tested positive for HIV or an STI dropped out of the study. This would result in an underestimation of the prevalence of STIs at later time points.

ln 52. What type of "health-seeking behavior" is being referenced here? Also, there are no citations to support this claim.

Response: Thank you for this question. Citation has been added. Line 52 now reads:

There is poor health-seeking behavior (accessing HIV/STI screening and testing services, and disclosure of sexual practices to healthcare workers) amongst MSM within South Africa and other Sub-Saharan countries [6]

ln 58. In what ways are the "profiles" being described? "changes in STI prevalence" would be more appropriate.

Response: We agree and have replaced "profiles" with "prevalence", avoiding the mention of changes in prevalence as these are only considered for the first two time points whereas prevalence is described for the entire follow-up period. The sentence has been changed to "In our analysis, we describe the STI prevalence amongst MSM enrolled in a preventive HIV vaccine trial which had routinely tested for sexually transmitted infections as part of study procedures"

Manuscript: PGPH-D-22-00629R2

Sexually transmitted infections amongst men who have sex with men (MSM) in South Africa
PLOS Global Public Health

Table 1. Why include a column on all MSM? Since the main outcome is STI prevalence, would it be more obvious to exclude those without an STI measurement? Or did those without STI measured at month 0 have an STI measured at a later time point?

Response: STI testing was added under version 2 of the protocol with STI testing initiated on 08 May 2017. Participants enrolled under version 1 of the protocol did not have STI testing performed. This resulted in 10 MSM participants not being tested for STI at month 0. However, these participants did receive STI testing at later time points. Table 1 includes a column for all MSM to indicate that demographics are similar among all MSM and MSM with STI testing at month 0.

The inclusion and exclusion criteria could be more thoroughly described.

Response: Thank you for this suggestion. Line 75-77 now reads: To be enrolled into HVTN 702, participants had been assessed as being healthy, 18-35-year-old HIV-uninfected adults at risk of HIV acquisition who were willing to receive HIV counselling and testing. The final decision to either include or exclude a volunteer from the study was based on laboratory test results, medical history, physical examination findings and answers to questionnaires. Possible barriers to retention e.g. employment were also considered.
The detailed inclusion/exclusion criteria is provided in the supplementary section

Table 3 is excessive. The authors could very well add a sentence, such as: "XX CT infectious were observed in NN individuals." to begin the description of prevalence over time.

Response: Thank you for your comment about Table 3. Table 3 has been deleted. Line 149 - 151 now reads, Overall, there were 41 unique participants that accounted for the 50 participants/STI combinations that had multiple positives across the time-points Months 0, 6, 12, 18, 24.

ln 252-256. The authors seem to be missing the point of the reviewer. Normally, if you test in more places, you are more likely to find an STI. Is there a differential diagnostic bias between MSM, females at birth and non-MSM? For example, if the non-MSM never engaged in insertive anal sex with a male partner, their risk of having an anal STI is essentially nil, hence no reason to test for rectal STIs. The potential for differential diagnostic bias would likely depend on whether MSM was correctly classified in this instance. Please modify the discussion accordingly.

Response: Thank you for your comment. The Discussion now reads this:

For our analysis, CT and NG results among MSM were each considered positive if either the urine or rectal sample testing was positive. Urine testing for non-MSMs was regarded as sufficient for detection of STIs in this population. Rectal testing was excluded in non-MSMs,

as the risk of receptive anal sex with a male partner was considered nil based on their behavioral questionnaires. CT and NG testing amongst females was done on both urine and vaginal swabs.

Due to differences in sexual practices amongst MSM, females and non-MSMs, including the specific anatomical site for the positive test was therefore not a requirement for our analysis.

However, the difference in diagnostic tests in each group is a potential for differential diagnostic bias. This is likely to affect those non-MSMs who self-identified as heterosexual and failed to disclose engaging in receptive anal sex with a male partner for fear of being labeled gay or MSM. Failure to do rectal testing in these participants would result in missed/undiagnosed STIs.
